# Supplementary material for: Disentangling cell-intrinsic and cell-extrinsic factors underlying evolution
Source: Cell Genom. 2025 May 29;5(8):100891. doi: 10.1016/j.xgen.2025.100891 (PMC12366658; doi:10.1016/j.xgen.2025.100891)
Supplement: Document S1. Figures S1–S29 and Tables S4 and S5 [file mmc1.pdf]

Cell Genomics, Volume *5*

Supplemental information

Disentangling cell-intrinsic

and cell-extrinsic factors underlying evolution

Alexander L. Starr, Toshiya Nishimura, Kyomi J. Igarashi, Chihiro Funamoto, Hiromitsu Nakauchi, and Hunter B. Fraser

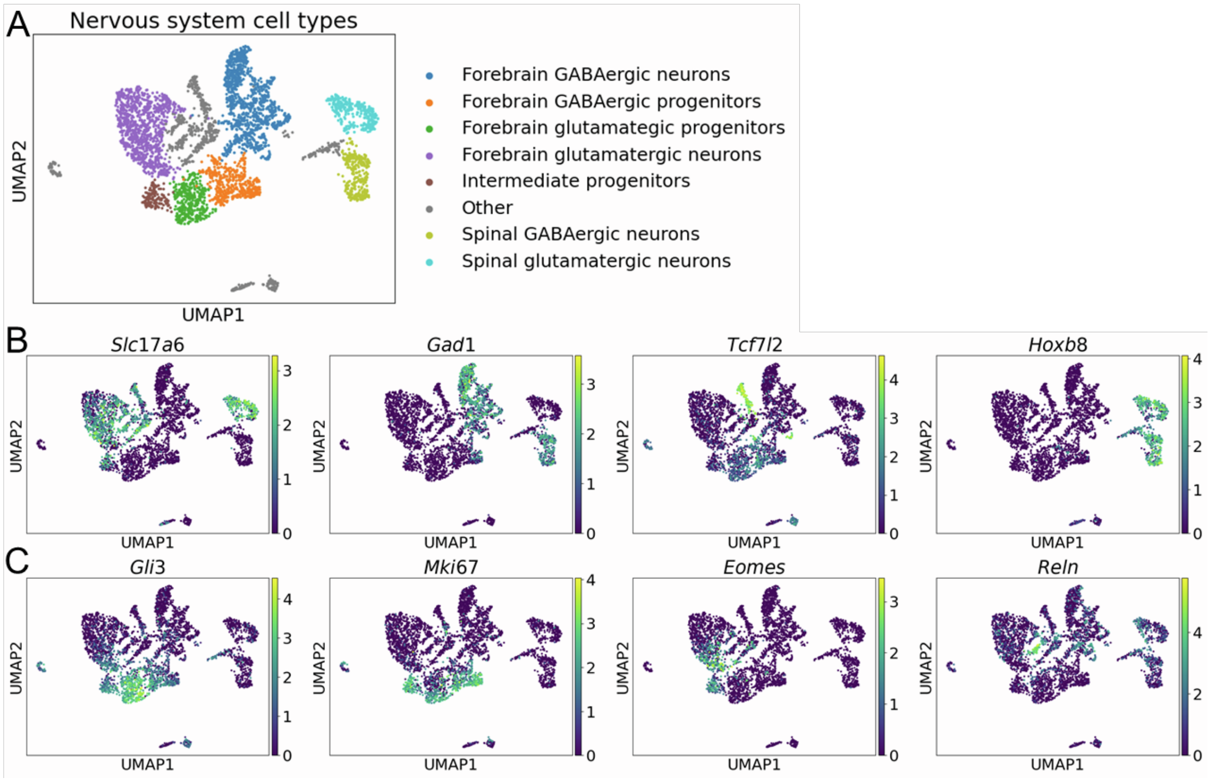

Fig. S1: Nervous system cell type annotations, related to figure 3. A) Uniform manifold approximation (UMAP) of all nervous system cell types. Cell types analyzed in this study are labeled and all other cell types are categorized as "Other". **B)** Marker genes used to classify nervous system cell types. From left to right: expression of *Slc17a6*, a marker of glutamatergic cells, expression of *Gad1,* a marker of GABAergic cells, expression of *Tcf7l2*, a marker for midbrain neurons (which were not analyzed), expression of *Hoxb8*, a marker for spinal neurons. Each point is colored by the log normalized expression in the cell represented by the point. **C)** Additional marker genes used to classify cell types. From left to right: expression of *Gli3*, which is highly expressed in forebrain glutamatergic progenitors, expression of *Mki67*, a marker of cycling cells, expression of *Eomes*, a marker for intermediate progenitors, expression of *Reln*, a marker of Cajal-Retzius cells which were excluded from further analysis.

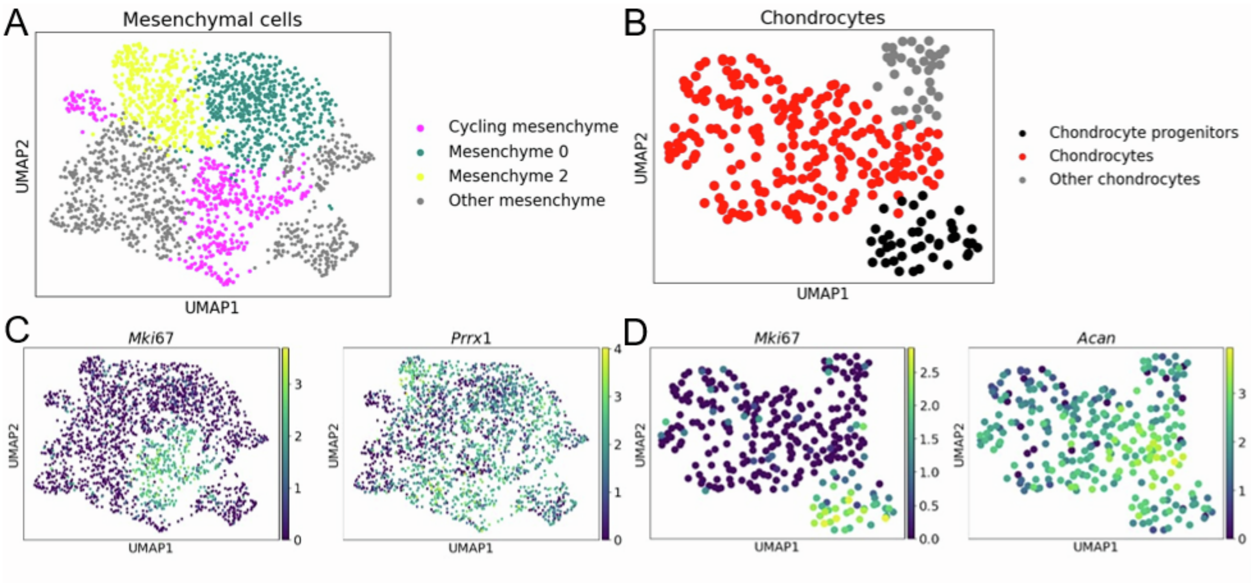

Fig. S2: Connective tissue cell type annotations, related to figure 3. A) UMAP of mesenchymal cell subtypes. UMAP is used purely for visualization and the proximity of different clusters in UMAP space cannot be used as a proxy for the true similarity between groups of cells. The small group of cycling mesenchymal cells with lower *Mki67* expression in the upper left is most similar in gene expression to the large group of cycling mesenchymal cells with higher *Mki67* expression as determined by leiden clustering and so are included with that cluster. Some cell types excluded from further analysis are labeled as Other. **B)** UMAP of chondrocyte subtypes. Cycling chondrocytes and other chondrocytes were excluded from further analysis. **C)** Expression of *Mki67*, a marker of cycling cells, and *Prrx1*, a marker of mesenchymal cells, in mesenchymal cells. Each point is colored by the log normalized expression in the cell represented by the point. **D)** Expression of *Mki67* and *Acan*, a marker of chondrocytes, in chondrocytes.

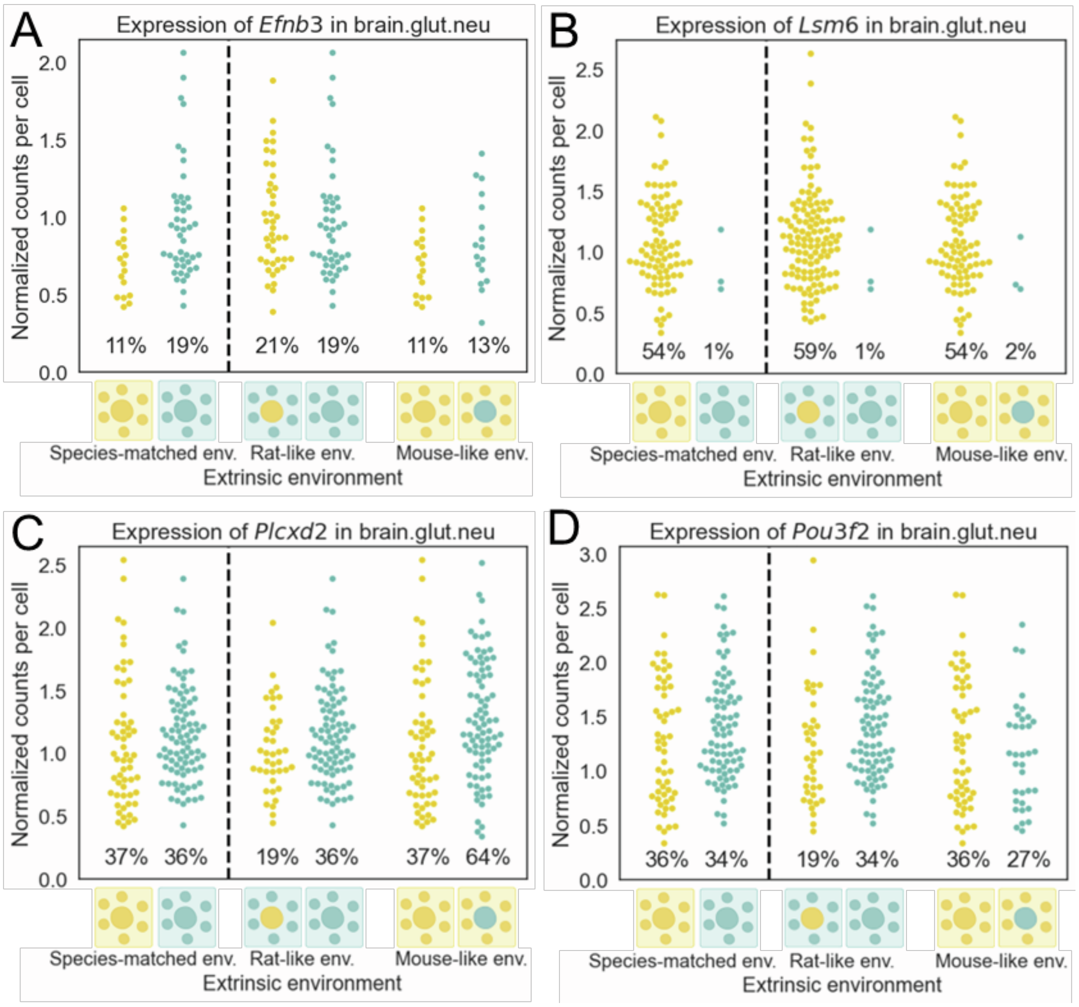

Fig. S3: Per-cell expression distribution for example genes, related to figure 3. Each swarm of points shows the normalized counts for a gene in each forebrain glutamatergic neuronal cells with non-zero counts for that gene. The percentage near the bottom of the plot indicates the percentage of cells with non-zero counts for that gene. A) Per-cell expression for *Efnb3*. B) Per-cell expression for *Lsm6*. C) Per-cell expression for *Plcxd2*. D) Per-cell expression for *Pou3f2*.

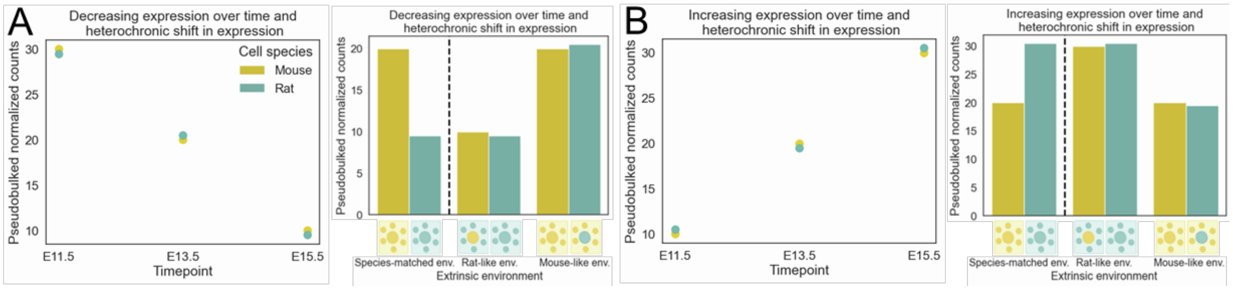

Fig. S4: Conceptual outline of the interplay of intrinsic divergence and heterochronic shifts in gene expression, related to figure 3. An important aspect of gene expression we have not explored in this study is changes in gene expression over developmental time (developmentally dynamic expression). In addition, the trajectory of gene expression can diverge between species. To explore how developmentally dynamic expression influences the results presented here, we analyzed bulk RNA-seq data from a time course of mouse and rat development, focusing on E11.5, E13.5, E15.5 in mouse and E11, E13, and E15 in rat. We analyze how a combination of intrinsic divergence and developmentally dynamic gene expression can appear as having purely interaction, purely extrinsic, or reinforcing/opposing extrinsic and intrinsic divergence in our study. First, a “temporal shift” (i.e. a shift against the global shift in gene expression associated with slower development of rats compared to mice) in gene expression could appear as extrinsic divergence in our study. As mentioned above, rat development proceeds at a slower pace than mouse development. Therefore, if there is no divergence in the expression trajectory of a gene beyond this global change, we would expect rat expression to differ from mouse expression at identical (but not stage-matched) timepoints for developmentally dynamic genes. However, the expression trajectories of some genes might not shift with this global change. This would result in the same expression trajectory (i.e. increasing or decreasing) in both species, but with similar expression levels at identical time points. **A)** Expression of a hypothetical gene across development. The gene decreases in expression over development but has very similar expression in mice and rats at the same

embryonic timepoints, going against the global difference in developmental rate between species. In the absence of extrinsic or interaction divergence, this gene would appear to have purely extrinsic divergence and higher expression in a mouse-like environment. **B)** The same as in (A) but showing a gene with increasing expression over time and a heterochronic shift. In the absence of extrinsic or interaction divergence, this gene would appear to have purely extrinsic divergence and higher expression in a rat-like environment.

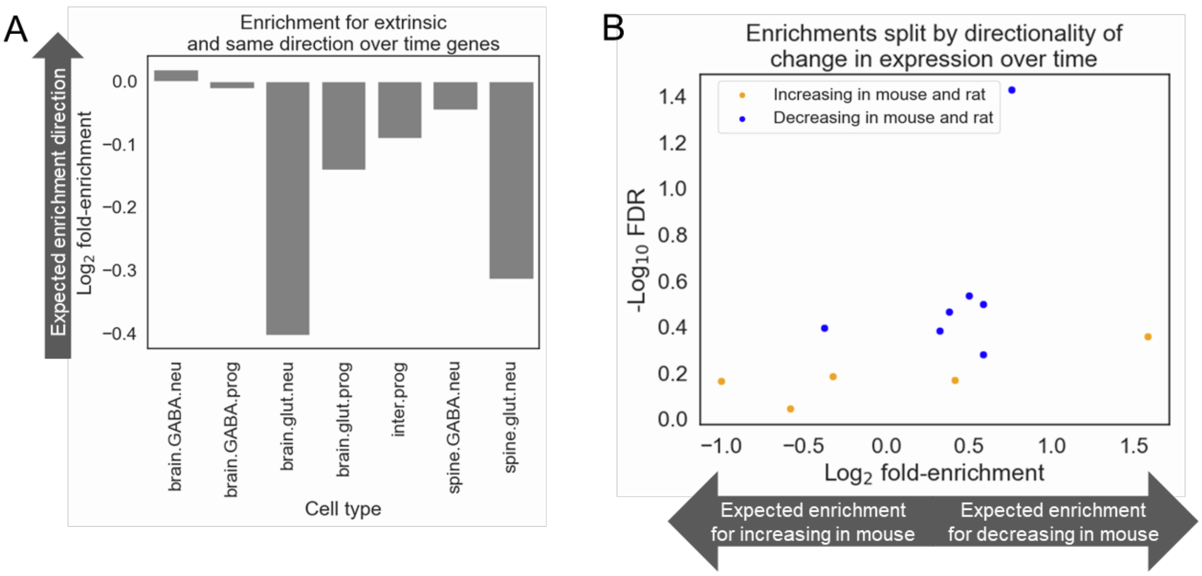

Fig. S5: Enrichment analysis for heterochronic shift genes, related to figure 3. See Methods for how heterochronic shift genes were defined. If a temporal shift occurs through purely intrinsic mechanisms, we would observe purely extrinsic divergence in our study. To test whether this occurs frequently, we identified genes with similar expression trajectories in mice and rats but small differences in expression at both E13.5 and E15.5 (absolute log fold-change less than 0.25). Importantly, when adding the restriction that the log fold-change in expression between E13.5 and E15.5 within species be greater than 0.5, less than 1% of genes fulfill these criteria suggesting the kind of temporal shift in expression hypothesized here is very rare. Therefore, we proceeded only with the restriction of low divergence between species at identical timepoints and similar expression trajectories between species. **A)** Enrichment analysis for genes with similar expression trajectories during embryonic brain development and similar expression levels at E13.5 and E15.5 in mice and rats and proportion extrinsic divergence. The y-axis shows the $\log_2$ fold-enrichment and the x-axis corresponds to cell type. The arrow shows the expected enrichment if intrinsic heterochronic shift genes were inflating estimates of extrinsic divergence. Overall, we find weak or no enrichment for genes with high proportion extrinsic divergence in this set of genes with evidence for a temporal shift (no cell types with

enrichments in the expected direction with $p < 0.1$). **B)** In addition, we would expect that temporal shift genes that decrease over time would be enriched for negative extrinsic divergence and vice versa (Fig. S4A-B). Enrichment analysis for genes with similar expression trajectories during embryonic brain development and similar expression levels at E13.5 and E15.5 in mice and rats and signed proportion extrinsic divergence. The x-axis is the $\log_2$ fold-enrichment and the y-axis is the $-\log_{10}$(FDR). The arrow shows the expected enrichment if intrinsic heterochronic shift genes were inflating estimates of extrinsic divergence. Each point corresponds to the enrichment in a central nervous system cell type and each cell type is represented twice, once as an blue dot for genes that are decreasing in mouse and rat (and have similar expression levels at E13.5 and E15.5 in mouse and rat) and once as an orange dot for genes that are increasing in mouse and rat (and have similar expression levels at E13.5 and E15.5 in mouse and rat). Here again we find only weak enrichments.

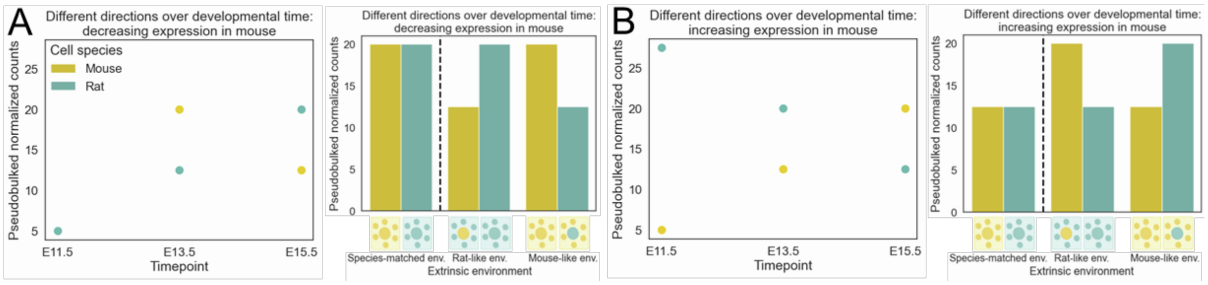

Fig. S6: Conceptual outline of the interplay of intrinsic divergence and switches in the trajectory of gene expression during development between species, related to figure 3. Another form of temporal divergence in gene expression is when the expression of a gene increases over time in one species but decreases over time in another species. For example, if a gene is intrinsically decreasing over time in mouse and increasing over time in rat (or vice versa), this could appear as purely an interaction between extrinsic and intrinsic divergence in our study. **A)** Expression of a hypothetical gene across development. The gene increases in expression over time in rats, but decreases in expression over time in mice. In the absence of extrinsic or interaction divergence, this gene would appear to have purely interaction divergence and higher expression in species-matched environments. **B)** The same as in (A) but showing a gene with increasing expression over time in mice and decreasing expression over time in rats. In the absence of extrinsic or interaction divergence, this gene would appear to have purely interaction divergence and higher expression in species-mismatched environments.

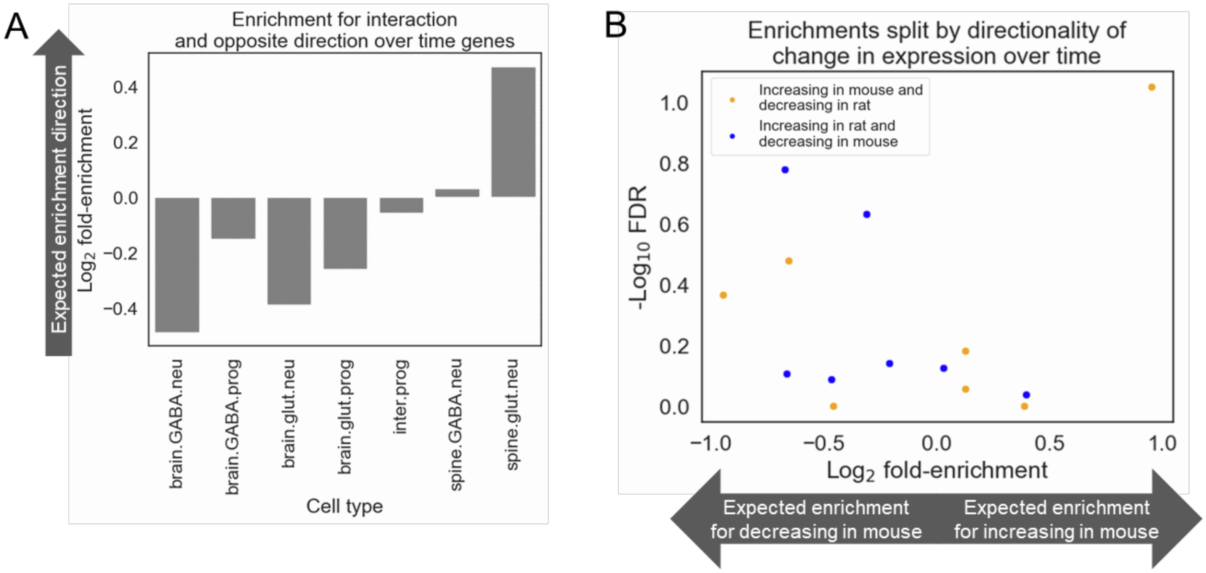

Fig. S7: Enrichment analysis for genes with switched trajectories of gene expression during development in mice and rats, related to figure 3. See Methods for how genes with switches in gene expression trajectory between species were defined. If this genes with intrinsically opposite expression trajectories contribute to the inflation of the interaction component in our study, we would expect that genes with high interaction proportions would be enriched for opposing trajectories in the time course data. **A)** Enrichment analysis for genes with switches in gene expression during embryonic brain development and proportion interaction divergence. The y-axis shows the $\log_2$ fold-enrichment and the x-axis corresponds to cell type. The arrow shows the expected enrichment if genes with the opposite gene expression trajectory between mice and rats were inflating estimates of interaction divergence. Across all brain cell types in our study, we find little to no evidence for this enrichment ($p < 0.1$ in one cell type). **B)** In addition, we can make a stronger prediction about the sign of interaction divergence if this confounder plays a major role. If gene expression is decreasing in mouse and increasing in rat, then this would lead to higher expression in species-matched environments (i.e. negative interaction divergence) due to the slower development of rat cells (Fig. S6A). As a result, this category of genes should be enriched for negative interaction divergence. Similarly, genes that

increase in expression over time in mouse but decrease over time in rat would lead to higher expression in species-mismatched environments (i.e. positive interaction divergence, Fig. S6B). This category should then be enriched for positive interaction divergence. Enrichment analysis for genes with switches in gene expression during embryonic brain development between species and signed proportion interaction divergence. The x-axis is $\log_2$ fold-enrichment and the y-axis is the $-\log_{10}$(FDR). The arrow shows the expected enrichment if genes with the opposite gene expression trajectory between mice and rats were inflating estimates of interaction divergence. Each point corresponds to the enrichment in a central nervous system cell type and each cell type is represented twice, once as a blue dot for genes that are increasing over time in rat and decreasing over time in mouse, and once as an orange dot for genes that are decreasing over time in rat and increasing over time in mouse. We find weak to no enrichment in the expected direction ($p < 0.1$ for one cell type for increasing in mouse, no cell types for decreasing in mouse).

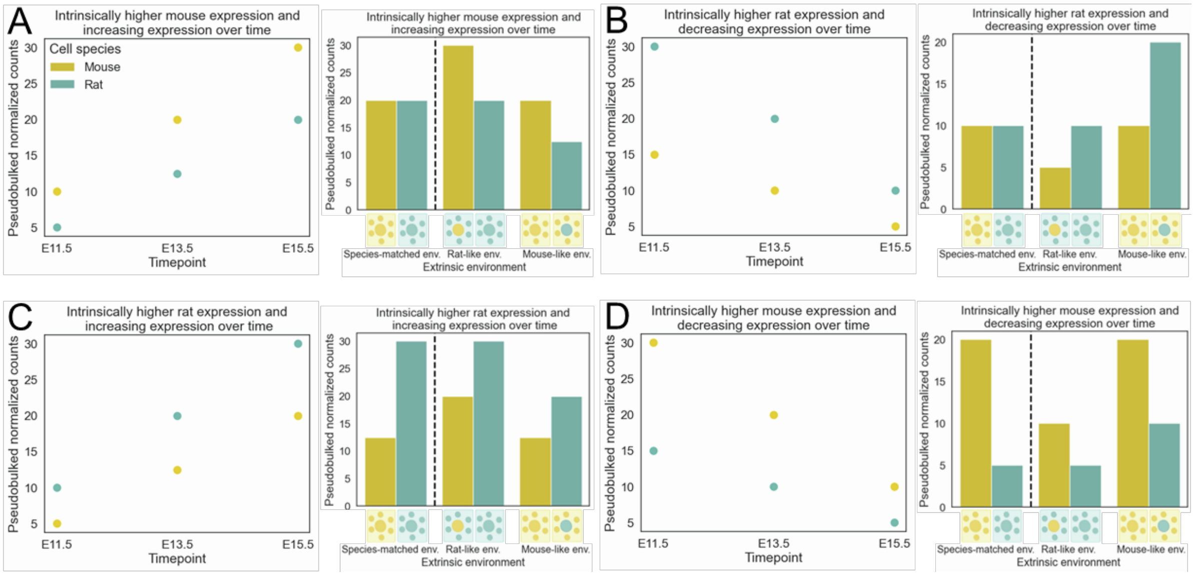

Fig. S8: Conceptual outline of the interplay of intrinsic divergence and conserved gene expression trajectories, related to figure 3. Intrinsic divergence coupled with a conserved expression trajectory can lead to the appearance of opposing or reinforcing extrinsic or intrinsic divergence. For example, if a gene is increasing in expression over time in both species and is intrinsically more highly expressed in mouse cells, this can appear as opposing extrinsic and intrinsic divergence in our study. In general, we would expect that both opposing and reinforcing genes would be enriched for genes with conserved expression trajectories in mouse and rat development. **A)** Expression of a hypothetical gene across development. The gene increases in expression over development in both mice and rats, but is intrinsically more highly expressed in mice. In the absence of extrinsic or interaction divergence, this gene would appear to have opposing extrinsic and intrinsic divergence. **B)** The same as in (A) but showing a gene with decreasing expression over time in both species and intrinsically higher expression in rat cells. In the absence of extrinsic or interaction divergence, this gene would appear to have opposing extrinsic and intrinsic divergence. **C)** The same as in (A) but showing a gene with increasing expression over time in both species and intrinsically higher expression in rat cells. In the

absence of extrinsic or interaction divergence, this gene would appear to have reinforcing extrinsic and intrinsic divergence. **D)** The same as in (A) but showing a gene with decreasing expression over time in both species and intrinsically higher expression in mouse cells. In the absence of extrinsic or interaction divergence, this gene would appear to have reinforcing extrinsic and intrinsic divergence.

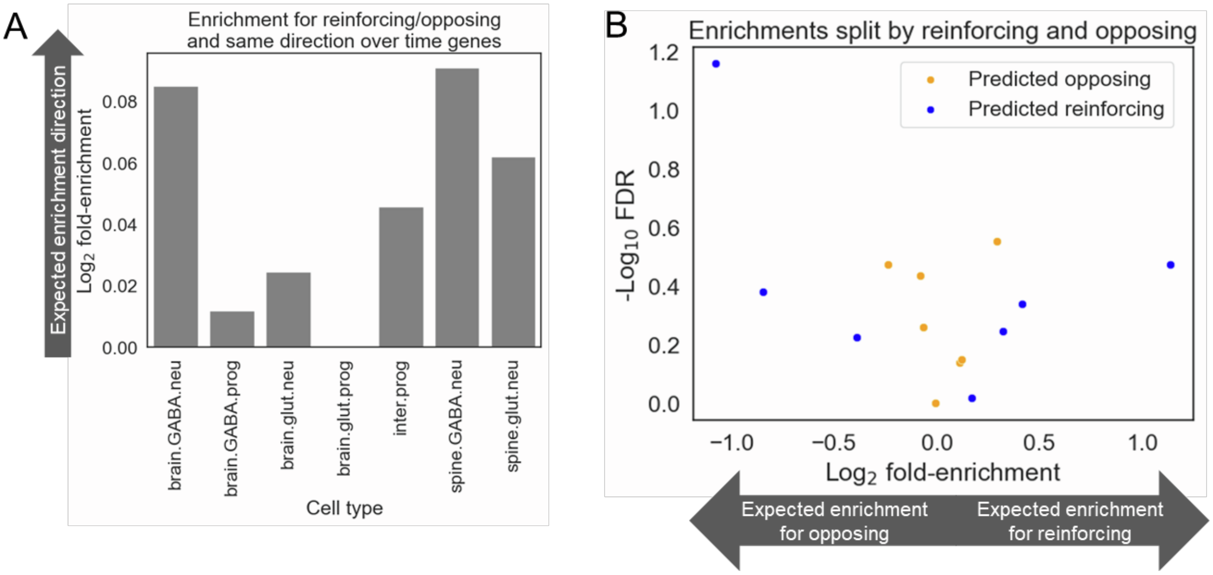

Fig. S9: Enrichment analysis for genes with conserved gene expression trajectories and reinforcing/opposing intrinsic and extrinsic divergence, related to figure 3. See Methods for how genes with conserved expression trajectories between species were defined. We can make the more specific hypothesis that genes that are intrinsically higher in mouse cells and increasing over time as well as genes that are intrinsically higher in rat and decreasing over time should be enriched for opposing extrinsic and intrinsic divergence (Fig. S8A-B). On the other hand, genes that are intrinsically higher in mouse cells and decreasing over time as well as genes that are intrinsically higher in rat and increasing over time should be enriched for reinforcing genes (Fig. S8C-D). **A)** Enrichment analysis for genes with conserved expression trajectories between species and the product of intrinsic proportion divergence and extrinsic proportion divergence. For this metric, genes with high values have opposing or reinforcing expression and genes with low values do not. The y-axis shows the $\log_2$ fold-enrichment and the y-axis corresponds to cell type. The arrow shows the expected enrichment if genes with conserved expression trajectories between mice and rats were inflating estimates of reinforcing/opposing divergence. **B)** Enrichment analysis for genes with conserved expression trajectories between species and the product of signed intrinsic proportion divergence and

signed extrinsic proportion divergence. For this metric, larger positive values indicate reinforcing intrinsic and extrinsic divergence, large negative values indicate opposing intrinsic and extrinsic divergence, and values near zero indicate neither reinforcing nor opposing intrinsic and extrinsic divergence. The x-axis is the $\log_2$ fold-enrichment and the y-axis is the $-\log_{10}$(FDR). The arrow shows the expected enrichment if genes with the conserved gene expression trajectories between mice and rats were inflating estimates of reinforcing/opposing intrinsic and extrinsic divergence. Each point corresponds to the enrichment in a central nervous system cell type and each cell type is represented twice, once as a blue dot for genes that are increasing over time in mouse and rat so would be predicted to appear as having reinforcing extrinsic and intrinsic divergence in our study, and once as an orange dot for genes that are decreasing over time in mouse and rat so would be predicted to appear as having opposing extrinsic and intrinsic divergence in our study. We find limited evidence for either hypothesis. Although this suggests that intrinsic divergence coupled with similar changes in expression over time is not the primary contributor to opposing or reinforcing extrinsic and intrinsic divergence, there are many genes with conserved expression trajectories suggesting that this area in particular should be explored further in future studies. Overall, these results suggest that a combination of intrinsic divergence and developmentally dynamic expression do not overly inflate our estimates of extrinsic and interaction divergence. However, we have only discussed intrinsic divergence in conjunction with developmentally dynamic gene expression. Various other complex combinations of divergence can instead inflate estimates of intrinsic divergence. For example, extrinsic divergence itself can be partially responsible for global shifts in the trajectory of gene expression further adding to complexity. Data from developmental time courses in reciprocal chimeras will undoubtedly provide valuable insight into how intrinsic, extrinsic, and temporal divergence interact and be vital in developing a more complete understanding of the molecular mechanisms underlying gene expression divergence.

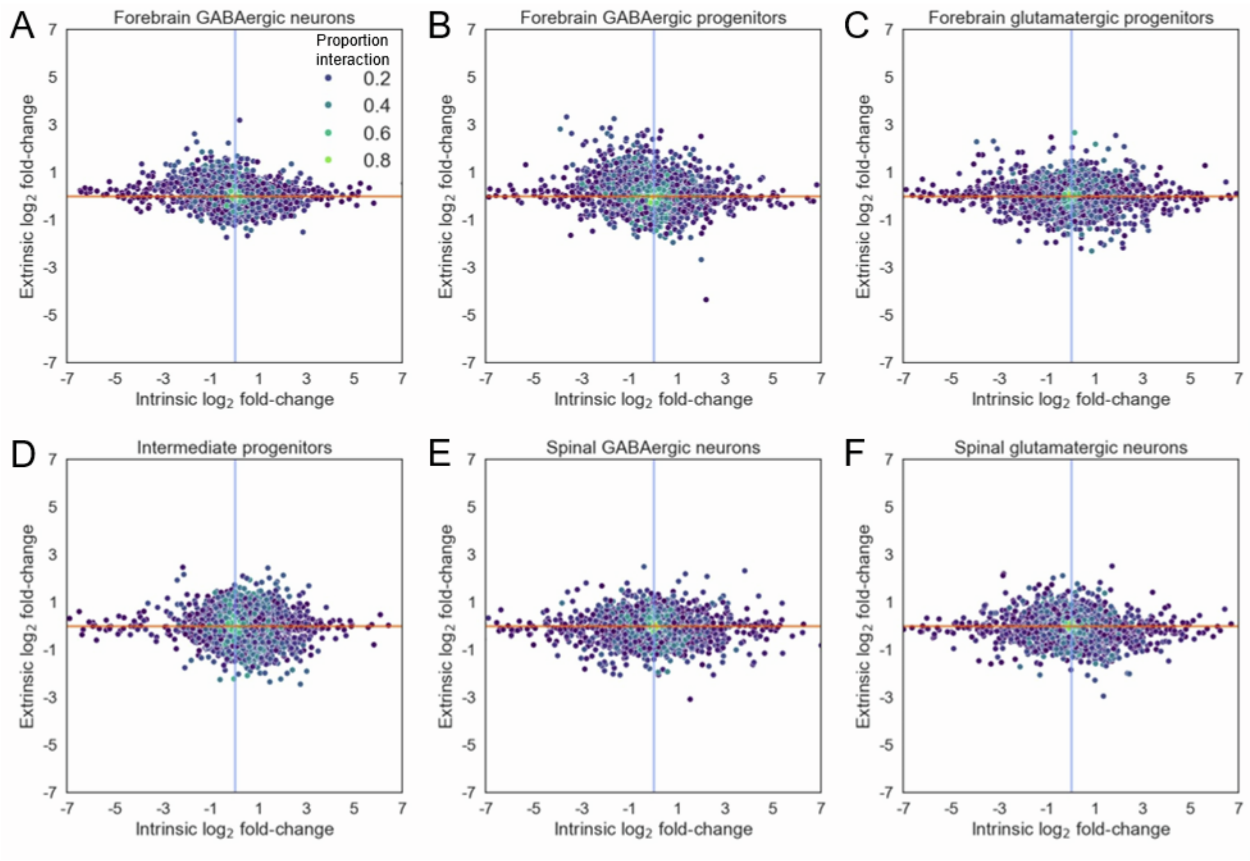

Fig. S10: Intrinsic and extrinsic divergence of nervous system cell types, related to figure 3. Each point is a gene. Intrinsic divergence is on the y-axis and extrinsic divergence is on the x-axis. Genes are colored by their proportion interaction. Forebrain glutamatergic neurons are shown in Fig. 2F. **A)** Plot for forebrain GABAergic neurons. **B)** Plot for forebrain GABAergic progenitors. **C)** Plot for forebrain glutamatergic progenitors. **D)** Plot for intermediate progenitors. **E)** Plot for spinal GABAergic neurons. **F)** Plot for spinal glutamatergic neurons.

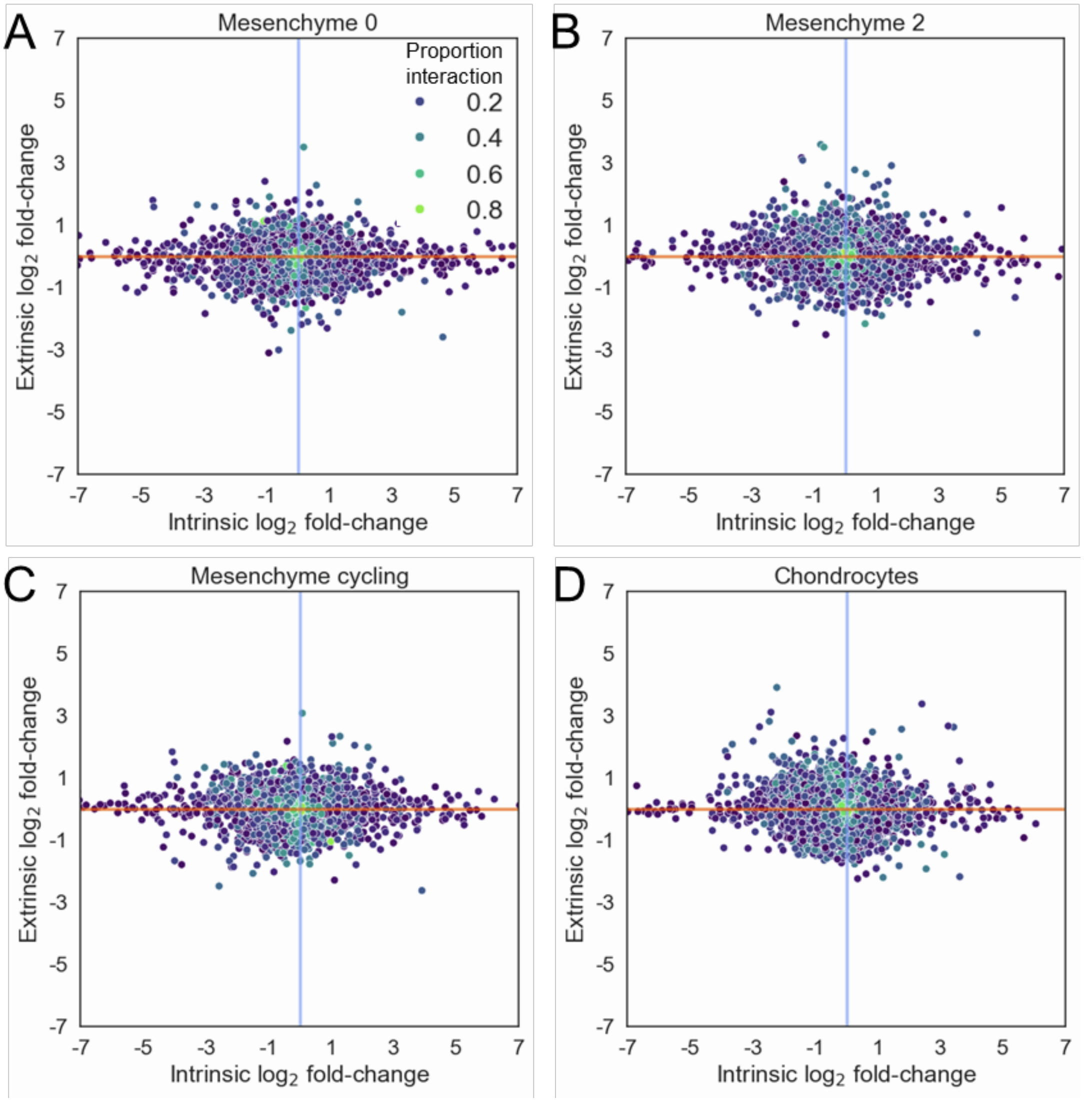

Fig. S11: Intrinsic and extrinsic divergence of connective tissue cell types, related to figure 3. Each point is a gene. Intrinsic divergence is on the y-axis and extrinsic divergence is on the x-axis. Genes are colored by their proportion interaction. **A)** Plot for mesenchyme cluster 0. **B)** Plot for mesenchyme cluster 2. **C)** Plot for cycling mesenchymal cells. **D)** Plot for chondrocytes.

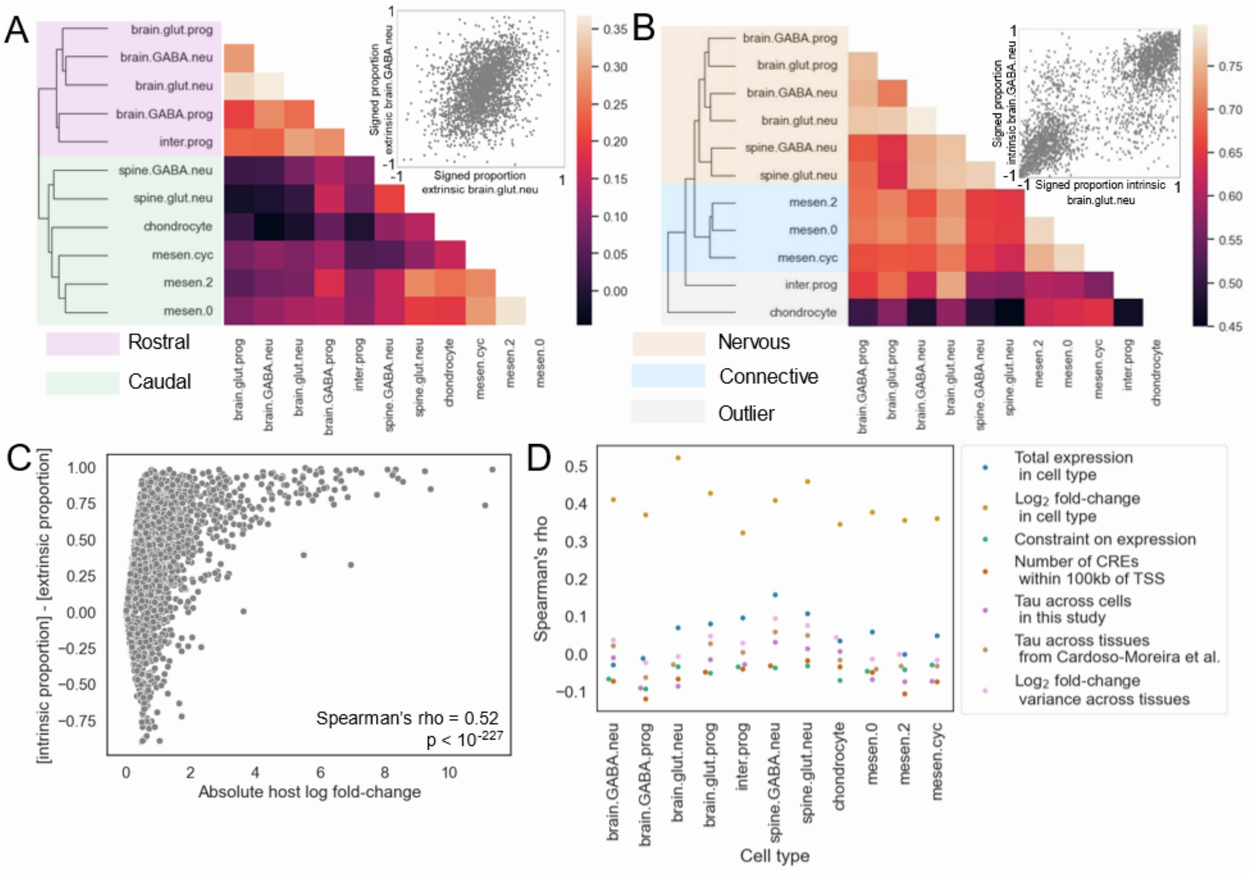

Fig. S12: Correlates of intrinsic and extrinsic divergence across cell types, related to figure 3. A) Heatmap showing Spearman correlation of signed proportion extrinsic divergence between cell types. Hierarchical clustering was performed on the Spearman rho values using the Euclidean distance metric. Cell types are shaded by their anatomical location of origin, with spinal neurons being more caudal similar to connective tissue cells. For the scatter plot in the upper right, each dot is a gene, the x-axis is the forebrain glutamatergic neuron signed proportion extrinsic divergence, and the y-axis is the forebrain GABAergic neuron signed proportion extrinsic divergence. **B)** The same as in (A) but showing the signed proportion intrinsic divergence and shading cell types by whether they cluster with nervous system cell types, connective tissue cell types, or are outliers. **C)** Scatter plot showing the relationship between absolute $\log_2$ fold-change between mouse cells in a mouse-like environment and rat cells in a rat-like environment (x-axis) and the proportion extrinsic subtracted from the proportion

intrinsic (referred to as [proportion intrinsic - proportion extrinsic], y-axis) for each gene in forebrain glutamatergic neurons. **D)** Spearman correlation coefficients for different per-gene variables and [proportion intrinsic - proportion extrinsic] across cell types.

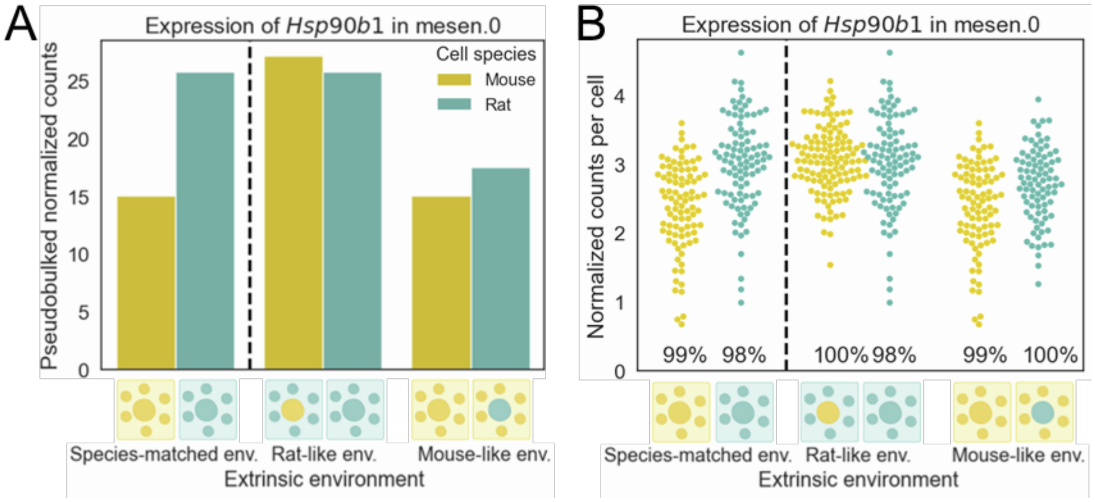

Fig. S13: Expression of *Hsp90b1*, related to figure 4. A) Expression of *Hsp90b1*, a gene involved in the ER stress response, in mesenchymal cluster 0. **B)** Per-cell expression of *Hsp90b1* in in mesenchymal cluster 0. Each swarm of points shows the normalized counts for a gene in each cell with non-zero counts for that gene. The percentage near the bottom of the plot indicates the percentage of cells with non-zero counts for that gene.

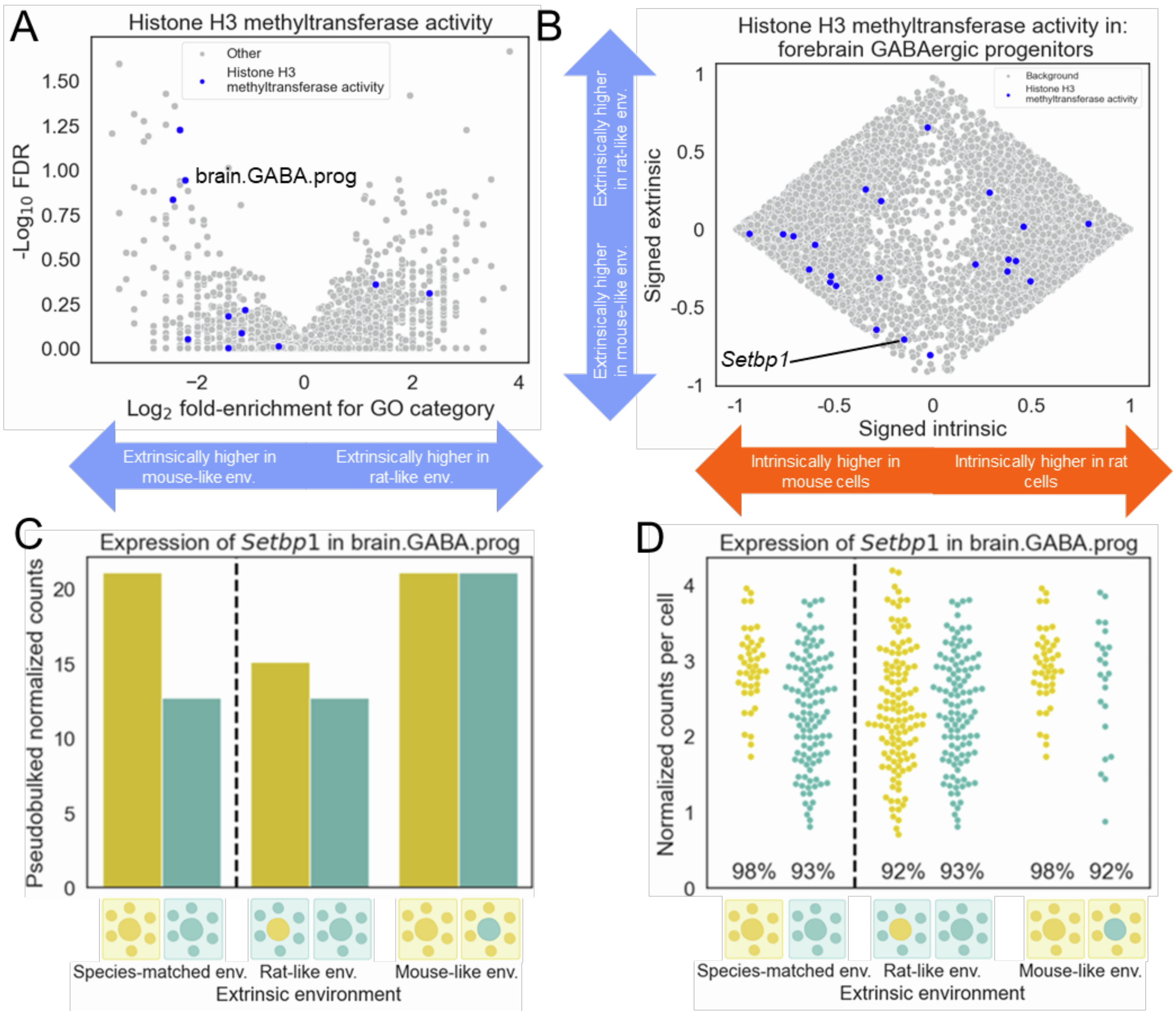

Fig. S14: Extrinsic divergence of genes encoding histone methyltransferases in central nervous system cell types, related to figure 4. A) Enrichment of genes encoding histone H3 methyltransferase for signed extrinsic divergence across cell types. Each point is a GO biological process category in a cell type and the points corresponding to the histone H3 methyltransferase activity GO category are colored blue. The x-axis shows the $\log_2$ fold-enrichment and the y-axis shows the $-\log_{10}$ false discovery rate. **B)** Scatterplot showing signed proportion intrinsic divergence (x-axis) and signed proportion extrinsic divergence (y-axis) for all genes passing our filtering criteria for forebrain GABAergic progenitors. Genes coding for histone H3 methyltransferases are shown in blue and all other genes are shown in grey. **C)**

Expression of *Setbp1*, a gene involved in histone methylation, in forebrain GABAergic progenitors. **D)** Per-cell expression *Setbp1* in forebrain GABAergic progenitors. Each swarm of points shows the normalized counts for a gene in each cell with non-zero counts for that gene. The percentage near the bottom of the plot indicates the percentage of cells with non-zero counts for that gene.

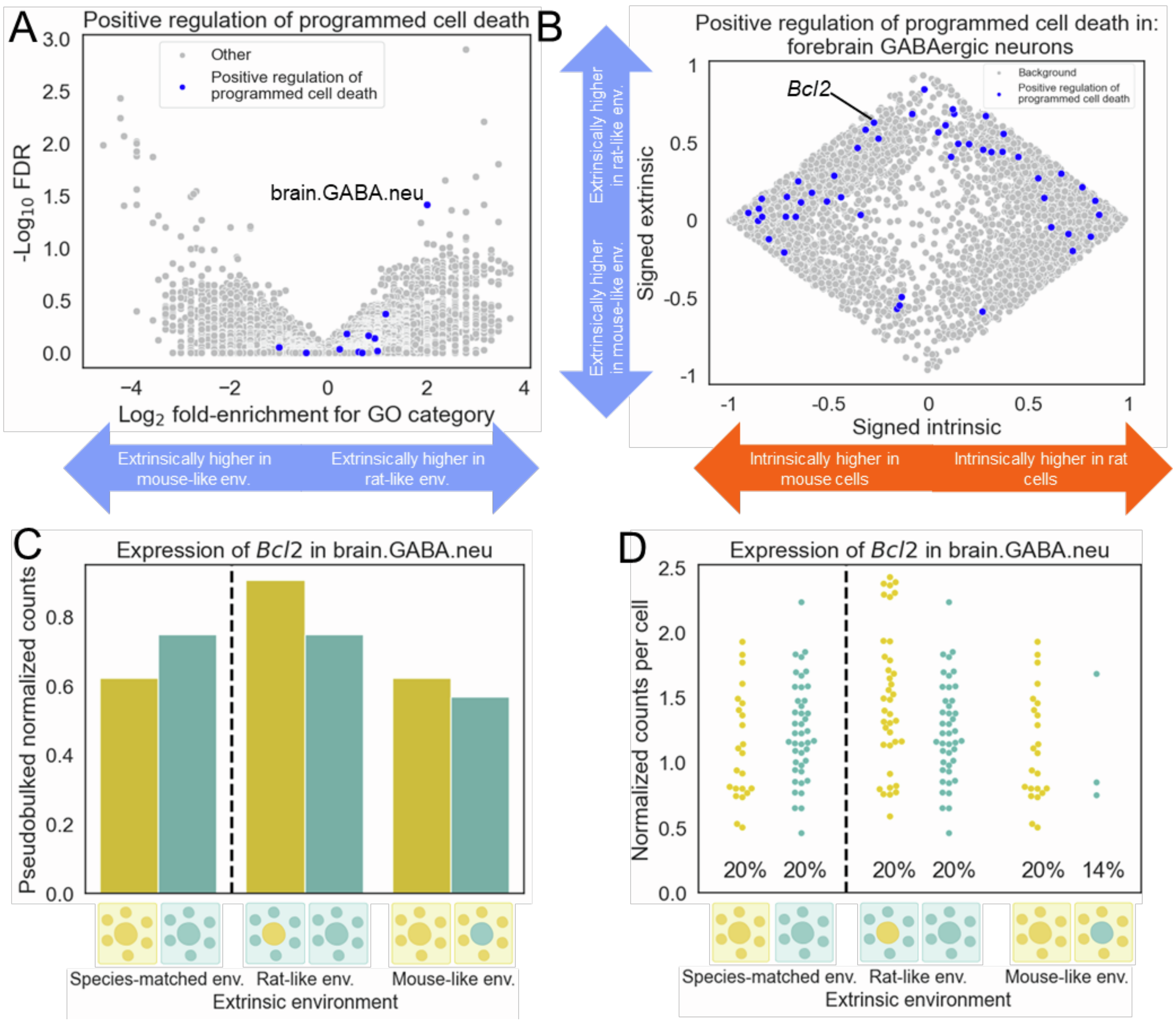

Fig. S15: Extrinsic divergence of genes involved in the positive regulation of programmed cell death in forebrain GABAergic neurons, related to figure 4. A) Enrichment of genes involved in the positive regulation of programmed cell death for signed extrinsic divergence across cell types. Each point is a GO biological process category in a cell type and the points corresponding to the positive regulation of programmed cell death GO category are colored blue. The x-axis shows the $\log_2$ fold-enrichment and the y-axis shows the $-\log_{10}$ false discovery rate. **B)** Scatterplot showing signed proportion intrinsic divergence (x-axis) and signed proportion extrinsic divergence (y-axis) for all genes passing our filtering criteria for forebrain

GABAergic neurons. Genes involved in the positive regulation of programmed cell death are shown in blue and all other genes are shown in grey. **C)** Expression of *Bcl2*, a gene involved in the regulation of programmed cell death, in forebrain GABAergic neurons. **D)** Per-cell expression *Bcl2* in forebrain GABAergic neurons. Each swarm of points shows the normalized counts for a gene in each cell with non-zero counts for that gene. The percentage near the bottom of the plot indicates the percentage of cells with non-zero counts for that gene.

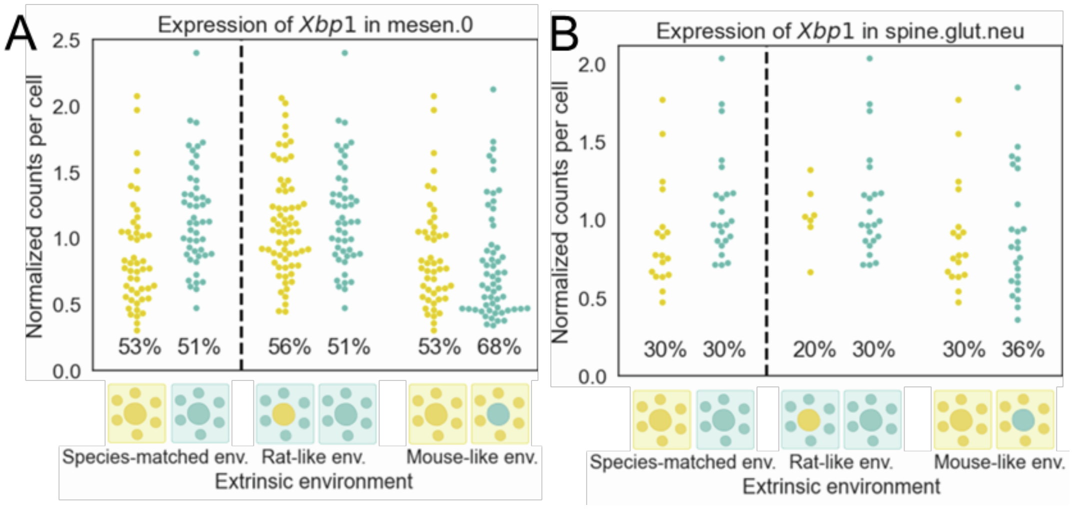

Fig. S16: Per-cell expression of *Xbp1*, related to figure 4. Each swarm of points shows the normalized counts for a gene in each cell with non-zero counts for that gene. The percentage near the bottom of the plot indicates the percentage of cells with non-zero counts for that gene. **A)** Per-cell expression *Xbp1* in mesenchymal cluster 0. **B)** Per-cell expression of *Xbp1* in spinal glutamatergic neurons.

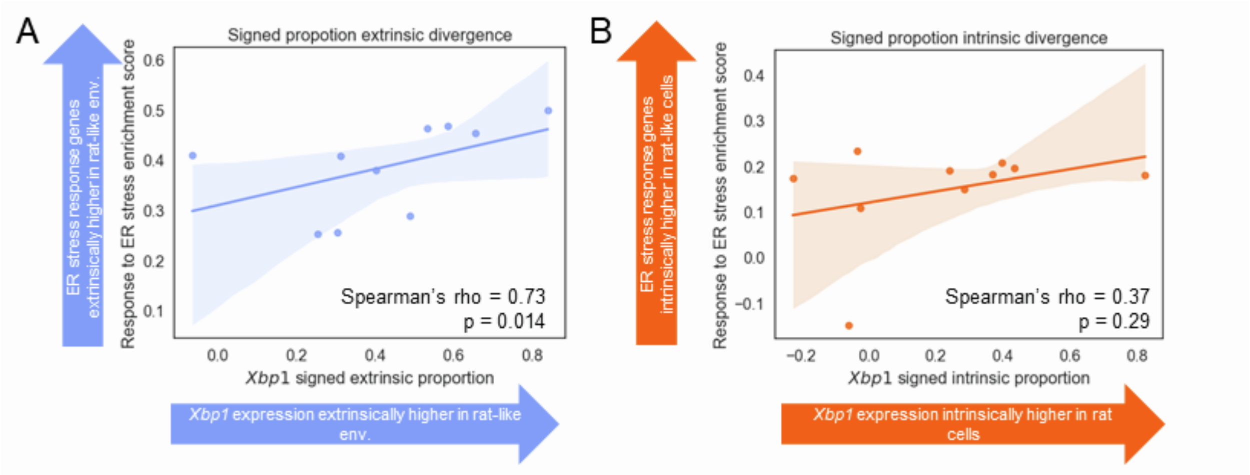

Fig. S17: Relationship between divergence in *Xbp1* expression and divergence in ER stress response gene expression, related to figure 4. A) Plot showing the relationship between signed proportion extrinsic divergence for *Xbp1* (x-axis) and the GSEA preranked enrichment score for the Response to ER stress GO category and signed proportion extrinsic divergence (y-axis). Each point is a cell type and cell types with extrinsically driven increased expression of *Xbp1* in a rat-like environment have larger values on the x-axis. Cell types with extrinsically driven increased expression of ER stress response genes in a rat-like environment have larger values on the y-axis. The line and shaded region represent the best fit and 95% confidence interval of a linear model fit to the data. **B)** Plot showing the relationship between signed proportion intrinsic divergence for *Xbp1* (x-axis) and the GSEA preranked enrichment score for the Response to ER stress GO category and signed intrinsic proportion divergence (y-axis). Each point is a cell type and cell types with intrinsically driven increased expression of *Xbp1* in rat cells have larger values on the x-axis. Cell types with intrinsically driven increased expression of ER stress response genes in rat cells have larger values on the y-axis. The line and shaded region represent the best fit and 95% confidence interval of a linear model fit to the data.

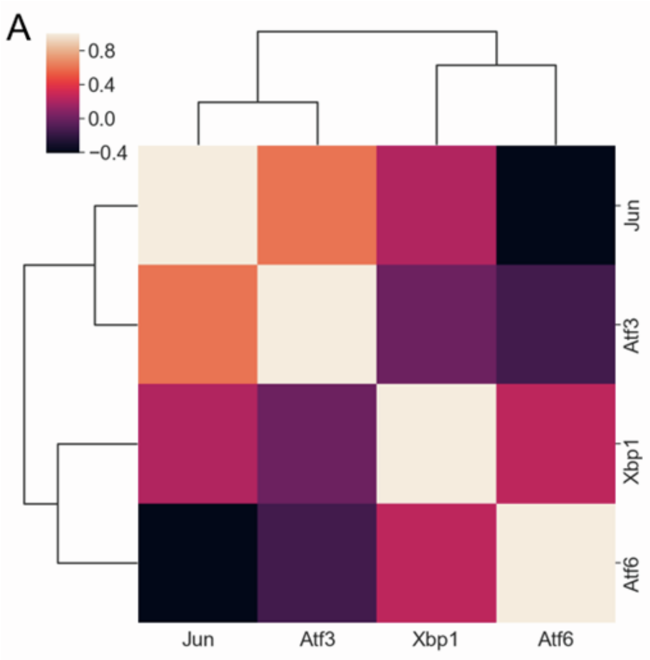

Fig. S18: Correlation between ER stress response TF signed proportion extrinsic divergence, related to figure 4. A) Heatmap showing the correlation between the signed proportion extrinsic divergence of TFs associated with the ER stress response. The genes were hierarchically clustered using the Euclidean distance metric. Only cell types in which both genes passed our filtering criteria were used to compute the correlation (see Methods).

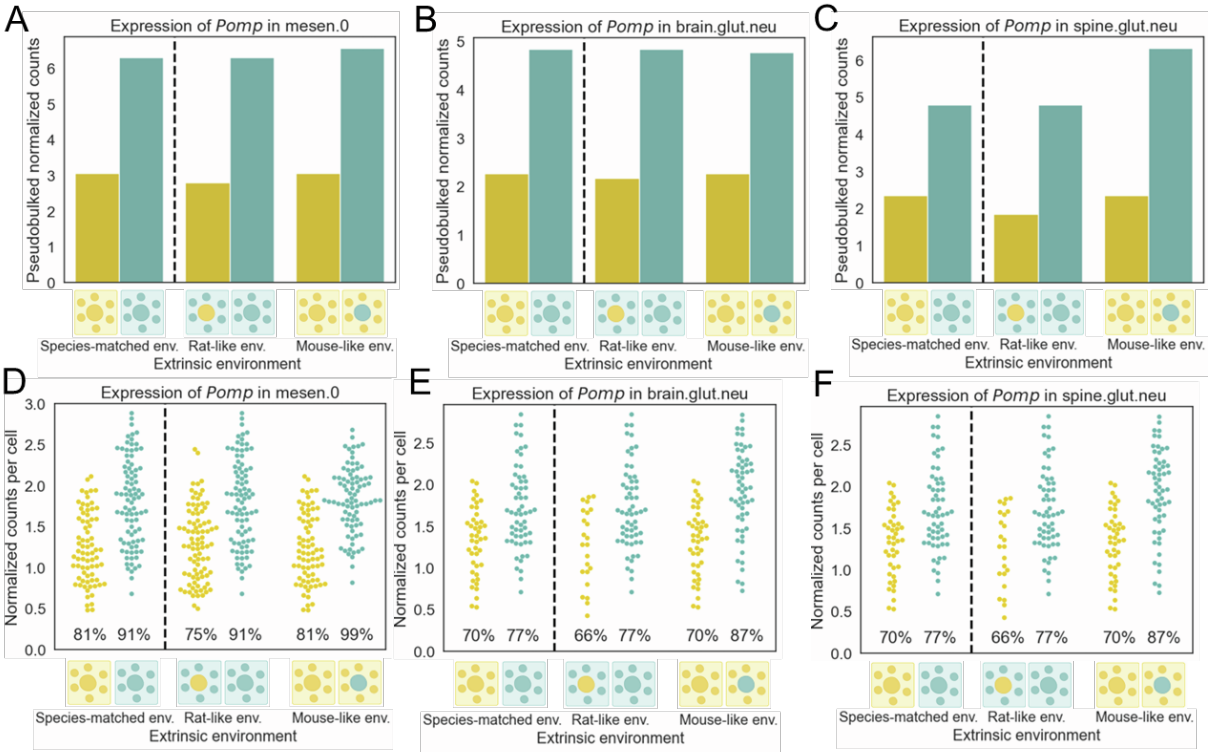

Fig. S19: Expression of *Pomp* across cell types, related to figure 5. A) Expression of the *Nfe2l1* target gene *Pomp* in mesenchymal cluster 0 cells. Expression is intrinsically higher in rat cells. **B)** Expression of *Pomp* in forebrain glutamatergic neurons. Expression is intrinsically higher in rat cells. **C)** Expression of *Pomp* in spinal glutamatergic neurons. Expression is primarily intrinsically higher in rat cells. **D)** Per-cell expression *Pomp* in mesenchymal cluster 0. Each swarm of points shows the normalized counts for a gene in each cell with non-zero counts for that gene. The percentage near the bottom of the plot indicates the percentage of cells with non-zero counts for that gene. **E)** Per-cell expression of *Pomp* in forebrain glutamatergic neurons. **F)** Per-cell expression of *Pomp* in spinal glutamatergic neurons.

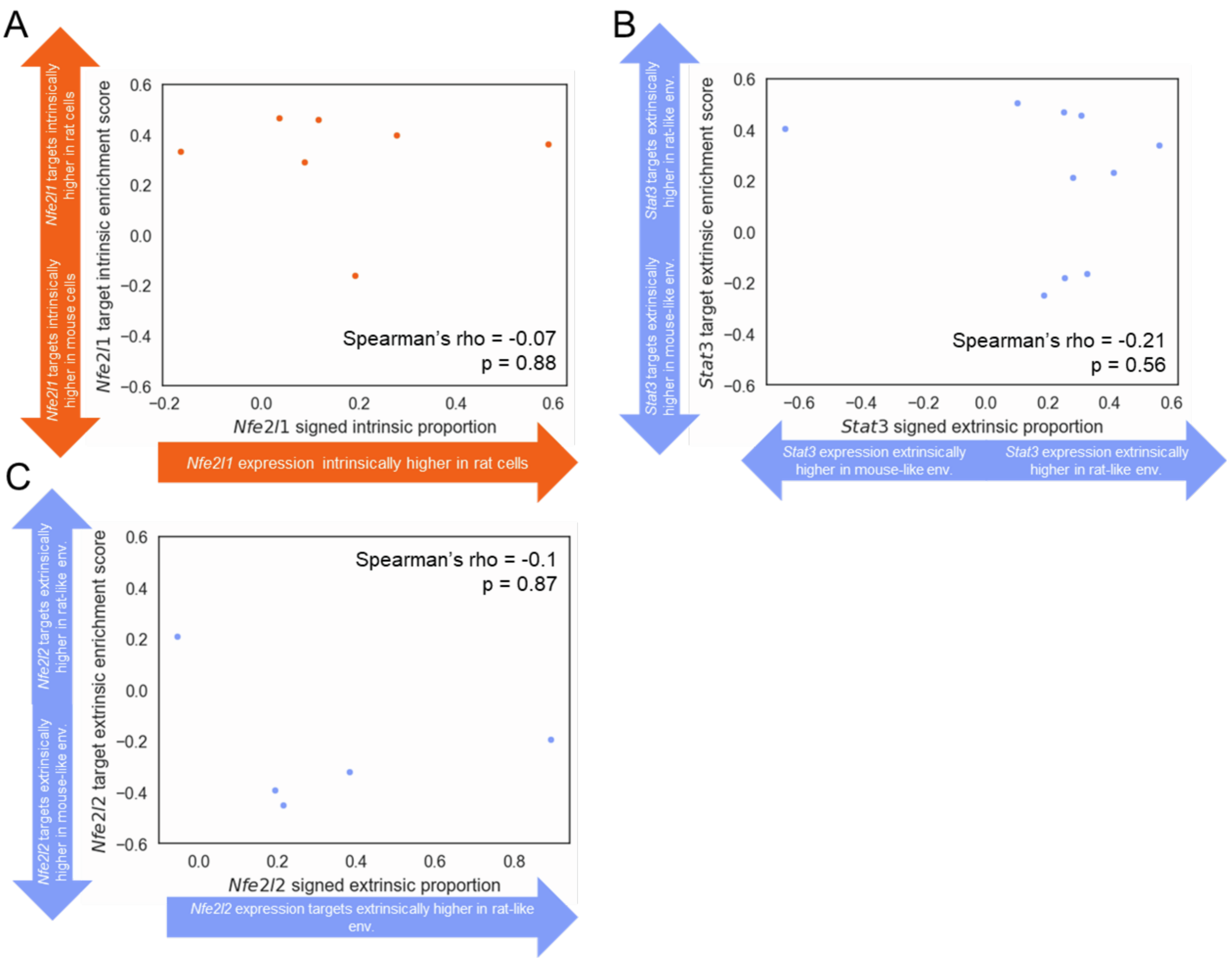

Fig. S20: Analysis of TFs regulating proteasomal subunit expression and their target genes, related to figure 5. A) Scatter plot showing the relationship between signed intrinsic proportion divergence for *Nfe2l1* (x-axis) and the enrichment of its target genes for signed intrinsic proportion divergence (y-axis). Each point is a cell type and cell types with intrinsically driven increased expression of *Nfe2l1* in rat cells have larger values on the x-axis. Cell types with intrinsically driven increased expression of *Nfe2l1* target genes in rat cells have larger values on the y-axis. **B)** Correlation between signed extrinsic proportion divergence for *Stat3* (x-axis) and the enrichment of its target genes for signed extrinsic proportion divergence (y-axis). Each point is a cell type and cell types with extrinsically driven increased expression of *Stat3* in a rat-like environment have larger values on the x-axis and cell types with extrinsically driven

increased expression of *Stat3* in a mouse-like environment have smaller values on the x-axis. Cell types with extrinsically driven increased expression of *Stat3* target genes in a rat-like environment have larger values on the y-axis whereas cell types with extrinsically driven increased expression of *Stat3* target genes in a mouse-like environment have smaller values on the y-axis. **C)** Correlation between signed extrinsic proportion divergence for *Nfe2l2* (x-axis) and the enrichment of its target genes for signed extrinsic proportion divergence (y-axis). Each point is a cell type and cell types with extrinsically driven increased expression of *Nfe2l2* in a rat-like environment have larger values on the x-axis and cell types with extrinsically driven increased expression of *Nfe2l2* in a mouse-like environment have smaller values on the x-axis. Cell types with extrinsically driven increased expression of *Nfe2l2* target genes in a rat-like environment have larger values on the y-axis whereas cell types with extrinsically driven increased expression of *Nfe2l2* target genes in a mouse-like environment have smaller values on the y-axis.

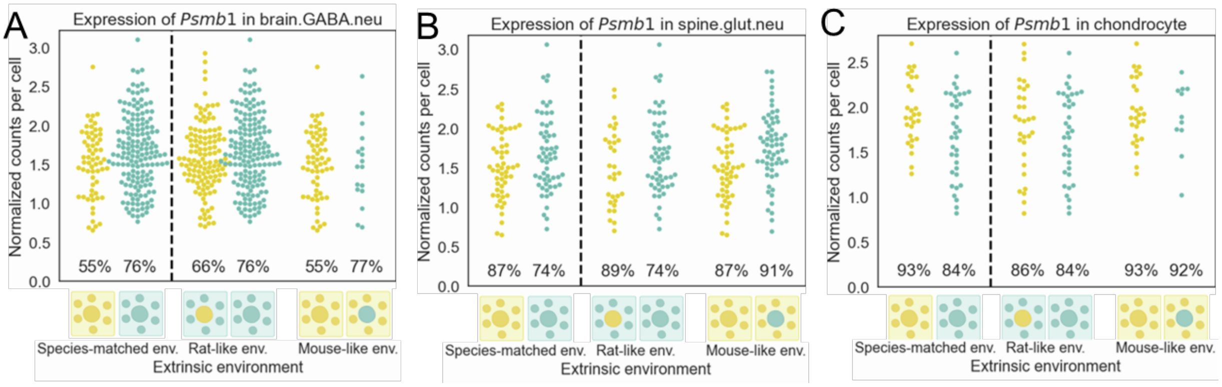

Fig. S21: Per-cell expression of *Psmb1* across cell types, related to figure 5. Each swarm of points shows the normalized counts for a gene in each cell with non-zero counts for that gene. The percentage near the bottom of the plot indicates the percentage of cells with non-zero counts for that gene. **A)** Per-cell expression *Psmb1* in forebrain GABAergic neurons. **B)** Per-cell expression of *Psmb1* in spinal glutamatergic neurons. **C)** Per-cell expression of *Psmb1* in chondrocytes.

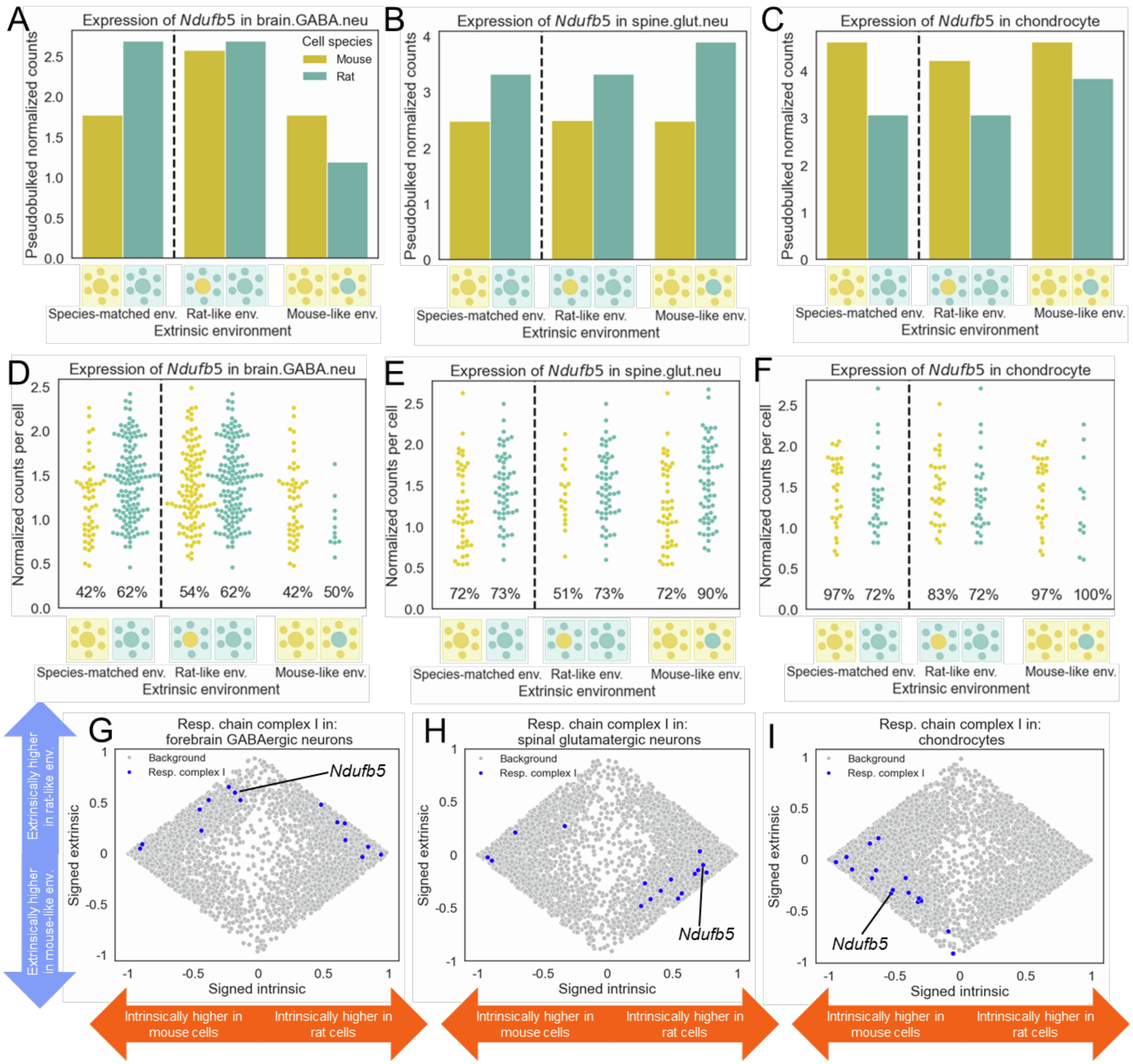

Fig. S22: Cell-type specific intrinsic and extrinsic divergence of the expression of genes encoding mitochondrial respiratory chain complex I subunits across cell types, related to figure 5. A) Expression of *Ndufb5* in forebrain GABAergic neurons. **B)** Same as in (A) but for spinal glutamatergic neurons. **C)** Same as in (A) but for chondrocytes. **D)** Per-cell expression *Ndufb5* in forebrain GABAergic neurons. Each swarm of points shows the normalized counts for a gene in each cell with non-zero counts for that gene. The percentage near the bottom of the plot indicates the percentage of cells with non-zero counts for that gene. **E)** Per-cell expression

of *Ndufb5* in spinal glutamatergic neurons. **F)** Per-cell expression of *Ndufb5* in chondrocytes. **G)** Scatterplot showing signed proportion intrinsic divergence (x-axis) and signed proportion extrinsic divergence (y-axis) for all genes passing our filtering criteria for forebrain GABAergic neurons. Genes coding for mitochondrial respiratory chain complex I subunits are shown in blue and all other genes are shown in grey. Expression of genes coding for mitochondrial respiratory chain complex I subunits is generally extrinsically higher in a rat-like environment. **H)** Same as in (G) but for spinal glutamatergic neurons. Expression of genes coding for mitochondrial respiratory chain complex I subunits is generally intrinsically higher in rat cells but extrinsically higher in a mouse-like environment. **I)** Same as in (G) but for chondrocytes. Expression of genes coding for mitochondrial respiratory chain complex I subunits is generally intrinsically higher in mouse cells and extrinsically higher in a mouse-like environment.

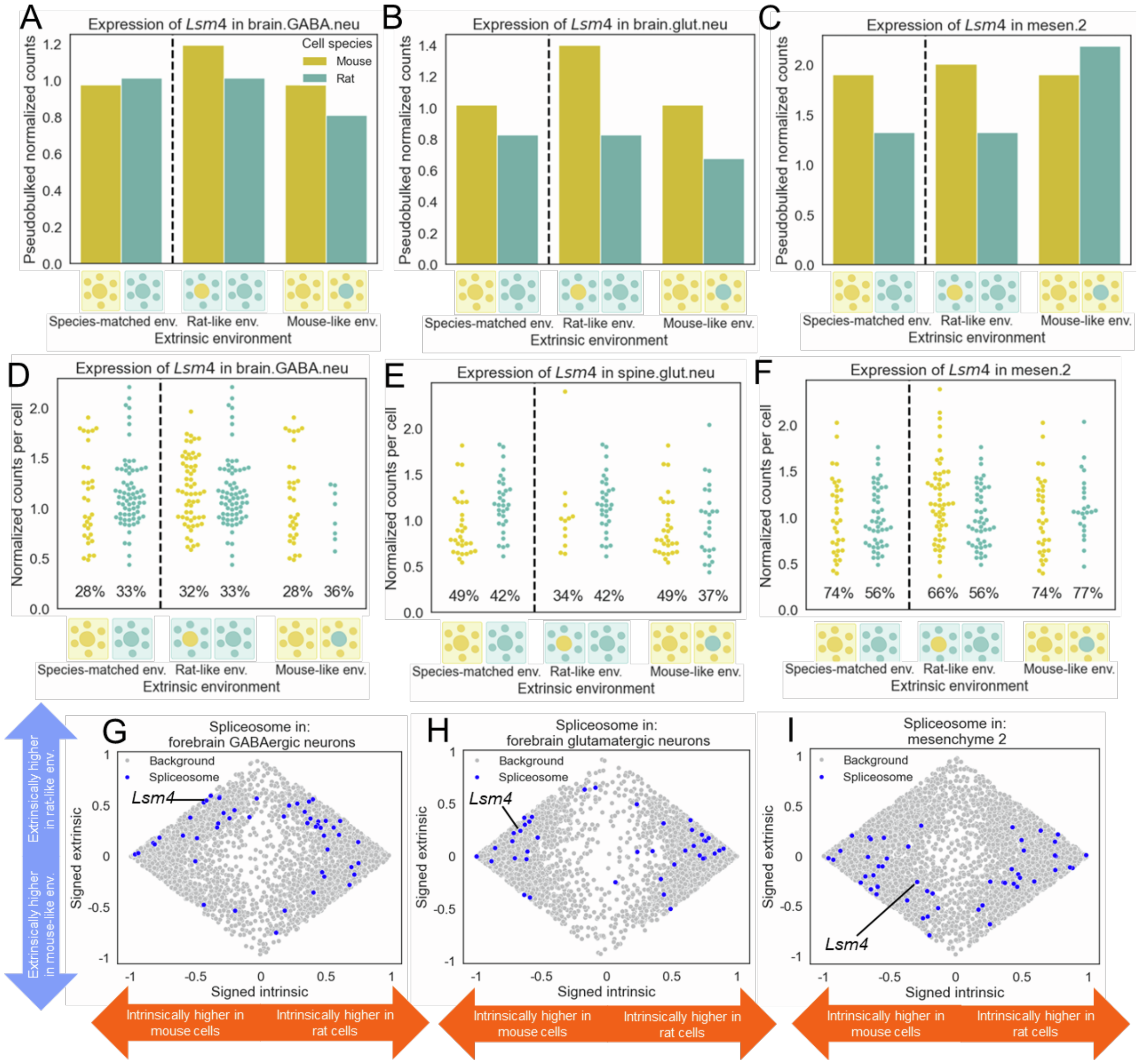

Fig. S23: Cell-type specific intrinsic and extrinsic divergence of the expression of genes encoding spliceosomal subunits across cell types, related to figure 5. A) Expression of *Lsm4* in forebrain GABAergic neurons. **B)** Same as in (A) but for spinal glutamatergic neurons. **C)** Same as in (A) but for mesenchymal cluster 2 cells. **D)** Per-cell expression *Lsm4* in forebrain GABAergic neurons. Each swarm of points shows the normalized counts for a gene in each cell with non-zero counts for that gene. The percentage near the bottom of the plot indicates the percentage of cells with non-zero counts for that gene. **E)** Per-cell expression of *Lsm4* in spinal glutamatergic neurons. **F)** Per-cell expression of *Lsm4* in mesenchymal cluster 2 cells. **G)**

Scatterplot showing signed proportion intrinsic divergence (x-axis) and signed proportion extrinsic divergence (y-axis) for all genes passing our filtering criteria for forebrain GABAergic neurons. Genes coding for spliceosomal subunits are shown in blue and all other genes are shown in grey. Expression of genes coding for spliceosomal subunits is generally extrinsically higher in a rat-like environment. **H)** Same as in (G) but for forebrain glutamatergic neurons. Expression of genes coding for spliceosomal subunits is generally extrinsically higher in a mouse-like environment and intrinsically higher in rat cells. **I)** Same as in (G) but for mesenchymal cluster 2 cells. Expression of genes coding for spliceosomal subunits is generally extrinsically higher in a mouse-like environment and intrinsically higher in mouse cells.

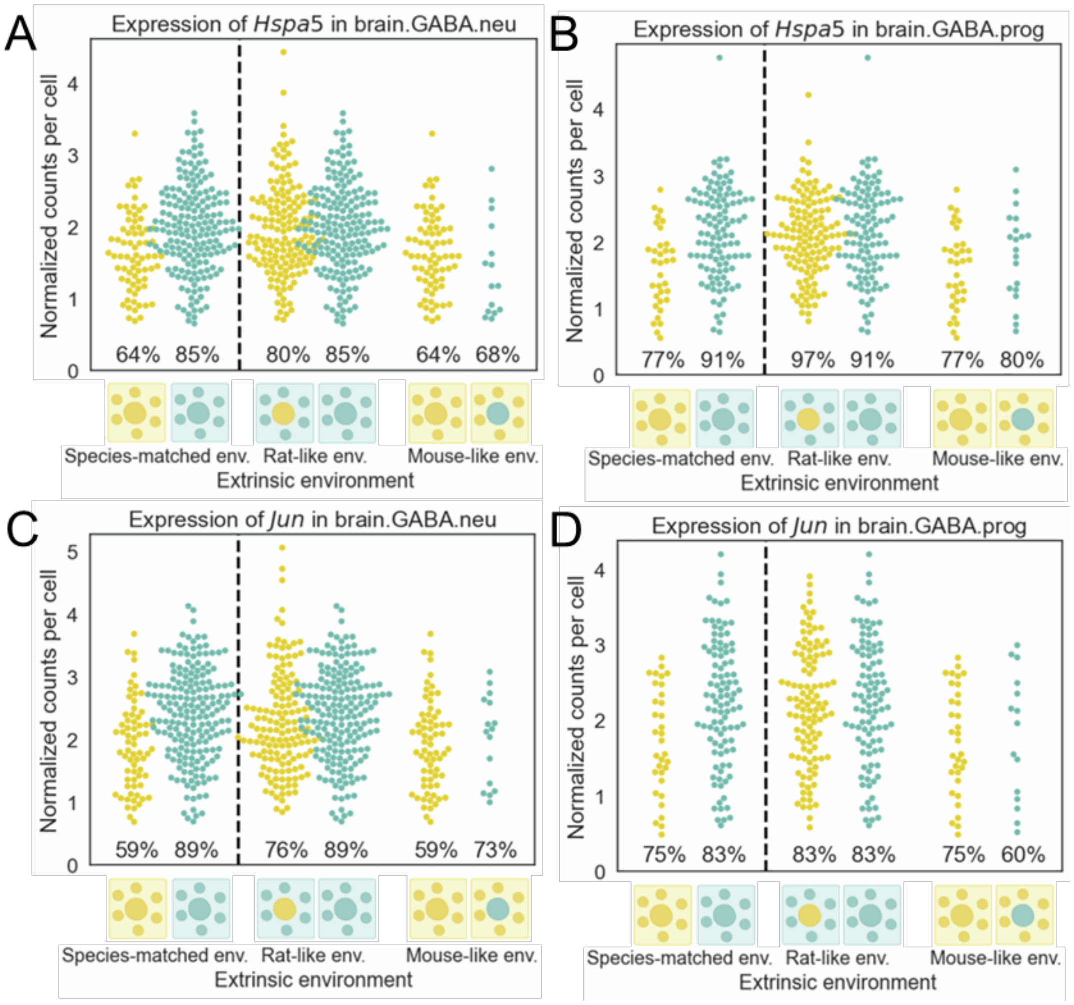

Fig. S24: Per-cell expression of *Jun* and *Hspa5* in forebrain GABAergic neurons and progenitors, related to figure 6. Each swarm of points shows the normalized counts for a gene in each cell with non-zero counts for that gene. The percentage near the bottom of the plot indicates the percentage of cells with non-zero counts for that gene. **A)** Per-cell expression of *Hspa5* in forebrain GABAergic neurons. **B)** Per-cell expression of *Hspa5* in forebrain GABAergic progenitors. **C)** Per-cell expression of *Jun* in forebrain GABAergic neurons. **D)** Per-cell expression of *Jun* in forebrain GABAergic progenitors.

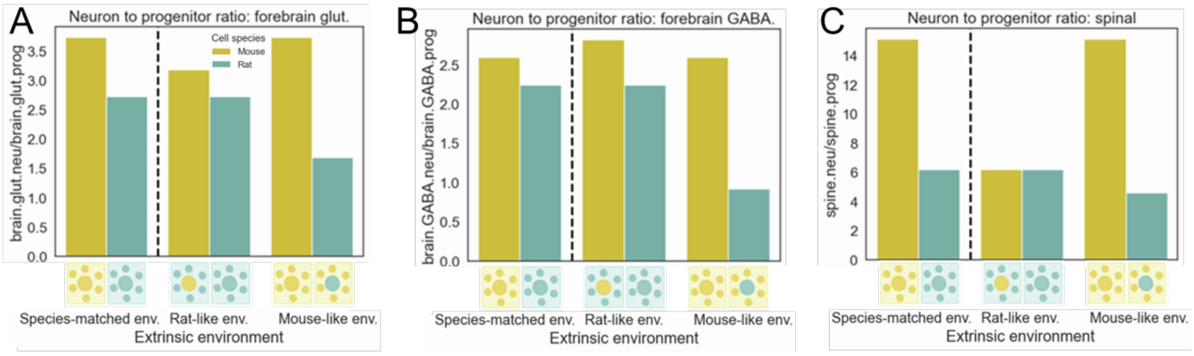

Fig. S25: Neuron-to-progenitor ratio in different neurogenic niches, related to figure 6. A) Plot showing the neuron-to-progenitor ratio for forebrain glutamatergic neurogenesis across the four different species-environment combinations. **B)** Same as in (A) but for forebrain GABAergic neurogenesis. **C)** Same as in (A) but for combined GABAergic and glutamatergic spinal neurogenesis. The two lineages were combined as we were unable to distinguish GABAergic and glutamatergic progenitors.

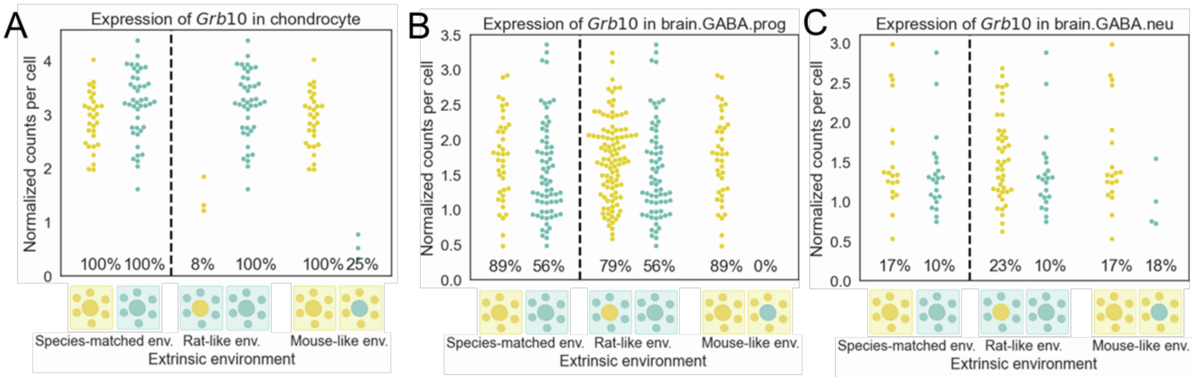

Fig. S26: Per-cell expression of *Grb10* across cell types, related to figure 7. Each swarm of points shows the normalized counts for a gene in each cell with non-zero counts for that gene. The percentage near the bottom of the plot indicates the percentage of cells with non-zero counts for that gene. **A)** Per-cell expression of *Grb10* in chondrocytes. **B)** Per-cell expression of *Grb10* in forebrain GABAergic progenitors. **C)** Per-cell expression of *Grb10* in forebrain GABAergic neurons.

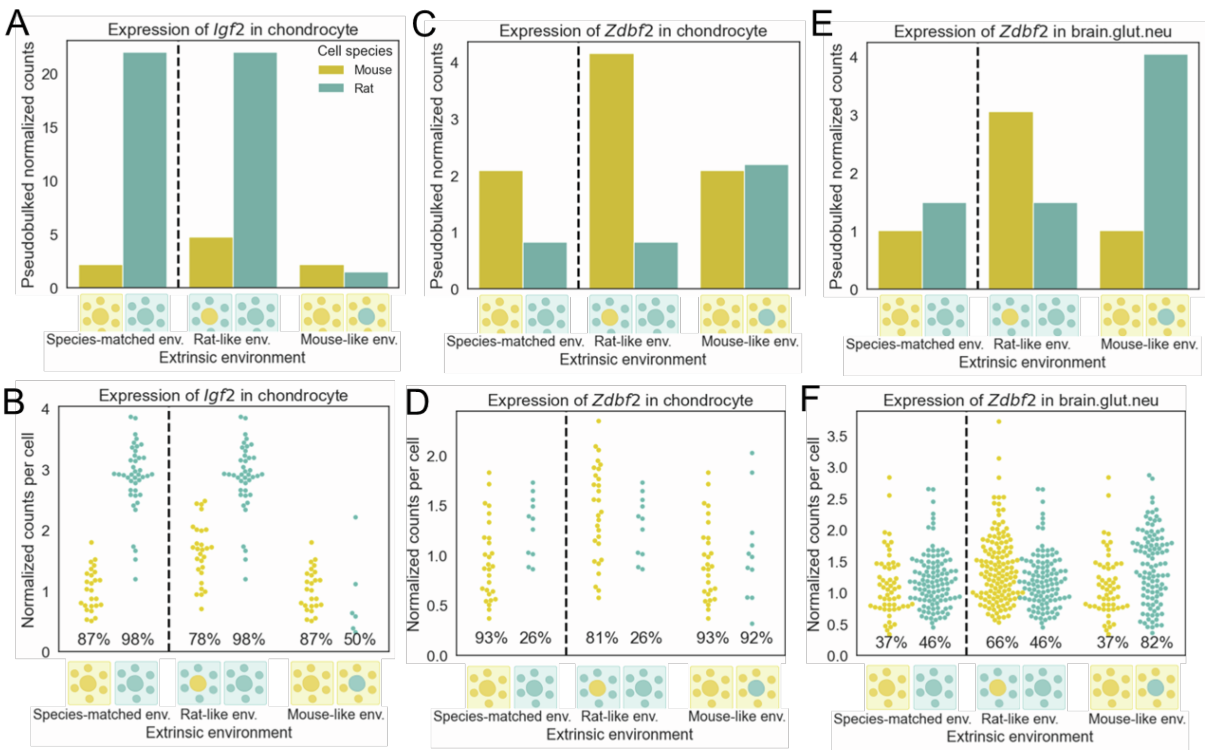

Fig. S27: Expression of selected imprinted genes, related to figure 7. A) Expression of *Igf2* across the four species-environment combinations in chondrocytes. Expression is very high for rat cells in a rat-like environment but is very low for rat cells in a mouse-like environment. **B)** Per-cell expression *Igf2* in chondrocytes. Each swarm of points shows the normalized counts for a gene in each cell with non-zero counts for that gene. The percentage near the bottom of the plot indicates the percentage of cells with non-zero counts for that gene. **C)** Expression of the imprinted gene *Zdbf2* across the four species-environment combinations in chondrocytes. Expression is higher in species-mismatched environments in cells from both species. **D)** Per-cell expression of *Zdbf2* in chondrocytes. **E)** Expression of the imprinted gene *Zdbf2* in forebrain glutamatergic neurons across the four species-environment combinations in forebrain glutamatergic neurons. Expression is higher in species-mismatched environments in cells from both species. **F)** Per-cell expression of *Zdbf2* in forebrain glutamatergic neurons.

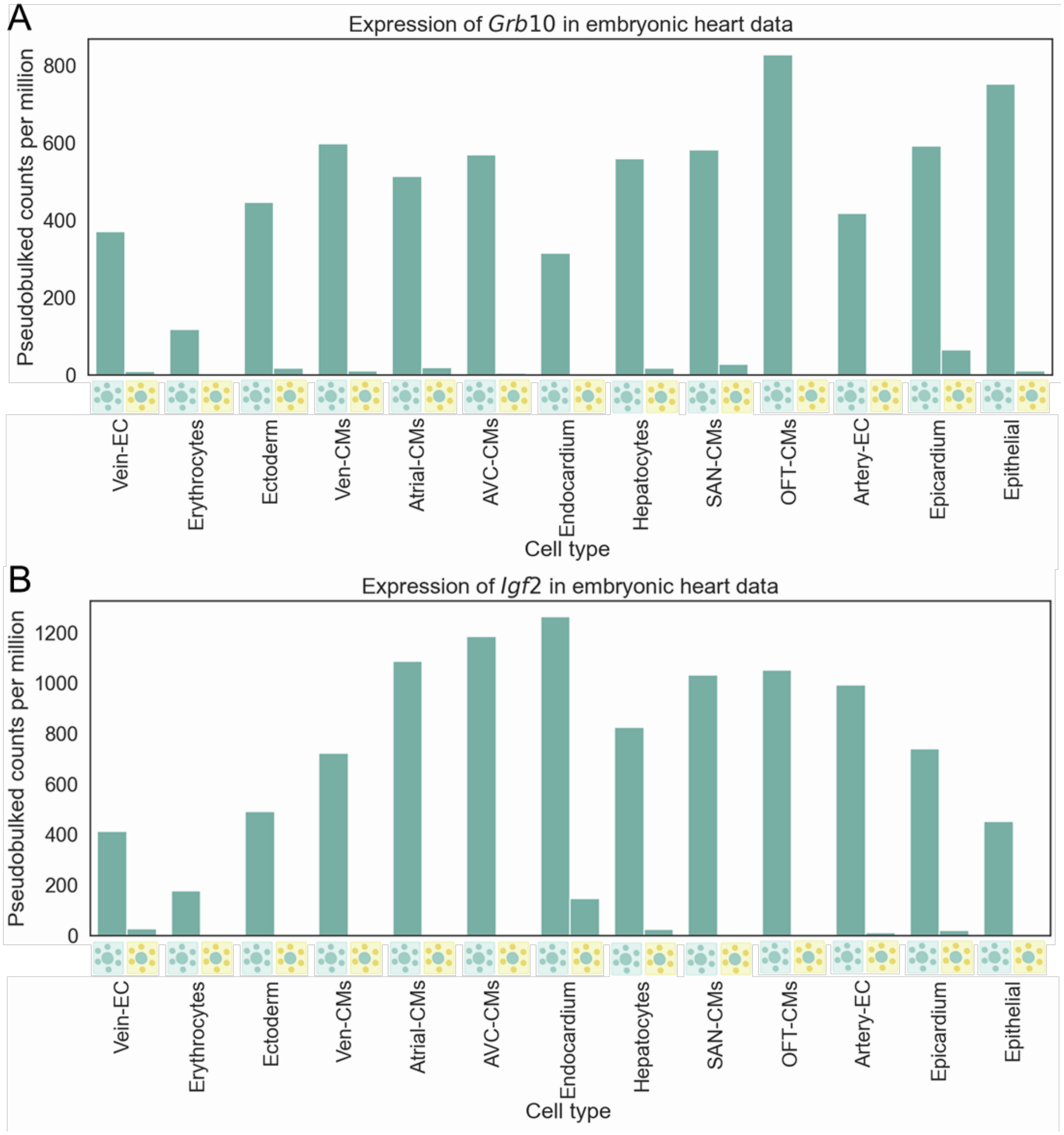

Fig. S28: Expression of imprinted genes *Grb10* and *Igf2* across cell types from the heart dataset, related to figure 7. For each cell type, expression in rat cells in a rat-like environment is shown on the left and expression in rat cells in a mouse-like environment is shown on the right. **A)** Expression of *Grb10* across cell types. Expression is higher in a species-matched

environment. **B)** Expression of *Igf2* across cell types. Expression is higher in a species-matched environment.

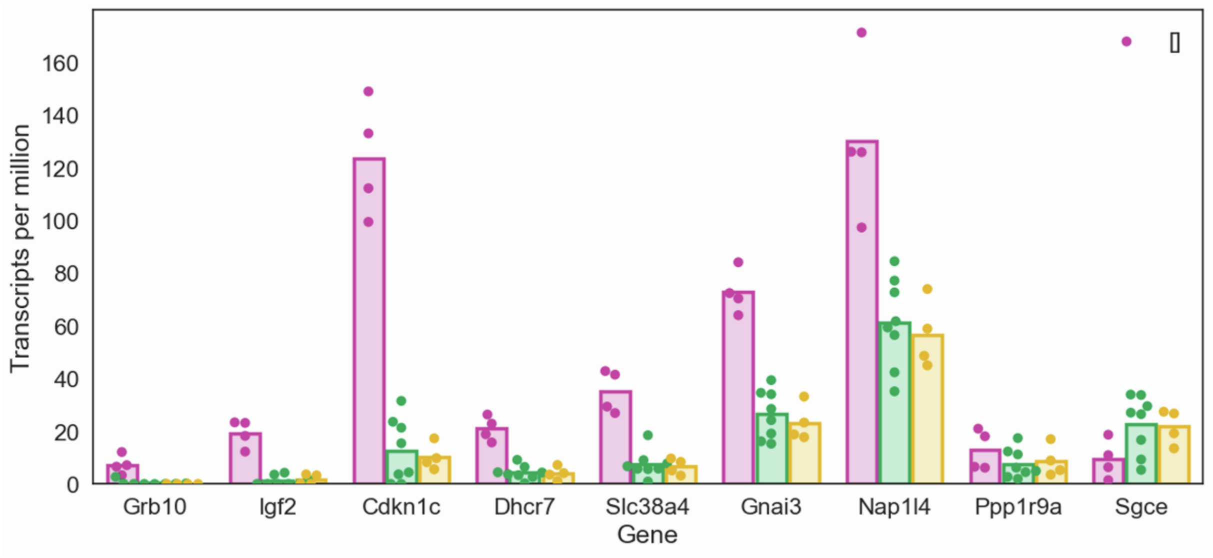

Fig. S29: Disrupted expression of imprinted genes in adult mouse parathyroid cells in species-mismatched environments, related to figure 7. A) Expression levels of mis-expressed imprinted genes for samples from species-mismatched environments, species-matched environments, and wildtype mice. Expression is higher in species-mismatched environments, but very similar between donor mouse cells in species-matched environments and wildtype mice. All imprinted genes with absolute difference between expression for species-mismatched and species-matched environment samples greater than 1 were plotted.

	Cell line	Embryo strain	Number of cells injected into embryo	stage (Embryonic)	No. of neonates	No. of chimeras
					(%)	(%)
mouse ESCs	CD1 (SUN106.2)	Wistar rat	5-7	13.5	23 (37)	7 (30)
	C57BL/6 (SGE2)	Wistar rat	5-7		23 (44)	16 (70)
Rat ESCs	Wistar (rat ESC No.3)	CD1 mouse	5-7	15.25	19(19)	10(53)
		C57BL/6 mouse	5-7		7(7)	1(14)

Table S4, related to STAR Methods: Results of all embryo injections performed.

			Donor chimerism analyzed by Flow cytometry as a percentage, (scRNA-seq library)		
Chimera	Donor cell	Host embryo	Chimera No.	Connective tissue	Forebrain
Mouse to Rat	SUN106.2	Wistar	1	23.1	1.84 (MR1)
			2	4.76 (MR3)	0
			3	0.95	0.03
			4	22.7	1.02 (MR2)
			5	15.4	0.64 (MR3)
			6	4.29 (MR2)	0
			7	3.52 (MR1)	0.089
	SGE2	Wistar	1	N/A	2.25
			2	2.22	11.5
			3	1.47	4.1
			4	0.31	3.48
			5	1.76	8.56
			6	0.15	1.86
			7	0.53	10.4
			8	3.92	21
			9	2.09	7.72
			10	0.078	1.56
			11	0.27	2.26
			12	0.45	4.33
			13	0.11	0.14
			14	0.88	0.56
			15	0.073	1.91
			16	0.6	8.81
Rat to Mouse	rat ESC No.3	C57BL/6	1	12.9	24.8
	rat ESC No.3	CD1	1	9.69	19.2
			2	5.59 (RM1)	3.14
			3	12.2	14
			4	4.52 (RM2)	9.22
			5	7.6 (RM3)	5.59
			6	2.27	1.81(RM2)
			7	4	3.23 (RM3)
			8	5.15	6.39 (RM1)
			9	8.75	15.9

Table S5, related to STAR Methods: Donor percentages for all chimeras analyzed with flow cytometry. The abbreviation in the parentheses indicate the library to which the tissue contributed.
