# Supplementary material for: Disentangling cell-intrinsic and cell-extrinsic factors underlying evolution
Source: Cell Genom. 2025 May 29;5(8):100891. doi: 10.1016/j.xgen.2025.100891 (PMC12366658; doi:10.1016/j.xgen.2025.100891)
Supplement: Document S2. Article plus supplemental information [file mmc5.pdf]

Cell Genomics

Article

Disentangling cell-intrinsic and cell-extrinsic factors underlying evolution

Graphical abstract

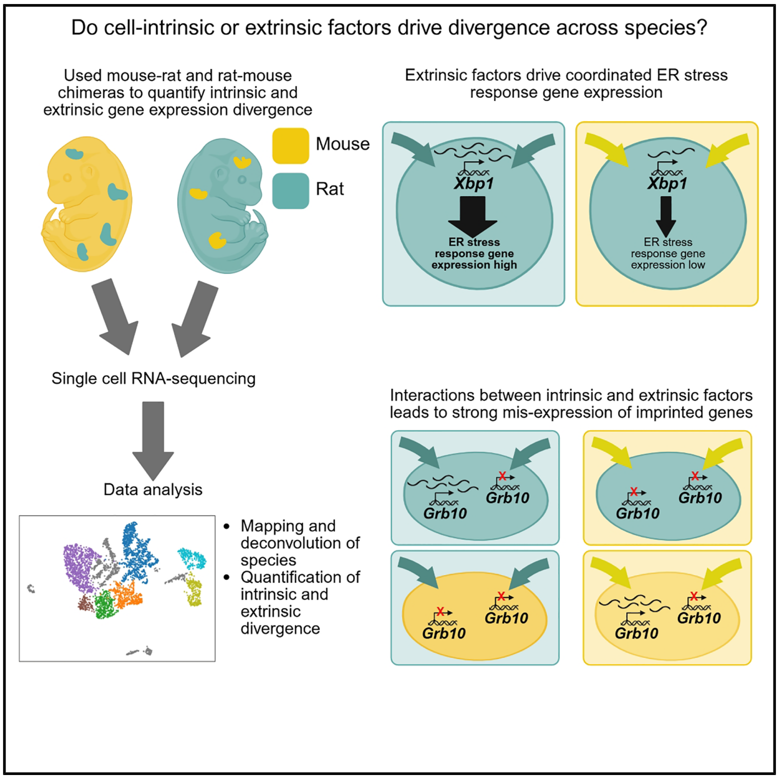

Authors

Alexander L. Starr, Toshiya Nishimura, Kyomi J. Igarashi, Chihiro Funamoto, Hiromitsu Nakauchi, Hunter B. Fraser

Correspondence

hbfraser@stanford.edu

In brief

Cellular function is shaped by factors both intrinsic and extrinsic to every cell. Starr and Nishimura et al. introduce a mathematical framework to decompose trait divergence into cell-intrinsic/extrinsic components. They find that most gene expression divergence between mouse and rat is cell intrinsic and that imprinted genes are severely misexpressed in interspecies chimeras.

Highlights

- Cellular function is shaped by factors both intrinsic and extrinsic to every cell
- We invent a framework to decompose trait divergence into intrinsic/extrinsic components
- Rat-mouse chimeras show that most gene expression divergence is cell intrinsic
- Misexpression of imprinted genes suggests intrinsic/extrinsic incompatibilities

Starr et al., 2025, Cell Genomics *5*, 100891
August 13, 2025 © 2025 The Authors. Published by Elsevier Inc.
https://doi.org/10.1016/j.xgen.2025.100891

CellPress

Cell Genomics

CellPress
OPEN ACCESS

Article

Disentangling cell-intrinsic and cell-extrinsic factors underlying evolution

Alexander L. Starr,[1,6] Toshiya Nishimura,[2,3,6,7] Kyomi J. Igarashi,[4] Chihiro Funamoto,[3] Hiromitsu Nakauchi,[2,5] and Hunter B. Fraser[1,8,*]

[1]Department of Biology, Stanford University, Stanford, CA 94305, USA

[2]Institute for Stem Cell Biology and Regenerative Medicine, Department of Genetics, Stanford University School of Medicine, Stanford, CA 94305, USA

[3]Division of Stem Cell and Organoid Medicine, Department of Genome Biology, Graduate School of Medicine, Osaka University, Osaka 565-0871, Japan

[4]Department of Genetics, Stanford University School of Medicine, Stanford, CA 94305, USA

[5]Division of Stem Cell Therapy, Distinguished Professor Unit, The Institute of Medical Science, The University of Tokyo, Minato-ku, Tokyo 108-8639, Japan

[6]These authors contributed equally

[7]Present address: WPI Premium Research Institute for Human Metaverse Medicine (WPI-PRIMe), Osaka University, Osaka 565-0871, Japan

[8]Lead contact

*Correspondence: hbfraser@stanford.edu

https://doi.org/10.1016/j.xgen.2025.100891

SUMMARY

A long-standing question in biology is the extent to which cells function autonomously as opposed to requiring interactions with other cells or environmental factors. Here, we develop a framework to use interspecies chimeras to precisely decompose evolutionary divergence in any cellular trait into cell-intrinsic and cell-extrinsic components. Applying this framework to thousands of gene expression levels in reciprocal rat-mouse chimeras, we found that most divergence is cell intrinsic, though extrinsic factors also play an integral role. For example, cell-extrinsic regulation of a transcription factor can propagate to its target genes, leading to cell-type-specific extrinsic regulation of both their mRNA and their protein levels. We also show that imprinted genes are dramatically misexpressed in chimeras, suggesting a mismatch between rapidly evolving intrinsic and extrinsic imprinting mechanisms. Overall, our conceptual framework opens up new avenues to investigate the mechanistic basis of the evolution, development, and regulation of myriad cellular traits in any multicellular organism.

INTRODUCTION

Among the most enduring questions in biology is how cells execute their diverse array of functions, including division, differentiation, homeostasis, and cell death. A key component of this question is to what degree cells follow an intrinsically predetermined fate as opposed to responding to dynamic extracellular cues. Although these cellular behaviors are ultimately programmed by the genome, that genome is shared by cells present throughout a multicellular organism, and it remains largely unknown to what degree any given cell's traits are determined by the genome located within that cell vs. by the multitude of copies in the rest of the individual.

Although this distinction is important for understanding almost every area of biology, it has been most thoroughly investigated in the context of development. For example, in 1885, a pioneer of this nascent field emphasized that its central goal was distinguishing to what extent development proceeds via self-differentiation (now known as cell-autonomous or cell-intrinsic differentiation) as opposed to dependent differentiation (now known as non-cell-autonomous or cell-extrinsic differentiation).[1,2] Since then, many seminal discoveries have been made by studying this dichotomy.[3–9] Interspecies chimeras—formed by grafting donor cells from one species into the host embryo of another—have fueled many of these key insights.[3–7] In general, a process is said to be cell intrinsic if the cells responsible for executing that process behave in a similar way regardless of the genetic background of surrounding cells. For example, quail cranial neural crest cells retain their own spatial and temporal programs for gene expression when they are transplanted into a slower-developing duck embryo.[6,10,11] On the other hand, a process is cell extrinsic when it depends on the genotype of other cells; for instance, duck epidermal cells in the same quail-duck chimeras are extrinsically induced by the transplanted quail neural crest to alter their gene expression to make quail-like feathers.[6,10,11]

The interplay between cell-intrinsic and cell-extrinsic factors not only is fundamental to development but also has significant implications for every aspect of cellular function. For example, integrating intrinsic and extrinsic dynamics is critical for

© 2025 The Authors. Published by Elsevier Inc.
This is an open access article under the CC BY license (http://creativecommons.org/licenses/by/4.0/).

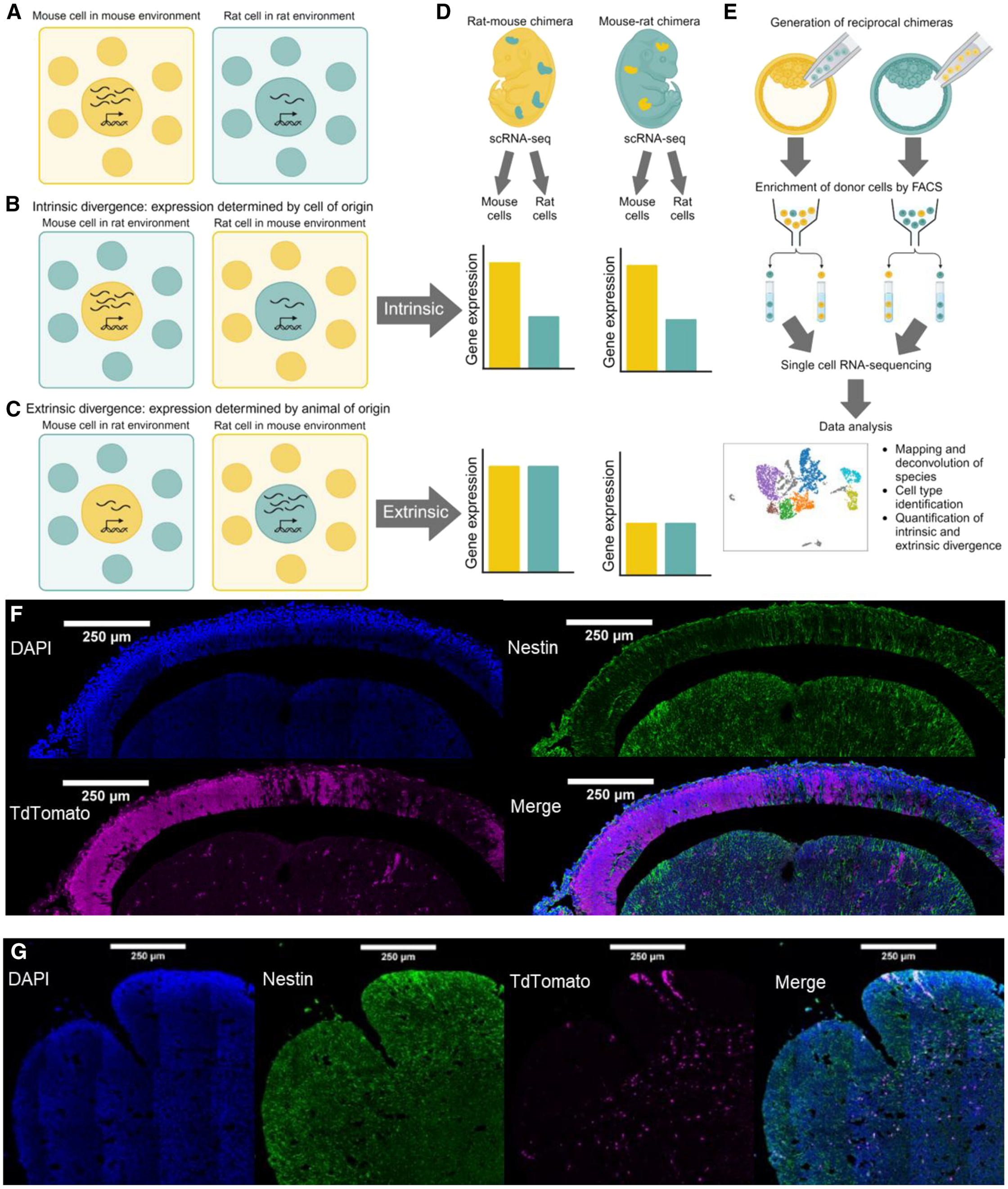

Figure 1. Conceptual model of estimating extrinsic and intrinsic divergence using reciprocal chimeras
(A) Rat and mouse cells in species-matched environments. In wild-type animals, the divergence in expression of a single gene between species is determined by divergence intrinsic to each cell as well as by divergence in the extracellular environment. In this example, expression is 2.5-fold higher in mouse than in rat. However, it is unclear how much of this divergence is cell-extrinsically vs. cell-intrinsically driven.
(B) Example of cell-intrinsic divergence. In rat/mouse chimeras, we can measure gene expression in mouse cells in a rat-like environment and in rat cells in a mouse-like environment. If the expression of the gene remains unchanged compared to species-matched environments, then the divergence must be due to intrinsic mechanisms.
(C) Example of cell-extrinsic divergence. If the expression of this gene in mouse cells in a rat-like environment matches the expression of the gene in rat cells in a rat-like environment and vice versa, then the divergence must be due to extrinsic divergence in the extracellular environment.
(D) Outline of measuring intrinsic and extrinsic divergence genome-wide. To measure extrinsic and intrinsic divergence, we can generate mouse-like (a small proportion of rat cells and a large proportion of mouse cells) and rat-like reciprocal chimeras. We can then use scRNA-seq to measure gene expression in individual cells across the four different species-environment combinations. If the divergence in expression is similar in both rat-like and mouse-like environments,

(legend continued on next page)

CellPress
OPEN ACCESS

modeling cellular networks and their responses to both genetic and environmental factors as well as for predicting how these factors contribute to complex traits and diseases. Understanding these highly interdependent layers of regulation will enhance our capacity to map genotype-phenotype relationships and to understand the multiscale interactions that govern cellular function in health and disease. Yet despite the importance of this distinction between cell-extrinsic and -intrinsic regulation, there is currently no general framework to quantitatively deconvolve traits into these components.

In contrast to the qualitative approach that has dominated the study of intrinsic/extrinsic factors for well over a century, the quantitative dissection of two distinct molecular mechanisms contributing to evolutionary divergence in gene expression—a key driver of evolutionary adaptation—has been extensively explored.[12,13] These mechanisms are known as *cis* and *trans*: *cis* refers to regulatory elements such as promoters and enhancers that affect nearby genes on the same chromosome, whereas *trans*-acting factors involve diffusible molecules such as transcription factors (TFs) or non-coding RNAs that can regulate genes throughout the genome.[13] A landmark study used *Drosophila* interspecies hybrids to disentangle the *cis*- and *trans*-regulatory contributions to gene expression divergence.[13] Since then, genome-wide *cis*- and *trans*-regulatory variation has been estimated for species across the tree of life, finding that the majority of variation within most species is caused by *trans*-acting mechanisms.[14–17] While *cis*-acting divergence is always cell intrinsic, both intrinsic and extrinsic factors can lead to *trans*-acting divergence between species, yet their relative contributions to phenotypic evolution remain unknown.

Here, we unify these concepts by introducing a quantitative genetic framework to disentangle the contributions of intrinsic factors, extrinsic factors, and their interactions to evolutionary divergence in gene expression and other cellular traits.

RESULTS

Cell-extrinsic and -intrinsic divergence in interspecies chimeras: Concepts

A chimera is an amalgamation of two sets of cells, donor and host. We define host cells as the cells derived from the injected blastocyst; when host cells make up a large majority, they are in species-matched environments similar to wild-type organisms (Figure 1A). Donor cells are the progeny of the cells that were injected into the blastocyst; when these are a small minority, they are in species-mismatched environments determined by the host (Figures 1B and 1C). Throughout, we use "donor cells" and "cells in species-mismatched environments" interchangeably and "host cells" and "cells in species-matched environments" interchangeably.

To illustrate the concept of extrinsic and intrinsic divergence in gene expression, we can consider an orthologous gene that is more highly expressed in mice than in rats. If mouse cells express the gene at the same level regardless of their extracellular environment, and likewise for rat cells, then we infer that the expression difference is determined by the cell of origin rather than its extracellular environment, suggesting cell-intrinsic divergence (Figure 1B). However, if donor and host cells express this gene at equal levels within each chimera, then we infer that this divergence is determined by the extracellular environment of the cells in that individual, suggesting extrinsic divergence in gene expression caused by divergence in the extracellular environment (Figure 1C). In practice, we can measure the intrinsic and extrinsic divergence in gene expression using single-cell RNA sequencing (scRNA-seq) of reciprocal interspecies chimeras (Figure 1D).

Cell-extrinsic and cell-intrinsic divergence in interspecies chimeras: Data

To explore this concept empirically, we first generated reciprocal rat-like and mouse-like chimeras via blastocyst injection (Figure 1E; STAR Methods). Importantly, we used only tissues with less than 8% donor contribution so that the extrinsic environment was primarily composed of host cells. As an example, we observed clear populations of host and donor cells (marked by TdTomato) in the forebrain of both mouse-like and rat-like chimeras (Figures 1F and 1G). Consistent with previous work, different brain regions showed distinct patterns, as donor cells were rarer and more evenly distributed in the ganglionic eminence compared to the developing neocortex in mouse-like chimeras (Figure 1G).[18]

Next, we enriched for donor cells from stage-matched embryos (embryonic day [E] 13.5 in mouse, E15.25 in rat) and then generated and analyzed scRNA-seq data from the forebrain and connective tissue/spinal cord of both mouse-like (i.e., made up primarily of mouse host cells) and rat-like chimeras (Figures 1E and S1 and S2; STAR Methods). After removing low-quality cells, identifying cell types, and filtering those with fewer than 10 cells in any sample type (host mouse, host rat, donor mouse, and donor rat), we retained 4,720 cells distributed across 11 cell types.

Cell-extrinsic and -intrinsic divergence in interspecies chimeras: A quantitative framework

With this dataset in hand, we developed a quantitative framework to decompose the divergence in gene expression between

then it is driven by intrinsic divergence. On the other hand, if expression is similar when mouse and rat cells are in the same extracellular environment but different when comparing cells from two different environments, it is driven by extrinsic divergence.

(E) Outline of experimental procedure for this study. Reciprocal chimeras were generated via blastocyst injection. Cells from mouse-like and rat-like chimeras were harvested at matched developmental stages (E13.5 and E15.25, respectively). Donor cells in species-mismatched environments were enriched using fluorescence-activated cell sorting using TdTomato as a marker, and scRNA-seq was performed. Each cell was then identified as mouse or rat based on RNA-seq reads, clustered, and further analyzed.

(F) Immunofluorescence image of the E13.5 forebrain of a mouse-like chimera. Nuclei are stained with DAPI (blue), and Nestin (a neural stem cell marker) is shown in green. TdTomato (magenta) marks donor rat cells in the mouse-like environment.

(G) Immunofluorescence image of the E15.25 ganglionic eminence of a rat-like chimera. In this case, TdTomato marks mouse cells in the rat-like environment.

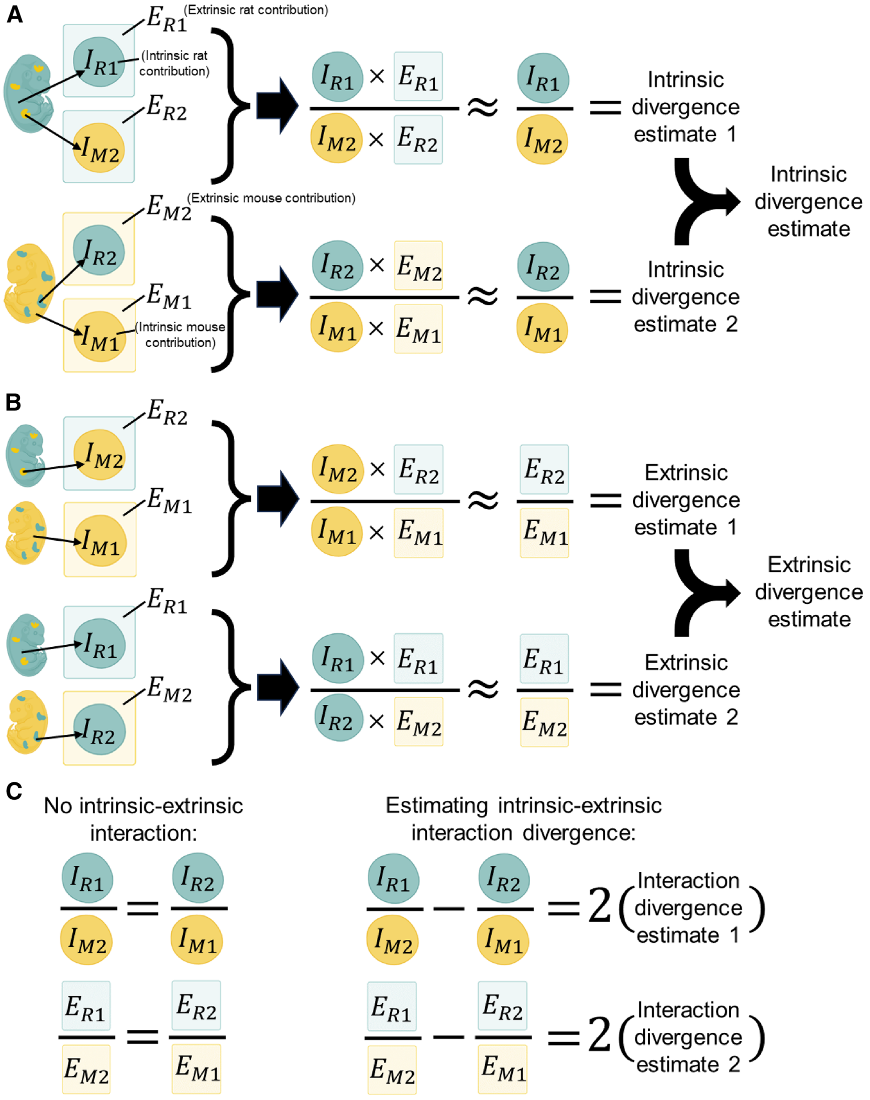

Figure 2. Quantitative framework for measuring intrinsic and extrinsic divergence

(A) Quantitative framework for measuring intrinsic divergence. In the absence of noise and interaction between intrinsic and extrinsic components, the effects of the rat-like environment on mouse and rat cells are identical, such that these extrinsic contributions cancel out, leaving only an estimate of the intrinsic divergence (top). Similarly, we can obtain another estimate of intrinsic divergence using the mouse-like chimeras, averaging the two estimates to obtain the final estimate of intrinsic divergence. Subscript numbers denote two independent estimates of the same underlying quantity, which may differ from each other due to measurement error and/or intrinsic-extrinsic interactions.

(B) Quantitative framework for measuring extrinsic divergence. In the absence of noise and interaction between intrinsic and extrinsic components, the intrinsic contributions of mouse cells are identical in both extracellular environments, such that they cancel out, leaving only an estimate of the extrinsic divergence (top). Similarly, we can obtain another estimate of extrinsic divergence using the other two measurements, averaging the two estimates to obtain the final estimate of extrinsic divergence.

(C) Quantitative framework for measuring intrinsic-extrinsic interaction divergence. In the absence of an interaction, the two intrinsic divergence estimates are equal and the two extrinsic divergence estimates are equal (left). However, if there is an intrinsic-extrinsic interaction, such that mouse cells do not respond to a rat environment in the same way as rat cells (or vice versa), then the two estimates are not equal. As a result, we can use the difference between the two intrinsic divergence estimates to measure interaction divergence and perform a similar procedure with the two extrinsic divergence estimates to obtain another estimate of interaction divergence (although in practice these two estimates are equal). For additional details, see STAR Methods.

mouse and rat into the extrinsic, intrinsic, and intrinsic-extrinsic interaction components of gene expression divergence for each gene (Figure 2; STAR Methods). In the absence of intrinsic-extrinsic interaction, the effects of the extracellular environment on mouse and rat cells are identical, enabling us to obtain two independent estimates of intrinsic divergence (Figure 2A). On the other hand, when comparing cells from the same species in different extracellular environments, the intrinsic contributions of the cells of that species are identical, enabling us to obtain two estimates of extrinsic divergence (Figure 2B). Any difference between these two estimates must be due to either measurement error or an interaction between extrinsic and intrinsic divergence (which is defined as extrinsic divergence in the host environments differing based on the genotype of the cell being measured and/or intrinsic divergence between cells of different genotypes differing based on the host genotype; see below and STAR Methods). Thus, we can use the four measurements obtained from reciprocal chimeras to decompose divergence in any cellular trait into the intrinsic, extrinsic, and interaction components. However, as the four empirical measurements of gene expression are generally easier to visually interpret than the values for the estimates of interaction, extrinsic, and intrinsic-extrinsic interaction, we plot the empirical measurements of gene expression (donor mouse expression, host mouse expression, donor rat expression, and host rat expression) throughout.

As an example, we can consider the expression of *Efnb3* in forebrain glutamatergic neurons (abbreviated as brain.glut.neu). In species-matched environments, we observed 2-fold higher expression in rat than in mouse (Figure 3A, left; the bars to the left of the dashed line are the same data as the bars for species-matched environments to the right of the dashed line and are included twice for clarity). However, in both chimeras, *Efnb3* expression in the donor cells mirrored that of the host

CellPress
OPEN ACCESS

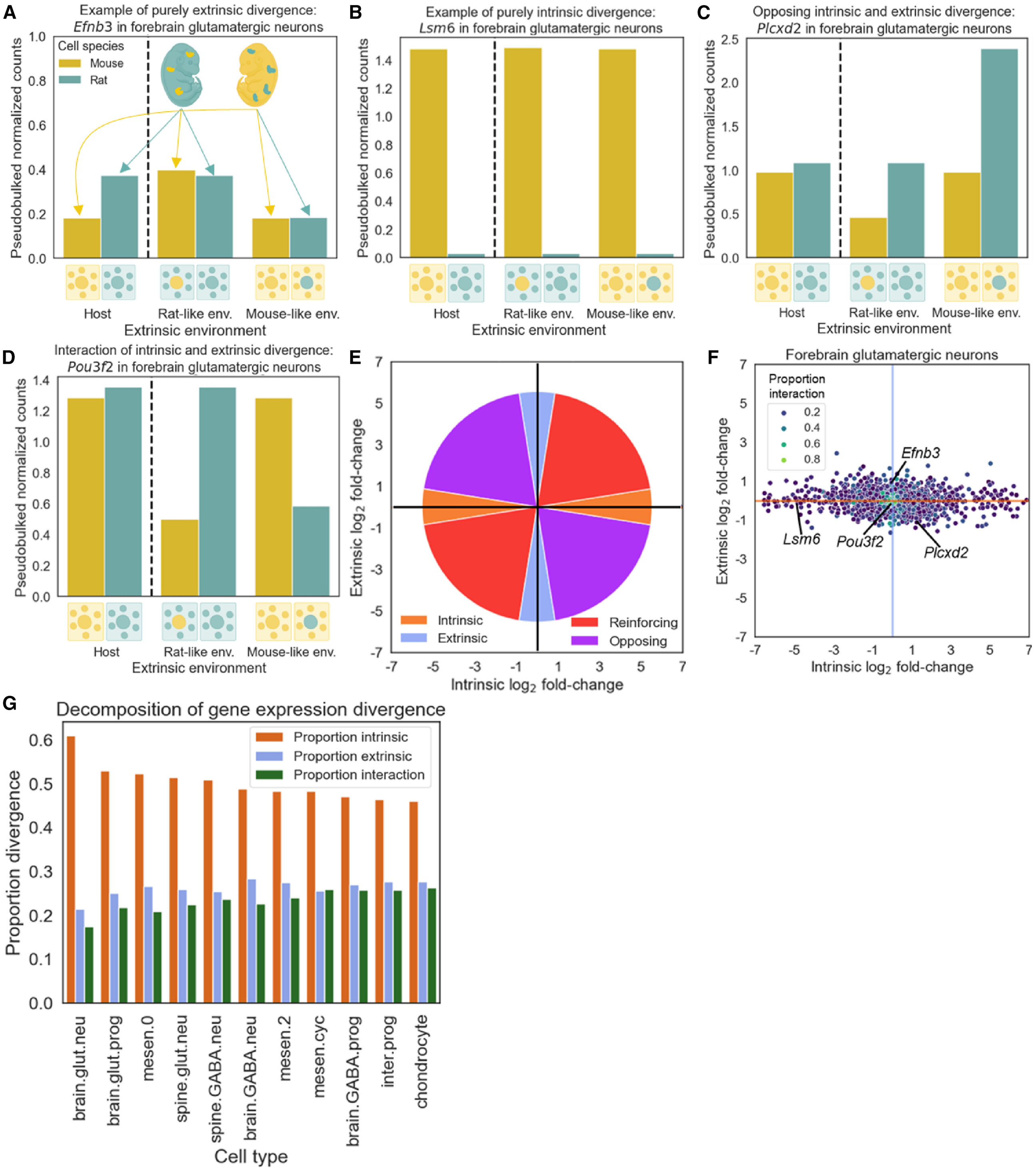

Figure 3. Decomposition of gene expression divergence into cell-intrinsic, cell-extrinsic, and interaction components

(A) Expression of *Efnb3* in forebrain glutamatergic neurons as an example of purely extrinsic divergence. On the left of the dashed line, the expression of *Efnb3* is shown in species-matched environments. To the right of the dashed line, expression in mouse cells and rat cells is first shown in a rat-like environment and then in a mouse-like environment. The data used for the bars in species-matched environments to the left of the dashed line are from the same host cells as the data in species-matched environments to the right of the dashed line and are included for clarity.

(B) Expression of *Lsm6* in forebrain glutamatergic neurons as an example of intrinsic divergence.

(C) Expression of *Plcxd2* as an example of opposing extrinsic and intrinsic divergence.

(legend continued on next page)

species (Figure 3A, middle/right; see Figure S3A for the per-cell normalized count distribution). This suggests that the divergence in the expression of *Efnb3* in forebrain glutamatergic neurons was driven almost entirely by extrinsic factors, which is reflected in its very high proportion of extrinsic divergence of 0.92. On the other hand, the expression of *Lsm6* was much higher in mouse cells regardless of their extracellular environment and had an intrinsic proportion divergence of 0.996, suggesting purely intrinsic divergence between species (Figures 3B and S3B).

However, most genes do not fit neatly into either category. For example, in both mouse and rat cells, *Plcxd2* had higher expression in a mouse-like environment than in a rat-like environment, suggesting extrinsic effects (Figures 3C and S3C). However, it also had higher expression in rat cells than in mouse cells in both chimeras, consistent with intrinsically higher expression in rat cells. These two opposing differences cancel out, resulting in nearly identical expression in host cells and approximately equal intrinsic and extrinsic proportions (0.46 and 0.53, Figure 3C) (see Figures S4–S9 and STAR Methods for a discussion of how developmentally dynamic gene expression and differences in the spatial distribution of donor cells between cell types might affect our results). While most genes primarily exhibit some combination of extrinsic and intrinsic divergence, some genes have evidence for an interaction between the two forms of divergence. For example, *Pou3f2* is downregulated in species-mismatched environments in both chimeras, which cannot be explained by any simple combination of intrinsic and extrinsic divergence, suggesting an interaction causing *Pou3f2* in mouse cells to respond differently to the extracellular environment than it does in rat cells (Figures 3D and S3D). This is reflected in *Pou3f2* having an interaction proportion of 0.85.

Cell-intrinsic divergence drives gene expression evolution

To summarize these results genome wide, we visualize these values on a scatterplot so that genes with purely intrinsic divergence are on the *x* axis and those with purely extrinsic divergence are on the *y* axis (Figures 3F, 3G, S10, and S11; Table S1). Across all cell types, we consistently observed that the genome-wide intrinsic component of gene expression divergence is larger than the extrinsic component, which is in turn larger than the interaction component (Figures 3G, S10, and S11). We estimate that 21%–28% of gene expression divergence is cell extrinsic across cell types, suggesting that a substantial fraction of the *trans*-regulatory divergence between species is due to differences in the extracellular environment.

We found that intrinsic divergence is substantially more correlated across cell types than extrinsic divergence (Figure S12A), consistent with the idea that *cis*-regulatory variation is also less tissue specific than *trans*.[19–21] Interestingly, neural and mesenchymal cell types clustered separately in terms of intrinsic divergence, but for extrinsic divergence they instead clustered by location within the embryo, resulting in spinal neurons clustering with other cells from more caudal parts of the embryo, such as mesenchyme (Figure S12B). This suggests that when specifically comparing extrinsically driven divergence, the similar extracellular environments shared between neuronal and non-neuronal caudal cell types could be the dominant factor, overriding the tendency for similar cell types, such as neurons, to cluster together. In addition, we evaluated whether several gene-level variables were correlated with intrinsic vs. extrinsic divergence proportions. The best predictor for individual genes was each gene's expression difference between mouse and rat: nearly all genes with >4-fold difference had primarily intrinsic divergence (Figure S12C), consistent with the finding that *cis*-regulatory divergence (which is cell intrinsic) contributes more to large changes in gene expression.[22] Other factors like expression level and tissue specificity had little to no predictive power (Figure S12D).

Cell-extrinsic and cell-intrinsic divergence can propagate through transcriptional networks

To investigate whether extrinsic divergence converges on particular pathways, we tested whether extrinsically driven genes were enriched for different gene sets, identifying dozens of enrichments (Table S2). For example, we found that genes involved in the endoplasmic reticulum (ER) stress response, such as *Hsp90b1*, had >2-fold enrichment for upregulation in a rat-like environment in 10 out of 11 cell types (Figures 4A, 4B, S13A, and S13B).[23] Conversely, we found that genes encoding histone methyltransferases, such as *Setbp1*, were highly enriched for upregulation in a mouse-like environment in neural but not connective tissue (Figures S14A–S14D).[24] Finally, many pathways were strongly enriched for extrinsic divergence in only one cell type, such as positive regulation of programmed cell death, which was specifically upregulated by forebrain GABAergic neurons in a rat-like environment (Figures S15A–S15D).

We next hypothesized that intrinsic and extrinsic regulation may propagate through transcriptional regulatory networks; for example, extrinsic regulation of a TF may result in extrinsic regulation of its target genes. In our analysis of ER stress response genes (Figure 4A) we noticed that divergence in the expression of *Xbp1*, a key transcriptional regulator of ER stress response genes,[25] was almost entirely extrinsic in some cell types, such as mesenchyme (Figures 4C and S16A). To determine whether extrinsically driven changes in *Xbp1* expression might drive the extrinsic divergence of its target genes, we compared the degree of extrinsic upregulation in the rat-like environment for *Xbp1*

(D) Expression of *Pou3f2* in forebrain glutamatergic neurons as an example of intrinsic-extrinsic interaction.
(E) Schematic outlining how each gene's location in (F) indicates intrinsic and extrinsic contributions to its expression divergence. Purely intrinsic divergence lies along the *x* axis, purely extrinsic divergence lies along the *y* axis, and a combination of both (which can be reinforcing or opposing in direction) occurs closer to the four diagonals.
(F) Plot of intrinsic and extrinsic divergence in forebrain glutamatergic neurons. Each point represents a gene and the example genes shown in (A)–(D) are indicated on the graph. Points are colored by the proportion of gene expression divergence explained by interaction.
(G) Bar plot showing the mean proportion of gene expression divergence explained by intrinsic divergence, extrinsic divergence, and their interaction across all measurable genes in each cell type.

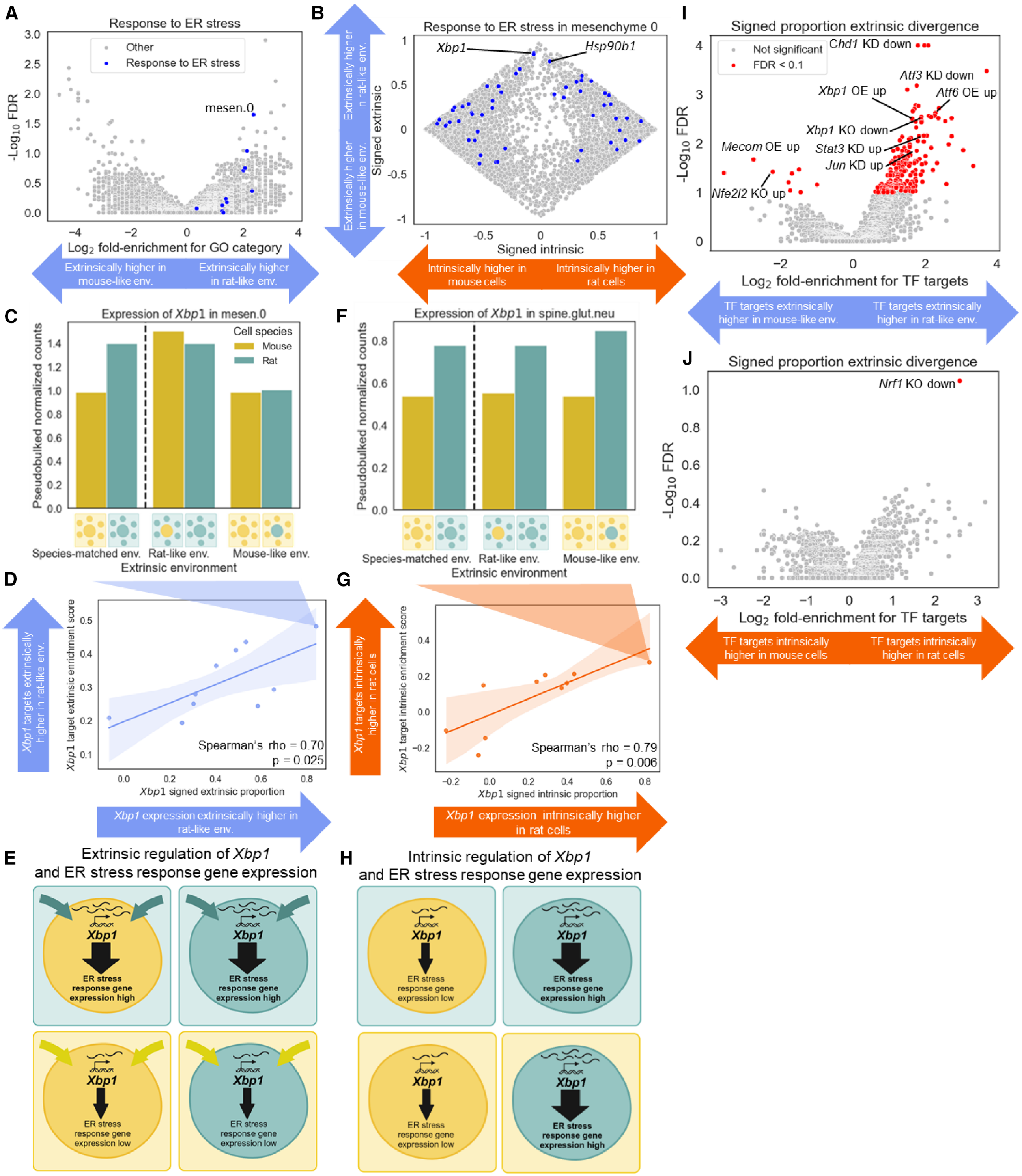

Figure 4. Extrinsic and intrinsic divergence in the expression of *Xbp1* and its target genes

(A) Enrichment of endoplasmic reticulum (ER) stress response genes for extrinsic divergence increasing expression in a rat-like environment. Each point is a Gene Ontology (GO) biological process category in a cell type and the points corresponding to the "response to ER stress" GO category are colored blue. The FDR is from the GSEAPY preranked test.

(B) Scatterplot showing signed proportion of intrinsic divergence (*x* axis) and signed proportion of extrinsic divergence (*y* axis) for all measurable genes in mesenchymal cluster 0. ER stress response genes are shown in blue and all other genes are shown in gray.

(legend continued on next page)

target genes (defined as genes upregulated by *Xbp1* overexpression)[26] with that of *Xbp1* itself in each cell type.[27] Remarkably, we observed a strong correlation between these two quantities, where cell types with extrinsic upregulation of *Xbp1* in the rat-like environment showed a similar pattern of extrinsic upregulation for its target genes (Spearman's rho = 0.70, p = 0.025, n = 10 cell types; Figure 4D). Moreover, we observed a similarly strong correlation between *Xbp1* extrinsic divergence and the extrinsic divergence of ER-stress-response-associated genes (Spearman's rho = 0.73, p = 0.014, n = 10 cell types; Figure S17A). This suggests that extrinsic regulation of *Xbp1* may lead to extrinsic regulation of its target genes (Figure 4E), though we cannot rule out a potential role for other unobserved factors such as post-transcriptional regulation[25] nor can we fully rule out an intrinsic cell-type-specific temporal shift in the expression of *Xbp1* that is somewhat specific to that gene (Figures S4–S9; STAR Methods).

Interestingly, although *Xbp1* had a similar magnitude of upregulation in both rat host spinal glutamatergic neurons and rat host mesenchyme compared to their mouse host counterparts (Figures 4C and 4F), this was almost entirely due to intrinsic divergence in the spinal neurons (Figures 4F and S16B). Analogous to the pattern for extrinsic divergence (Figure 4D), we found that cell types with intrinsic upregulation of *Xbp1* in rat cells showed a similar pattern of intrinsic upregulation of its target genes (Spearman's rho = 0.79, p = 0.006, n = 10 cell types; Figure 4G), although in this case *Xbp1* intrinsic regulation was not as strongly predictive of intrinsic upregulation of ER stress response genes more broadly (Spearman's rho = 0.37, p = 0.29, n = 10 cell types; Figure S17B). This suggests that intrinsically driven divergence in *Xbp1* expression may propagate to its target genes (Figure 4H) in much the same way as extrinsic divergence. However, it is worth noting that these results could be influenced by the spatial distribution of donor cells (Figures S4–S9; STAR Methods).

More generally, we expanded our analysis to test whether the known targets of many different TFs were enriched for unidirectional extrinsic divergence. Notably, the targets of several well-known regulators of ER stress, including *Atf3*, *Atf6*, and *Jun*, were also enriched for extrinsic upregulation in a rat-like environment, similar to *Xbp1* (Figure 4I).[28–30] The extrinsic regulation of these TFs was not correlated with that of *Xbp1* (Figure S18), suggesting divergence in distinct extrinsic factors that converge on TFs regulating the ER stress response. Overall, we observed 155 enriched TFs at a 10% false discovery rate (FDR), compared to just one enriched TF when performing an identical procedure with intrinsic regulation (Figure 4J; Table S3).

Complex patterns of intrinsic and extrinsic divergence underlie coordinated cell-type-specific expression of protein complex subunits

The single TF with targets enriched for intrinsic divergence was *Nfe2l1* (which encodes the protein Nrf1), a well-established regulator of proteasomal genes (Figure 4J).[31] For example, *Pomp*, a target of Nrf1 involved in the formation and activation of the proteasome, was consistently more highly expressed in rat cells than in mouse cells regardless of the extracellular environment (Figures S19A–S19F).[31] In contrast, the targets of other proteasome TFs, such as *Nfe2l2* (encoding the protein Nrf2) and *Stat3*, were enriched for extrinsic upregulation in mouse-like and rat-like environments, respectively (Figure 4I).[32,33] This heterogeneity in the mode (intrinsic vs. extrinsic) and direction (rat vs. mouse upregulation) of divergence in proteasomal TFs contrasted with the four ER stress response TFs mentioned above, whose targets were all enriched for extrinsic upregulation in a rat-like environment. Interestingly, the conflicting modes and directions of divergence of these proteasomal TFs did not correlate strongly with the divergence of their target genes (Figures S20A–S20C), suggesting that heterogeneity in the regulatory divergence of multiple TFs targeting the same genes might lead to more complex patterns than the relatively simple propagation seen for *Xbp1* and its targets.

To investigate the consequences of having multiple proteasomal TFs with differing modes and directions of expression divergence, we explored how proteasomal subunits were regulated across cell types. For example, in rat cells, the proteasomal subunit *Psmb1* had higher expression in a rat-like environment in forebrain GABAergic neurons, but higher expression in a mouse-like environment in spinal glutamatergic neurons and

(C) Expression of *Xbp1* in mesenchymal cluster 0.

(D) Correlation between signed extrinsic proportion of divergence for *Xbp1* (x axis) and the enrichment of its target genes for signed extrinsic proportion of divergence (y axis). Each point is a cell type. The line and shaded region represent the best fit and the 95% confidence interval of a linear model fit to the data. The p value is from the Spearman correlation.

(E) Conceptual model for *Xbp1*-driven increased expression of ER stress response genes in a rat-like environment occurring in some cell types. Due to an extrinsic factor(s), *Xbp1* expression is increased in a rat-like environment compared to a mouse-like environment. This then upregulates *Xbp1* target genes, many of which are ER stress response genes, in a rat-like environment.

(F) Expression of *Xbp1* in spinal glutamatergic neurons.

(G) Correlation between signed intrinsic proportion of divergence for *Xbp1* (x axis) and the enrichment of its target genes for signed intrinsic proportion of divergence (y axis). Each point is a cell type. The line and shaded region represent the best fit and the 95% confidence interval of a linear model fit to the data. The p value is from the Spearman correlation.

(H) Conceptual model for *Xbp1*-driven increased expression of ER stress response genes in rat cells occurring in some cell types. Due to an intrinsic factor(s), *Xbp1* expression is increased in rat cells compared to mouse cells. This then upregulates *Xbp1* target genes, many of which are ER stress response genes, in rat cells compared to mouse cells.

(I) Volcano plot of enrichment of TF target genes for signed extrinsic proportion of divergence. KO stands for knockout, KD stands for knockdown, OE stands for overexpression, up implies increased expression after the experimental manipulation, and down implies decreased expression after the experimental manipulation. The FDR is from the GSEAPY preranked test.

(J) Volcano plot of enrichment of TF target genes for signed intrinsic proportion of divergence. The FDR is from the GSEAPY preranked test.

CellPress
OPEN ACCESS

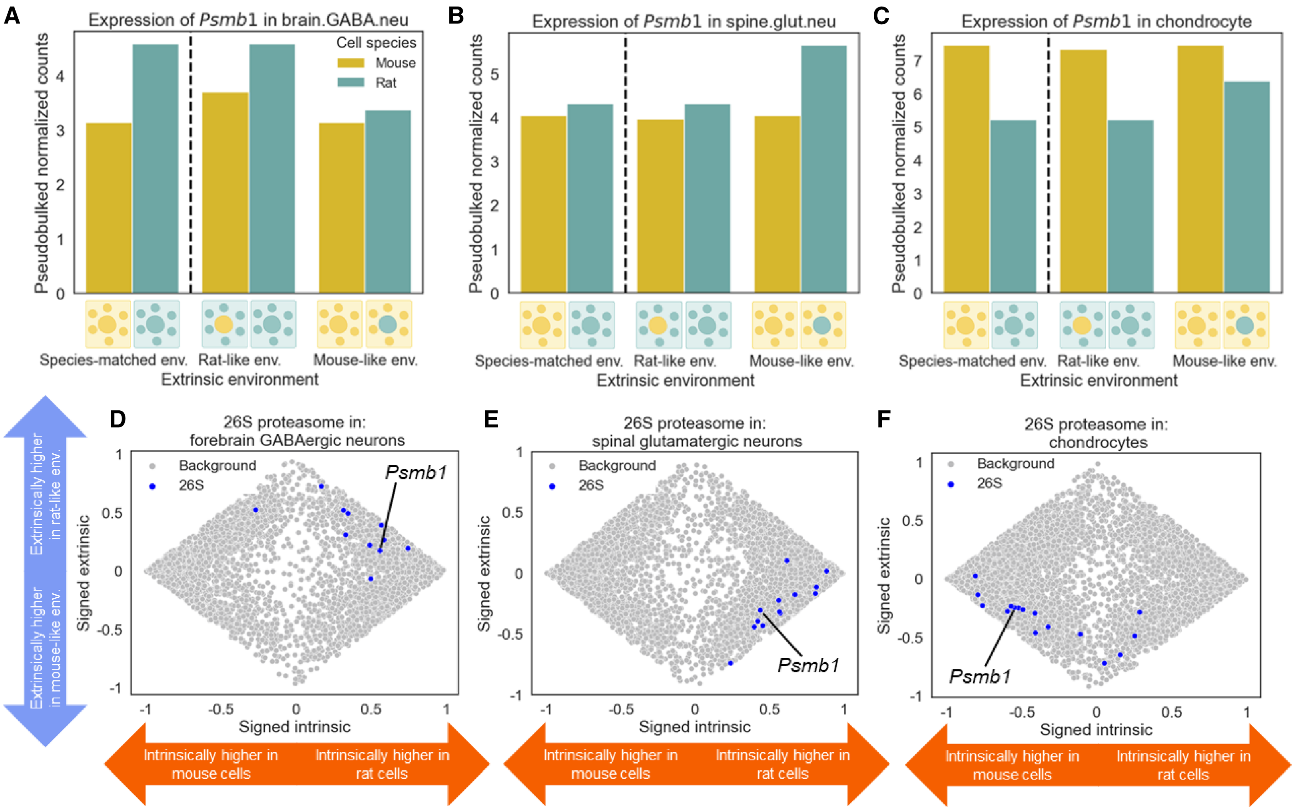

Figure 5. Cell-type-specific intrinsic and extrinsic divergence of the expression of genes encoding proteasomal subunits across cell types

(A) Expression of *Psmb1* in forebrain GABAergic neurons.

(B) Same as in (A) but for spinal glutamatergic neurons.

(C) Same as in (A) but for chondrocytes.

(D) Scatterplot showing signed proportion of intrinsic divergence (*x* axis) and signed proportion of extrinsic divergence (*y* axis) for all genes passing our filtering criteria for forebrain GABAergic neurons. Genes coding for 26S proteasomal subunits are shown in blue and all other genes are shown in gray.

(E) Same as in (D) but for spinal glutamatergic neurons.

(F) Same as in (D) but for chondrocytes.

chondrocytes (Figures 5A–5C and S21A–S21C). Interestingly, many proteasomal genes showed a similar pattern (Figures 5D–5F). In forebrain GABAergic neurons, expression was upregulated both intrinsically, in rat cells, and extrinsically, by the rat-like environment. However, in spinal glutamatergic neurons, the intrinsic effect was preserved but the extrinsic rat-like environment had the opposite effect, downregulating expression. Last, for chondrocytes, both effects were flipped compared to forebrain GABAergic neurons: both the intrinsic and the extrinsic effects of rat were repressive. Based on this finding, we looked for other gene sets that showed similarly variable enrichments across cell types and identified context-specific patterns of intrinsic and extrinsic components for spliceosomal genes as well as nuclear-encoded subunits of mitochondrial respiratory complex I (Figures S22 and S23). This suggests that highly coordinated context specificity, integrating both extrinsic and intrinsic signals, has evolved to regulate multiple protein complexes, perhaps to maintain proper stoichiometry while modulating their abundance in response to both extrinsic and intrinsic cues.

Cell-extrinsic divergence in the protein expression of two ER stress response genes

To test whether the extrinsically driven gene expression divergence we observed translates to the protein level, we used immunofluorescence (IF) to quantify extrinsic, intrinsic, and interaction components of divergence in protein levels for two proteins: *Jun* and *Hspa5*.[34] We prioritized these genes because they are involved in the ER stress response, show strong extrinsic divergence across cell types (especially in GABAergic forebrain neurons and progenitors; Figures 6A, 6B, and S24A–S24D), and are sufficiently highly expressed to be detected with IF. In the ganglionic eminences (which contain both GABAergic progenitors and recently born GABAergic neurons) of two mouse-like and two rat-like chimeras, we observed extrinsically driven upregulation in rat-like environments for both proteins, closely matching the estimates from our scRNA-seq data (Figures 6C–6E). This suggests that extrinsic divergence in mRNA abundances can translate to the protein level and also that our estimates of extrinsic divergence for these genes are unlikely to be inflated by experimental variability.

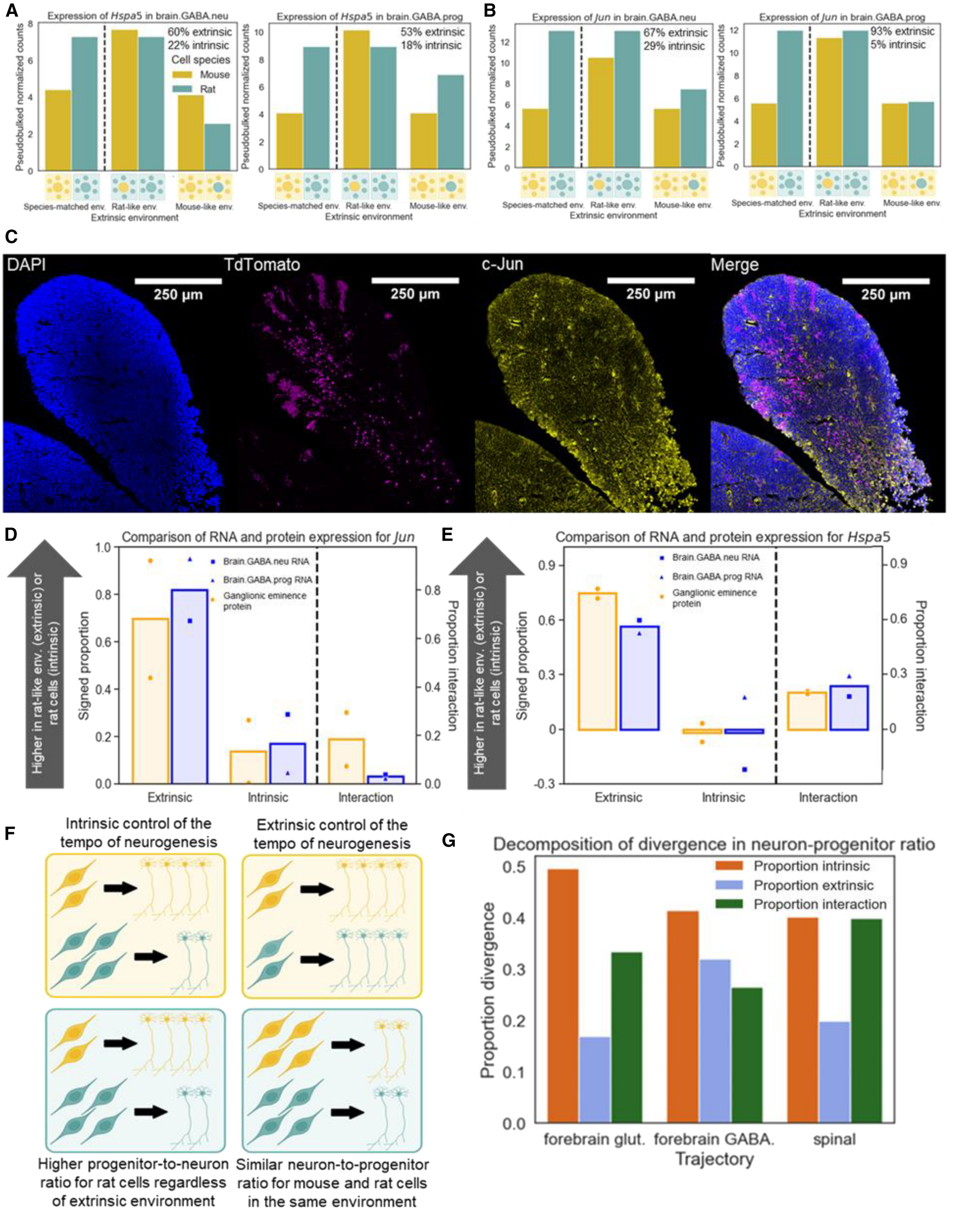

Figure 6. Extrinsic divergence of *Jun* and *Hspa5* at the RNA and protein levels

(A) Expression of *Hspa5* in forebrain GABAergic neurons (left) and progenitors (right). The percentage of gene expression divergence explained by extrinsic and intrinsic factors is shown in the upper right of each plot.

(legend continued on next page)

Extrinsic and intrinsic components of neural progenitor differentiation kinetics

As discussed above, our framework is applicable to most cellular traits. To demonstrate this, we explored the application of this framework to estimate extrinsic, intrinsic, and interaction components of a cellular-level quantitative trait: the ratio of post-mitotic neurons to neural progenitors, which reflects the timing of neurogenesis. If the timing of neurogenesis is intrinsically controlled, we would expect a higher neuron-to-progenitor ratio for mouse cells, regardless of their extracellular environment (Figure 6F, top). On the other hand, if this trait is extrinsically controlled, we would expect similar ratios for both mouse and rat cells in the same extracellular environment (Figure 6F, bottom). Strikingly, across three different branches of neurogenesis (forebrain GABAergic, forebrain glutamatergic, and spinal), we consistently found that the ratio of neurons to progenitors was much higher in mouse host cells than in rat donor cells, consistent with an intrinsic component for the faster development of mouse neurons and faster global maturation rate of mouse embryos (Figures S25A–S25C). However, we also found this ratio to be similar for mouse donor cells and rat host cells, consistent with previous work showing an effect of the rat maternal environment on the timing of neurogenesis (Figures S25A–S25C).[9] Overall, we estimated an average contribution of 44% intrinsic, 23% extrinsic, and 33% interaction for divergence in the timing of mouse and rat neuronal differentiation (Figure 6G).

Imprinted genes are strongly misexpressed in species-mismatched environments

During our analysis, we noticed that several imprinted genes had strikingly large interaction components. For example, while the imprinted gene *Grb10* was highly expressed in both mouse and rat chondrocytes when in species-matched environments, its expression was nearly zero in both mouse and rat chondrocytes in species-mismatched environments (Figures 7A and S26A).[35] In GABAergic progenitors, *Grb10* expression in mouse cells was unaffected by the extracellular environment but was undetectable in rat cells in a mouse-like environment (Figures 7B and S26B). In contrast, *Grb10* expression was unaltered in species-mismatched environments in forebrain GABAergic neurons (Figures 7C and S26C). This stark divergence was not restricted to *Grb10*, as, for example, *Igf2* is more highly expressed in rat chondrocytes than in mouse chondrocytes in species-matched environments, but drops close to zero when rat chondrocytes are in a mouse-like environment (Figures S27A and S27B).[36]

To explore whether this pattern holds for imprinted genes more generally, we tested whether previously reported imprinted genes were enriched for interactions between extrinsic and intrinsic divergence. Remarkably, we observed that imprinted genes were strongly enriched for large interaction components ($p < 0.001$ in both connective and nervous tissue; Figure 7D). Given that genes expressed from the maternal allele (maternally expressed) and expressed from the paternal allele (paternally expressed) often have opposing effects on growth, we split imprinted genes based on the expressed allele.[37] We found that paternally expressed genes were strongly enriched for higher expression in species-mismatched environments ($p < 0.01$ in both brain and connective tissue), whereas maternally expressed genes were enriched for higher expression in species-matched environments only in connective tissue ($p < 0.01$ in connective tissue; $p > 0.5$ in brain tissue), suggesting that maternally expressed and paternally expressed genes are often affected by species-mismatched environments in opposite ways (Figure 7E). The pattern for paternally expressed genes is exemplified by *Zdbf2*, which is expressed approximately 2-fold higher in species-mismatched environments for both mouse cells and rat cells across connective and nervous cell types (Figures S27C–S27F).[38]

To determine if this pattern of disrupted imprinting generalized to other types of cells and chimeras, we sought to test for this effect in other chimera scRNA-seq datasets. Because these studies do not have reciprocal chimeras, we could not disentangle the extrinsic and interaction components, but we could test the simpler prediction that imprinted genes should be highly sensitive to the extracellular environment. In one previous study, rat-mouse chimeras were generated in which the hearts were almost entirely derived from rat donor cells, and their gene expression was compared to wild-type rat heart cells from the same embryonic stage.[39] Despite the data being independently generated using different host and donor strains, tissues, and developmental stages, we observed that *Grb10* and *Igf2* were highly expressed in wild-type rat but were nearly undetectable in rat cells present in a mouse-like environment across all 11 heart data cell types tested (Figures 7F, S28A, and S28B). More generally, imprinted genes were strongly enriched for differential expression between rat cells in a rat-like environment

(B) The same as in (A) but showing the expression of *Jun*, which codes for the protein c-Jun.

(C) Immunofluorescent staining for c-Jun in the medial ganglionic eminence of a rat-like chimera. Donor mouse cells are marked with TdTomato, and c-Jun staining is shown in yellow.

(D) Comparison of intrinsic, extrinsic, and interaction divergence at the RNA and protein levels for *Jun*. To the left of the dashed line, the signed proportion of divergence is along the *y* axis, with larger values indicating a greater proportion of either intrinsic or extrinsic divergence leading to higher expression in rat. The extrinsic and intrinsic divergence for protein levels across two chimera pairs is shown in blue, and the divergence in RNA expression is in orange for GABAergic neurons (square markers) and neurons (triangle markers). The bars indicate the average across replicates for protein and the two cell types for RNA. To the right of the dashed line, the proportion of interaction divergence (which is non-negative by definition) is shown with its own *y* axis.

(E) Similar to (D) but showing *Hspa5* divergence.

(F) Conceptual model for extrinsic or intrinsic divergence in the pace of neurogenesis. The left outlines intrinsic divergence, as mouse cells include more neurons than progenitors regardless of whether they are in a rat-like or a mouse-like environment. The right outlines extrinsic divergence, in which the ratio of neurons to progenitors is similar for mouse and rat cells in the same extracellular environment but differs between environments.

(G) Proportion of intrinsic, extrinsic, and interaction divergence for neuron-progenitor ratio in forebrain glutamatergic neurogenesis, forebrain GABAergic neurogenesis, and spinal GABAergic and glutamatergic neurogenesis together.

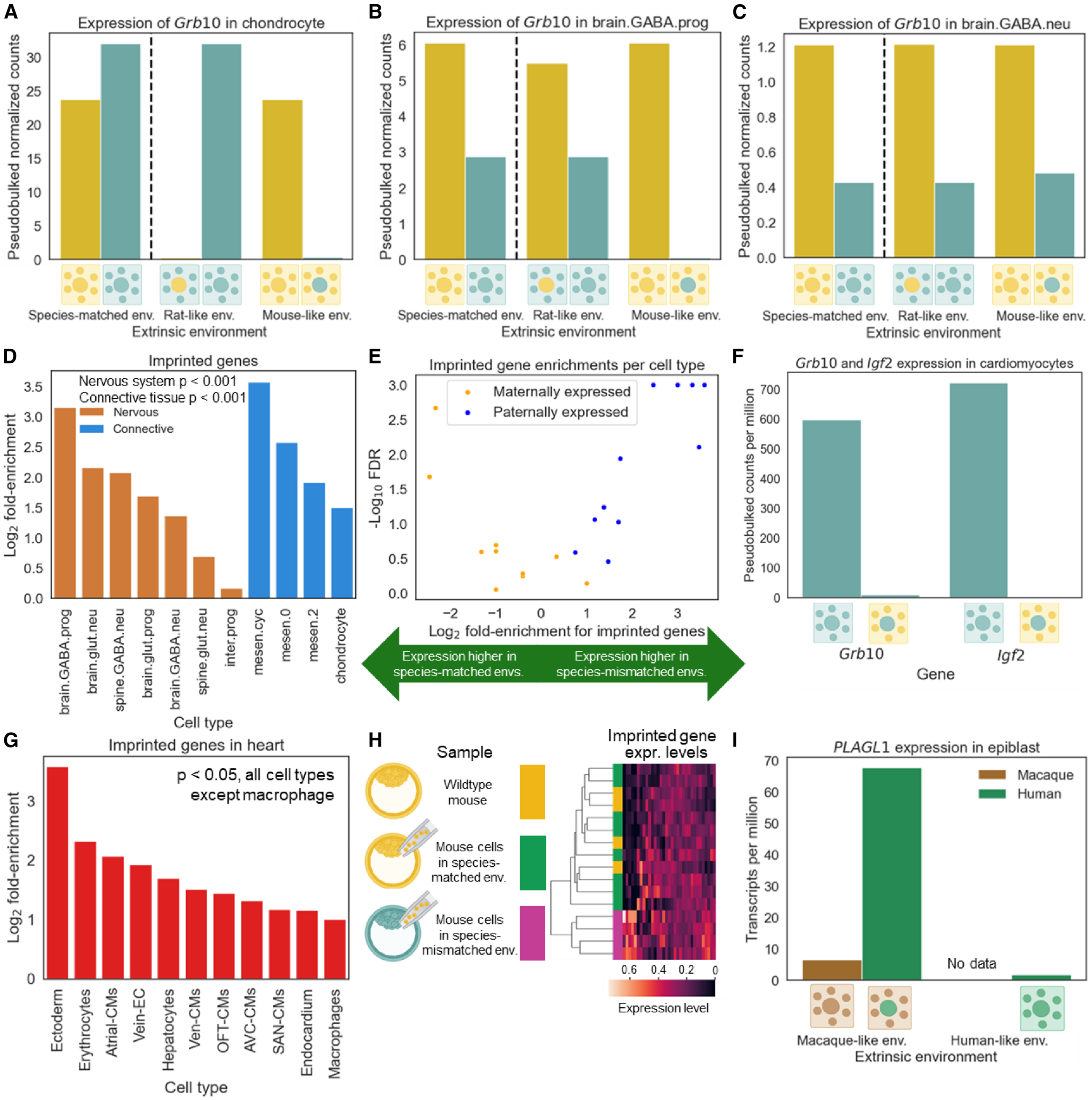

Figure 7. Misexpression of imprinted genes in species-mismatched environments

(A) Expression of the imprinted gene *Grb10* in chondrocytes.

(B) Expression of *Grb10* in forebrain GABAergic progenitors.

(C) Expression of *Grb10* in forebrain GABAergic neurons.

(D) Enrichment of imprinted genes for high absolute interaction divergence across cell types. *p* value is from the GSEAPY preranked test.

(E) Enrichment of maternally expressed and paternally expressed genes for signed interaction divergence. Each of the 11 cell types analyzed in this study is represented by one blue point and one orange point. A negative enrichment score indicates higher expression in species-matched environments, whereas a positive enrichment score indicates higher expression species-mismatched environments. FDR is from the GSEAPY preranked test.

(F) Expression of imprinted genes *Grb10* and *Igf2* in ventricular cardiomyocytes.

(G) The same as in (D), but for cell types in the heart dataset and using the absolute log fold change between expression in a mouse-like environment and expression in a rat-like environment to rank genes when computing enrichments.

(H) Heatmap of imprinted gene expression across samples for adult parathyroid data. Samples were hierarchically clustered using the Euclidean distance metric.

(I) Expression of the imprinted gene *PLAGL1* in macaque and human epiblasts. Data for macaque cells in a human-like environment have not been reported.

vs. rat cells in a mouse-like environment ($p < 0.05$ in all cell types except macrophages, where $p = 0.11$; Figure 7G).

One possible explanation for disrupted imprinting in donor cells is that imprinting could already be disrupted in the embryonic stem cells (ESCs) from which these cells are derived. To test this, we examined a published bulk RNA-seq dataset of adult parathyroid gland cells where the same mouse ESCs were injected into both rat and mouse blastocysts and compared to wild-type mice with no blastocyst injection.[40] If imprinting disruption is due to the species-mismatched environment rather than any technical factor related to cell lines or chimera generation, then we would expect imprinted genes to be misexpressed in rat-mouse chimeras but not in mouse-mouse chimeras. To evaluate this, we hierarchically clustered all samples based on the expression of all expressed imprinted genes. We found that samples from species-mismatched environments clearly clustered together, whereas samples from wild-type mice and donor mouse cells in a species-matched environment were intermixed in a separate cluster (Figure 7G). For example, *Grb10* and *Igf2* were strongly misexpressed in a species-mismatched environment (Figure S29). Overall, these results suggest that a species-mismatched environment, not blastocyst injection of ESCs more generally, underlies misexpression of imprinted genes and that misexpression can persist into adulthood.

Finally, we investigated whether imprinting may also be disrupted in *ex vivo* human-macaque chimeric embryos.[41,42] We found that many imprinted genes, such as *PLAGL1*, showed strong misregulation in human cells growing in a macaque-like embryo (Figure 7I). Imprinted genes were enriched for differential expression between human cells and macaque cells in the same macaque-like environment, as well as between human cells in a human-like environment and those in a macaque-like environment ($p < 0.01$ in each). These results suggest that imprinting disruption in chimeras does not depend on the maternal environment (which is not present for *ex vivo* chimeras) and occurs in both rodents and primates. Overall, our results suggest that the interaction between extrinsic and intrinsic divergence results in widespread misexpression of imprinted genes in interspecies chimeras.

DISCUSSION

Despite widespread interest in the concept of cell autonomy for over a century,[1,2] and numerous fundamental discoveries,[3–9] its investigation has remained a qualitative and low-throughput endeavor. Building on this foundational body of work, we have developed a broadly applicable framework to decompose divergence in any cellular trait into cell-extrinsic and cell-intrinsic components (Figure 2). This enables not only mathematical precision but also the potential for high-throughput measurement of intrinsic and extrinsic contributions to thousands of traits in a single experiment.

Application of this framework to gene expression divergence between mouse and rat revealed a preponderance of intrinsic factors in the evolution of gene expression (Figure 3). However, extrinsic factors also contribute to many genes and even dominate the divergence of some critical pathways, such as the ER stress response in mesenchyme (Figure 4). The contributions of extrinsic factors are often highly cell-type specific, as exemplified by *Xbp1*'s regulation of the ER stress response as well as by the proteasome (Figure 5), histone methyltransferases (Figure S14), and regulators of cell death (Figure S15). This cell-type specificity enabled us to track the propagation of extrinsically driven divergence through transcriptional networks (Figure 4) as well as to the protein level (Figure 6).

In addition, we identified widespread misregulation of imprinted genes as a result of interactions between extrinsic and intrinsic divergence (Figure 7), suggesting that imprinting may be controlled by a complex interplay between rapidly evolving intrinsic and extrinsic factors. This misregulation may be the result of a direct (e.g., ligand-receptor) interaction between extrinsic and intrinsic mechanisms for controlling imprinted gene expression or, alternatively, could involve a more general cellular response to a species-mismatched environment. Regardless of the exact mechanism, it points to interesting, largely unexplored biology. In addition, since many imprinted genes play key roles in cell proliferation and survival, modulating their expression could be a promising avenue to improve the function of donor cells in interspecies chimeras.[43] It will be interesting to explore the effects of xenotransplantation on the expression of imprinted genes as well.

Our framework can also provide insight into the genetic basis of interspecies chimeric incompatibility.[44] For example, we identified strong extrinsic divergence in ER stress response genes such as *Hspa5* (Figure 6E), which is vital for neuronal survival during development,[45] as well as in histone methyltransferases (Figure S14), which play a key role in controlling the tempo of neuronal development.[46] Consistent with this, we found that the tempo of neurogenesis—which is faster in mouse than in rat—has evolved via a complex combination of both intrinsic and extrinsically driven divergence, as well as interactions between these. Interestingly, although the intrinsic:extrinsic ratio was similar for neuronal differentiation (Figure 6G) and global gene expression (Figure 3G)—about 2:1—neuronal differentiation showed a substantially greater contribution from intrinsic-extrinsic interaction. Further work will be required to confirm this finding across different species and developmental stages and to disentangle effects such as maternal environment[9] from the host embryo's genotype. Nevertheless, it is tempting to speculate that this difference may be related to the greater complexity of cellular differentiation compared to individual gene expression levels and that increasingly complex traits may show a general trend toward greater intrinsic-extrinsic interaction.

A recent study reported rat-mouse chimeras where rat ESCs were injected into host mouse blastocysts that were unable to generate a forebrain (*Hesx1*$^{-/-}$).[18] This study is challenging to directly compare to ours, since donor cells composed ~60% of the forebrain at the time of scRNA-seq profiling, suggesting that the extracellular environment was neither mouse-like nor rat-like. In addition, the study did not have reciprocal chimeras or a quantitative analysis framework to disentangle extrinsic/intrinsic divergence. Perhaps because of these and other factors, our results differ substantially from theirs. Specifically,

they found the pace of neurodevelopment (at a gross anatomical level) in rat donor cells to be extrinsically controlled, while the overall transcriptome was intrinsically determined. In contrast, using reciprocal chimeras and our quantitative framework, we found both neurodevelopment (at a single-cell level) and the transcriptome to be subject to about twice as much intrinsic as extrinsic divergence, along with a substantial effect of interaction between these, especially for neurodevelopment.

Our study opens the door to myriad avenues for further research. While our initial study focused on gene expression, the same framework can be applied to any cellular trait. For example, we can now quantify the cell-intrinsic vs. cell-extrinsic contributions to the evolution of numerous components of gene regulation, such as chromatin accessibility, DNA methylation, histone post-translational modifications, 3D chromosomal contacts, splicing, translation, etc. In addition, questions about the cell autonomy of cellular morphology, electrophysiology, differentiation dynamics, cell fate decisions, somatic mutations, cell death, and protein subcellular localization can begin to be quantitatively addressed. Looking ahead, an important question will be how the interplay between intrinsic and extrinsic divergence leads to phenotypic divergence at the organismal level. For example, a recent study linked extrinsically driven changes in neuronal firing to differences in behavior between oldfield and deer mice.[47]

Other exciting future directions include sampling chimeras across a time course of development, which will allow us to disentangle the role of developmental time from intrinsic and extrinsic divergence to better understand the evolution of developmentally dynamic gene expression. In addition, our framework could be applied in conjunction with spatial transcriptomics to further decompose extrinsic divergence into local components driven by cell-cell contacts vs. non-local (e.g., organ-wide) components driven by diffusible factors such as signaling molecules. Finally, our approach can be applied to any pair of strains or species compatible with chimerism. Overall, the application of our framework to a wide range of cellular traits will enable new insights into the mechanistic basis of cellular function, developmental processes, and phenotypic evolution across the tree of multicellular life.

Limitations of the study

There are several important limitations to our study that can be addressed in future work. First, we sampled a relatively small number of cells from only two animals per tissue. As a result, our estimates of intrinsic, extrinsic, and interaction divergence are imprecise for low-expressed genes (such as *Efnb3* or *Lsm6*) although likely fairly accurate for more highly expressed genes (such as *Jun*, *Hspa5*, *Grb10*, and *Igf2*, evidenced by the orthogonal replication of their expression patterns). In addition, it is unclear how consistent these results are from chimera to chimera, outside of the imprinted gene misexpression that we have replicated in other datasets. It is also important to note that measurement error and animal-to-animal variability are expected to primarily increase the intrinsic-extrinsic interaction component and, to a lesser extent, the extrinsic component, because they involve more potential sources of measurement error than the intrinsic component. Another limitation is that we are generally unable to disentangle gene-specific temporal shifts in gene expression from intrinsic, extrinsic, and interaction divergence (Figures S4–S9; STAR Methods). Although our analysis suggests that this generally does not have a large effect on our results, an important future direction will be sampling a developmental time course in chimeras to further disentangle temporal shifts in gene expression from other forms of divergence.

RESOURCE AVAILABILITY

Lead contact

Requests for further information and resources and reagents should be directed to and will be fulfilled by the lead contact, Hunter Fraser (hbfraser@stanford.edu).

Materials availability

This study did not generate new unique reagents.

Data and code availability

Sequencing data are available through GEO with accession no. GEO: GSE266218. All code needed to reproduce the analyses described in this study is available on Zenodo: https://doi.org/10.5281/zenodo.15263429.

ACKNOWLEDGMENTS

We thank Jonathan Pritchard, Richard Schneider, Vanessa Barrone, Leslie Magtanong, Gabriella Cale, Alina Xiao, Daniel Pederick, Tom Hindmarsh-Sten, Colleen McLaughlin, Liqun Luo, and other members of the Fraser and Luo labs for helpful discussions and feedback. We thank the Stanford Functional Genomics core facility for preparing the scRNA-seq libraries. We thank Xabier López Aranguren and Asier Ullate Agote for sharing metadata for the heart embryonic dataset. Biorender was used to generate some figures. Funding was provided by NIH R01DK121851 (awarded to H.N.), the Japan Agency for Medical Research and Development (AMED) grant no. JP22bm1004002 (awarded to H.N.), NIH R01HG012285 (Could you please let us know whether Ref. 58 is a book or journal?awarded to H.B.F.), and JSPS KAKENHI grant no. 21H02378 (awarded to T.N.). A.L.S. was supported by an NDSEG fellowship under grant no. FA9550-21-F-0003. K.J.I. was supported by a National Science Foundation Graduate Research fellowship under grant no. DGE-1656518.

AUTHOR CONTRIBUTIONS

T.N. led all wet-lab work including cell culture, chimera generation and assessment of donor contribution, fluorescence-activated cell sorting (FACS), preparation of cells for input for scRNA-seq, and immunofluorescence. K.J.I. helped with FACS and sampling of chimeric embryos. C.F. helped with immunofluorescent staining and optimization. A.L.S. performed all bioinformatic analysis, visualization, validation, and writing of software, with guidance from H.B.F. A.L.S. wrote the manuscript and created figures with input from H.B.F., T.N., and H.N. A.L.S. and T.N. wrote the methods section with input from H.B.F. and H.N. H.B.F. and H.N. provided funding and conceived the study.

DECLARATION OF INTERESTS

H.N. is a co-founder and shareholder in ReproCELL, Megakaryon, and Century Therapeutics.

STAR★METHODS

Detailed methods are provided in the online version of this paper and include the following:

- KEY RESOURCES TABLE

- EXPERIMENTAL MODEL AND SUBJECT DETAILS
  - Animals
  - ESC culture
  - Embryo culture and manipulation
- METHOD DETAILS
  - Chimera dissection and cell preparation
  - Flow cytometry
  - Single cell RNA-sequencing
  - Histological analysis
- STATISTICAL ANALYSIS AND QUANTIFICATION
  - Alignment, species deconvolution, and clustering
  - Gene filtering, pseudobulking, and count normalization
  - A framework to decompose gene expression divergence into extrinsic, intrinsic, and interaction components
  - Data processing and analysis for predictors of intrinsic and extrinsic divergence
  - Enrichment analysis
  - Transcription factor target enrichment analysis
  - Quantification and analysis of immunofluorescence (IF) images
  - Analysis of neuron-progenitor ratios
  - Analysis of imprinted gene expression
  - Analysis of bulk RNA-seq data and developmentally dynamic gene expression
  - The spatial distribution of donor cells could lead to differences in estimates of intrinsic and extrinsic divergence between cell types

SUPPLEMENTAL INFORMATION

Supplemental information can be found online at https://doi.org/10.1016/j.xgen.2025.100891.

Received: August 3, 2024
Revised: January 10, 2025
Accepted: May 5, 2025
Published: May 29, 2025

REFERENCES

1. Roux, W. (1887). Beiträge zur Entwickelungsmechanik des Embryos. I. Zur Orientirung über einige Probleme der organischen Entwickelung. Z. Biol. *21*, 411–524.
2. Schlosser, G. (2024). From "self-differentiation" to organoids—the quest for the units of development. Dev. Genes Evol. *234*, 57–64. https://doi.org/10.1007/s00427-023-00711-z.
3. Spemann, H., and Mangold, H. (2001). Induction of embryonic primordia by implantation of organizers from a different species. 1923. Int. J. Dev. Biol. *45*, 13–38.
4. Le Douarin, N.M. (1980). The ontogeny of the neural crest in avian embryo chimaeras. Nature *286*, 663–669. https://doi.org/10.1038/286663a0.
5. Twitty, V.C. (1936). Correlated genetic and embryological experiments on Triturus. I and II. J. Exp. Zool. *74:*, 232–309.
6. Schneider, R.A. (2018). Neural crest and the origin of species-specific pattern. genesis *56*, e23219. https://doi.org/10.1002/dvg.23219.
7. Le Douarin, N., and McLaren, A. (1984). Chimeras in Developmental Biology (Academic Press).
8. Suchy, F., and Nakauchi, H. (2018). Interspecies chimeras. Curr. Opin. Genet. Dev. *52*, 36–41. https://doi.org/10.1016/j.gde.2018.05.007.
9. Stepien, B.K., Naumann, R., Holtz, A., Helppi, J., Huttner, W.B., and Vaid, S. (2020). Lengthening Neurogenic Period during Neocortical Development Causes a Hallmark of Neocortex Expansion. Curr. Biol. *30*, 4227–4237.e5. https://doi.org/10.1016/j.cub.2020.08.046.
10. Eames, B.F., and Schneider, R.A. (2005). Quail-duck chimeras reveal spatiotemporal plasticity in molecular and histogenic programs of cranial feather development. Dev. Camb. Engl. *132*, 1499–1509. https://doi.org/10.1242/dev.01719.
11. Schneider, R.A., and Helms, J.A. (2003). The Cellular and Molecular Origins of Beak Morphology. Science *299*, 565–568. https://doi.org/10.1126/science.1077827.
12. Wittkopp, P.J., and Kalay, G. (2012). Cis-regulatory elements: molecular mechanisms and evolutionary processes underlying divergence. Nat. Rev. Genet. *13*, 59–69. https://doi.org/10.1038/nrg3095.
13. Wittkopp, P.J., Haerum, B.K., and Clark, A.G. (2004). Evolutionary changes in cis and trans gene regulation. Nature *430*, 85–88. https://doi.org/10.1038/nature02698.
14. Coolon, J.D., McManus, C.J., Stevenson, K.R., Graveley, B.R., and Wittkopp, P.J. (2014). Tempo and mode of regulatory evolution in *Drosophila*. Genome Res. *24*, 797–808. https://doi.org/10.1101/gr.163014.113.
15. Liu, X., Li, Y.I., and Pritchard, J.K. (2019). Trans Effects on Gene Expression Can Drive Omnigenic Inheritance. Cell *177*, 1022–1034.e6. https://doi.org/10.1016/j.cell.2019.04.014.
16. Albert, F.W., Bloom, J.S., Siegel, J., Day, L., and Kruglyak, L. (2018). Genetics of trans-regulatory variation in gene expression. Elife *7*, e35471. https://doi.org/10.7554/eLife.35471.
17. Wang, Q., Jia, Y., Wang, Y., Jiang, Z., Zhou, X., Zhang, Z., Nie, C., Li, J., Yang, N., and Qu, L. (2019). Evolution of cis- and trans-regulatory divergence in the chicken genome between two contrasting breeds analyzed using three tissue types at one-day-old. BMC Genom. *20*, 933. https://doi.org/10.1186/s12864-019-6342-5.
18. Huang, J., He, B., Yang, X., Long, X., Wei, Y., Gao, Y., Fang, Y., Ying, W., Wang, Z., Li, C., et al. (2023). Interspecies blastocyst complementation generates functional rat cell-derived forebrain tissues in mice. Developmental Biology *187*, 2129–2142. https://doi.org/10.1101/2023.04.13.536774.
19. Kim-Hellmuth, S., Aguet, F., Oliva, M., Muñoz-Aguirre, M., Kasela, S., Wucher, V., Castel, S.E., Hamel, A.R., Viñuela, A., Roberts, A.L., et al. (2020). Cell type–specific genetic regulation of gene expression across human tissues. Science *369*, eaaz8528. https://doi.org/10.1126/science.aaz8528.
20. The, G.T.E.C., Aguet, F., Anand, S., Ardlie, K.G., Gabriel, S., Getz, G.A., Graubert, A., Hadley, K., Handsaker, R.E., Huang, K.H., et al. (2020). The GTEx Consortium atlas of genetic regulatory effects across human tissues. Science *369*, 1318–1330. https://doi.org/10.1126/science.aaz1776.
21. GTEx Consortium (2017). Genetic effects on gene expression across human tissues. Nature *550*, 204–213. https://doi.org/10.1038/nature24277.
22. Metzger, B.P.H., Duveau, F., Yuan, D.C., Tryban, S., Yang, B., and Wittkopp, P.J. (2016). Contrasting Frequencies and Effects of *cis* - and *trans* -Regulatory Mutations Affecting Gene Expression. Mol. Biol. Evol. *33*, 1131–1146. https://doi.org/10.1093/molbev/msw011.
23. Hoter, A., El-Sabban, M., and Naim, H. (2018). The HSP90 Family: Structure, Regulation, Function, and Implications in Health and Disease. Int. J. Mol. Sci. *19*, 2560. https://doi.org/10.3390/ijms19092560.
24. Zhu, Y., Sun, D., Jakovcevski, M., and Jiang, Y. (2020). Epigenetic mechanism of SETDB1 in brain: implications for neuropsychiatric disorders. Transl. Psychiatry *10*, 115. https://doi.org/10.1038/s41398-020-0797-7.
25. Park, S.-M., Kang, T.-I., and So, J.-S. (2021). Roles of XBP1s in Transcriptional Regulation of Target Genes. Biomedicines *9*, 791. https://doi.org/10.3390/biomedicines9070791.
26. Gregor, M.F., Misch, E.S., Yang, L., Hummasti, S., Inouye, K.E., Lee, A.-H., Bierie, B., and Hotamisligil, G.S. (2013). The role of adipocyte XBP1 in metabolic regulation during lactation. Cell Rep. *3*, 1430–1439. https://doi.org/10.1016/j.celrep.2013.03.042.
27. Subramanian, A., Tamayo, P., Mootha, V.K., Mukherjee, S., Ebert, B.L., Gillette, M.A., Paulovich, A., Pomeroy, S.L., Golub, T.R., Lander, E.S., et al. (2005). Gene set enrichment analysis: A knowledge-based approach for interpreting genome-wide expression profiles. Proc. Natl. Acad. Sci. *102*, 15545–15550. https://doi.org/10.1073/pnas.0506580102.

28. Ku, H.-C., and Cheng, C.-F. (2020). Master Regulator Activating Transcription Factor 3 (ATF3) in Metabolic Homeostasis and Cancer. Front. Endocrinol. *11*, 556. https://doi.org/10.3389/fendo.2020.00556.

29. Adachi, Y., Yamamoto, K., Okada, T., Yoshida, H., Harada, A., and Mori, K. (2008). ATF6 is a transcription factor specializing in the regulation of quality control proteins in the endoplasmic reticulum. Cell Struct. Funct. *33*, 75–89. https://doi.org/10.1247/csf.07044.

30. Yu, W., Wang, B., Zhou, L., and Xu, G. (2021). Endoplasmic Reticulum Stress-Mediated p62 Downregulation Inhibits Apoptosis via c-Jun Upregulation. Biomol. Ther. *29*, 195–204. https://doi.org/10.4062/biomolther.2020.089.

31. Sha, Z., and Goldberg, A.L. (2014). Proteasome-Mediated Processing of Nrf1 Is Essential for Coordinate Induction of All Proteasome Subunits and p97. Curr. Biol. *24*, 1573–1583. https://doi.org/10.1016/j.cub.2014.06.004.

32. Vangala, J.R., Dudem, S., Jain, N., and Kalivendi, S.V. (2014). Regulation of PSMB5 Protein and β Subunits of Mammalian Proteasome by Constitutively Activated Signal Transducer and Activator of Transcription 3 (STAT3). J. Biol. Chem. *289*, 12612–12622. https://doi.org/10.1074/jbc.M113.542829.

33. He, F., Ru, X., and Wen, T. (2020). NRF2, a Transcription Factor for Stress Response and Beyond. Int. J. Mol. Sci. *21*, 4777. https://doi.org/10.3390/ijms21134777.

34. Wang, J., Lee, J., Liem, D., and Ping, P. (2017). HSPA5 Gene encoding Hsp70 chaperone BiP in the endoplasmic reticulum. Gene *618*, 14–23. https://doi.org/10.1016/j.gene.2017.03.005.

35. Juan, A.M., Foong, Y.H., Thorvaldsen, J.L., Lan, Y., Leu, N.A., Rurik, J.G., Li, L., Krapp, C., Rosier, C.L., Epstein, J.A., et al. (2022). Tissue-specific Grb10/Ddc insulator drives allelic architecture for cardiac development. Mol. Cell *82*, 3613–3631.e7. https://doi.org/10.1016/j.molcel.2022.08.021.

36. Giannoukakis, N., Deal, C., Paquette, J., Goodyer, C.G., and Polychronakos, C. (1993). Parental genomic imprinting of the human IGF2 gene. Nat. Genet. *4*, 98–101. https://doi.org/10.1038/ng0593-98.

37. Moore, G.E., Ishida, M., Demetriou, C., Al-Olabi, L., Leon, L.J., Thomas, A. C., Abu-Amero, S., Frost, J.M., Stafford, J.L., Chaoqun, Y., et al. (2015). The role and interaction of imprinted genes in human fetal growth. Philos. Trans. R. Soc. B Biol. Sci. *370*, 20140074. https://doi.org/10.1098/rstb.2014.0074.

38. Glaser, J., Iranzo, J., Borensztein, M., Marinucci, M., Gualtieri, A., Jouhanneau, C., Teissandier, A., Gaston-Massuet, C., and Bourc'his, D. (2022). The imprinted Zdbf2 gene finely tunes control of feeding and growth in neonates. Elife *11*, e65641. https://doi.org/10.7554/eLife.65641.

39. Coppiello, G., Barlabé, P., Moya-Jódar, M., Abizanda, G., Pogontke, C., Barreda, C., Iglesias, E., Linares, J., Arellano-Viera, E., Larequi, E., et al. (2023). Generation of heart and vascular system in rodents by blastocyst complementation. Dev. Cell *58*, 2881–2895.e7. https://doi.org/10.1016/j.devcel.2023.10.008.

40. Kano, M., Mizuno, N., Sato, H., Kimura, T., Hirochika, R., Iwasaki, Y., Inoshita, N., Nagano, H., Kasai, M., Yamamoto, H., et al. (2023). Functional calcium-responsive parathyroid glands generated using single-step blastocyst complementation. Proc. Natl. Acad. Sci. *120*, e2216564120. https://doi.org/10.1073/pnas.2216564120.

41. Zhou, F., Wang, R., Yuan, P., Ren, Y., Mao, Y., Li, R., Lian, Y., Li, J., Wen, L., Yan, L., et al. (2019). Reconstituting the transcriptome and DNA methylome landscapes of human implantation. Nature *572*, 660–664. https://doi.org/10.1038/s41586-019-1500-0.

42. Tan, T., Wu, J., Si, C., Dai, S., Zhang, Y., Sun, N., Zhang, E., Shao, H., Si, W., Yang, P., et al. (2021). Chimeric contribution of human extended pluripotent stem cells to monkey embryos ex vivo. Cell *184*, 2020–2032.e14. https://doi.org/10.1016/j.cell.2021.03.020.

43. Piedrahita, J.A. (2011). The role of imprinted genes in fetal growth abnormalities. Birt. Defects Res. A. Clin. Mol. Teratol. *91*, 682–692. https://doi.org/10.1002/bdra.20795.

44. Masaki, H., and Nakauchi, H. (2017). Interspecies chimeras for human stem cell research. Development *144*, 2544–2547. https://doi.org/10.1242/dev.151183.

45. Ramani, B., Rose, I.V.L., Pan, A., Tian, R., Ma, K., Palop, J.J., and Kampmann, M. (2023). Scalable, cell type-selective, AAV-based *in vivo* CRISPR screening in the mouse brain. BiorXiv. https://doi.org/10.1101/2023.06.13.544831.

46. Ciceri, G., Baggiolini, A., Cho, H.S., Kshirsagar, M., Benito-Kwiecinski, S., Walsh, R.M., Aromolaran, K.A., Gonzalez-Hernandez, A.J., Munguba, H., Koo, S.Y., et al. (2024). An epigenetic barrier sets the timing of human neuronal maturation. Nature *626*, 881–890. https://doi.org/10.1038/s41586-023-06984-8.

47. Niepoth, N., Merritt, J.R., Uminski, M., Lei, E., Esquibies, V.S., Bando, I.B., Hernandez, K., Gebhardt, C., Wacker, S.A., Lutzu, S., et al. (2024). Evolution of a novel adrenal cell type that promotes parental care. Nature *629*, 1082–1090. https://doi.org/10.1038/s41586-024-07423-y.

48. Cardoso-Moreira, M., Halbert, J., Valloton, D., Velten, B., Chen, C., Shao, Y., Liechti, A., Ascenção, K., Rummel, C., Ovchinnikova, S., et al. (2019). Gene expression across mammalian organ development. Nature *571*, 505–509. https://doi.org/10.1038/s41586-019-1338-5.

49. Zeng, T., Spence, J.P., Mostafavi, H., and Pritchard, J.K. (2024;56:1632–1643). Bayesian estimation of gene constraint from an evolutionary model with gene features. Nature Genetics. https://doi.org/10.1101/2023.05.19.541520.

50. The ENCODE Project Consortium, Abascal, F., Acosta, R., Addleman, N. J., Adrian, J., Afzal, V., Ai, R., Aken, B., Akiyama, J.A., Jammal, O.A., et al. (2020). Expanded encyclopaedias of DNA elements in the human and mouse genomes. Nature *583*, 699–710. https://doi.org/10.1038/s41586-020-2493-4.

51. Wolf, F.A., Angerer, P., and Theis, F.J. (2018). SCANPY: large-scale single-cell gene expression data analysis. Genome Biol. *19*, 15. https://doi.org/10.1186/s13059-017-1382-0.

52. Korsunsky, I., Millard, N., Fan, J., Slowikowski, K., Zhang, F., Wei, K., Baglaenko, Y., Brenner, M., Loh, P., and Raychaudhuri, S. (2019). Fast, sensitive and accurate integration of single-cell data with Harmony. Nat. Methods *16*, 1289–1296. https://doi.org/10.1038/s41592-019-0619-0.

53. Traag, V.A., Waltman, L., and Van Eck, N.J. (2019). From Louvain to Leiden: guaranteeing well-connected communities. Sci. Rep. *9*, 5233. https://doi.org/10.1038/s41598-019-41695-z.

54. Fang, Z., Liu, X., and Peltz, G. (2023). GSEApy: a comprehensive package for performing gene set enrichment analysis in Python. Bioinformatics *39*, btac757. https://doi.org/10.1093/bioinformatics/btac757.

55. Schindelin, J., Arganda-Carreras, I., Frise, E., Kaynig, V., Longair, M., Pietzsch, T., Preibisch, S., Rueden, C., Saalfeld, S., Schmid, B., et al. (2012). Fiji: an open-source platform for biological-image analysis. Nat. Methods *9*, 676–682. https://doi.org/10.1038/nmeth.2019.

56. Ying, Q.-L., Stavridis, M., Griffiths, D., Li, M., and Smith, A. (2003). Conversion of embryonic stem cells into neuroectodermal precursors in adherent monoculture. Nat. Biotechnol. *21*, 183–186. https://doi.org/10.1038/nbt780.

57. Yamaguchi, T., Hamanaka, S., and Nakauchi, H. (2014). The generation and maintenance of rat induced pluripotent stem cells. Methods Mol. Biol. Clifton NJ *1210*, 143–150. https://doi.org/10.1007/978-1-4939-1435-7_11.

58. Nagy, A., Gertsenstein, M., Vintersten, K., and Behringer, R. (2003). Manipulating the Mouse Embryo: A Laboratory Manual, Third Edition (Cold Spring Harbor Laboratory Press).

59. Yates, A.D., Allen, J., Amode, R.M., Azov, A.G., Barba, M., Becerra, A., Bhai, J., Campbell, L.I., Carbajo Martinez, M., Chakiachvili, M., et al. (2022). Ensembl Genomes 2022: an expanding genome resource for non-vertebrates. Nucleic Acids Res. *50*, D996–D1003. https://doi.org/10.1093/nar/gkab1007.

CellPress
OPEN ACCESS

60. Di Bella, D.J., Habibi, E., Stickels, R.R., Scalia, G., Brown, J., Yadollahpour, P., Yang, S.M., Abbate, C., Biancalani, T., Macosko, E.Z., et al. (2021). Molecular logic of cellular diversification in the mouse cerebral cortex. Nature *595*, 554–559. https://doi.org/10.1038/s41586-021-03670-5.

61. Lipiec, M.A., Bem, J., Koziński, K., Chakraborty, C., Urban-Ciećko, J., Zajkowski, T., Dąbrowski, M., Szewczyk, Ł.M., Toval, A., Ferran, J.L., et al. (2020). TCF7L2 regulates postmitotic differentiation programs and excitability patterns in the thalamus. Development *147*, 190181. https://doi.org/10.1242/dev.190181.

62. Witschi, R., Johansson, T., Morscher, G., Scheurer, L., Deschamps, J., and Zeilhofer, H.U. (2010). *Hoxb8-Cre* mice: A tool for brain-sparing conditional gene deletion. genesis *48*, 596–602. https://doi.org/10.1002/dvg.20656.

63. Lee, J., Rabbani, C.C., Gao, H., Steinhart, M.R., Woodruff, B.M., Pflum, Z. E., Kim, A., Heller, S., Liu, Y., Shipchandler, T.Z., et al. (2020). Hair-bearing human skin generated entirely from pluripotent stem cells. Nature *582*, 399–404. https://doi.org/10.1038/s41586-020-2352-3.

64. Yanai, I., Benjamin, H., Shmoish, M., Chalifa-Caspi, V., Shklar, M., Ophir, R., Bar-Even, A., Horn-Saban, S., Safran, M., Domany, E., et al. (2005). Genome-wide midrange transcription profiles reveal expression level relationships in human tissue specification. Bioinforma. Oxf. Engl. *21*, 650–659. https://doi.org/10.1093/bioinformatics/bti042.

65. Virtanen, P., Gommers, R., Oliphant, T.E., Haberland, M., Reddy, T., Cournapeau, D., Burovski, E., Peterson, P., Weckesser, W., Bright, J., et al. (2020). SciPy 1.0: fundamental algorithms for scientific computing in Python. Nat. Methods *17*, 261–272. https://doi.org/10.1038/s41592-019-0686-2.

66. Giurgiu, M., Reinhard, J., Brauner, B., Dunger-Kaltenbach, I., Fobo, G., Frishman, G., Montrone, C., and Ruepp, A. (2019). CORUM: the comprehensive resource of mammalian protein complexes—2019. Nucleic Acids Res. *47*, D559–D563. https://doi.org/10.1093/nar/gky973.

67. Rouillard, A.D., Gundersen, G.W., Fernandez, N.F., Wang, Z., Monteiro, C. D., McDermott, M.G., and Ma'ayan, A. (2016). The harmonizome: a collection of processed datasets gathered to serve and mine knowledge about genes and proteins. Database *2016*, baw100. https://doi.org/10.1093/database/baw100.

68. Ashburner, M., Ball, C.A., Blake, J.A., Botstein, D., Butler, H., Cherry, J.M., Davis, A.P., Dolinski, K., Dwight, S.S., Eppig, J.T., et al. (2000). Gene Ontology: tool for the unification of biology. Nat. Genet. *25*, 25–29. https://doi.org/10.1038/75556.

STAR★METHODS

KEY RESOURCES TABLE

REAGENT or RESOURCE	SOURCE	IDENTIFIER
Antibodies		
APC-anti-mouse CD45	Biolegend	Cat# 103112; RRID: AB_312977
PE-Cy7-anti rat CD45	Biolegend	Cat#: 202214; RRID: AB_2253306
Anti-c-Fos 1:1000 Dilution	Abcam	Cat#: ab190289; RRID: AB_2737414
Anti-c-Jun 1:100 Dilution	Abcam	Cat#: ab31419; RRID: AB_731605
Anti-Hspa5 1:1000 Dilution	Abcam	Cat#: ab21685; RRID: AB_2119834
Anti-Nestin 1:500 Dilution	Novus Bio	Cat#: NB100-1604; RRID: AB_2282642
Anti-tdTomato 1:100 Dilution	Origene	Cat#: AB8181-200; RRID: AB_3206272
Donkey anti-rabbit IgG Alexa Fluor 647 1:1000 Dilution	Thermo Fisher Scientific	Cat#: A-31573; RRID: AB_2536183
Goat anti-guinea pig IgG Alexa Fluor 647 1:1000 Dilution	Thermo Fisher Scientific	Cat#: A-21450; RRID: AB_141882
Donkey anti-goat IgG Alexa Fluor 594 1:1000 Dilution	Thermo Fisher Scientific	Cat#: A-11058; RRID: AB_142540
Donkey anti-chicken IgG Alexa Fluor 555 1:1000 Dilution	Thermo Fisher Scientific	Cat#: A-78949; RRID: AB_2921071
Donkey anti-guinea pig IgG Alexa Fluor 488 1:1000 Dilution	Jackson Immuno	Cat#: 706-545-148; RRID: AB_2340472
Donkey anti-chicken IgG Alexa Fluor 488 1:1000 Dilution	Thermo Fisher Scientific	Cat#: A-78948; RRID: AB_2921070
Chemicals, peptides, and recombinant proteins		
LIF	Peprotech	300-05
MEK inhibitor PD0325901	Tocris	4192
GSK3 inhibitor CHIR99021	Tocris	4423
KSOM-AA medium	CytoSpring	K0101
Collagenase/Dispase	Roche	10269638001
Critical commercial assays		
10x 3' v3.1 scRNA-seq kit	10x Genomics	PN-1000268
Deposited data		
GRCm39	ENSEMBL v109	ENSEMBL: https://ftp.ensembl.org/pub/release-109/fasta/mus_musculus_cbaj/
mRatBN7.2	ENSEMBL v109	ENSEMBL: https://ftp.ensembl.org/pub/release-109/fasta/rattus_norvegicus/
Bulk RNA-seq from developing mouse and rat	Cardoso-Moreira et al.[48]	EvoDevoApp: https://apps.kaessmannlab.org/evodevoapp/
Constraint on individual genes	Zeng et al[49]	https://doi.org/10.1038/s41588-024-01820-9
ENCODE cCREs	The ENCODE Project Consortium et al.[50]	ENCODE: https://screen.encodeproject.org/
List of imprinted genes in mouse and human	N/A	https://www.geneimprint.com/site/home
Chimeric rodent heart scRNA-seq data	Coppiello et al.[39]	GEO: GSE236400
Chimeric rodent parathyroid scRNA-seq data	Kano et al.[40]	GEO: GSE232600
Chimeric human-macaque *in vitro* embryo scRNA-seq data	Tan et al.[42]	GEO: GSE155381
Human *in vitro* embryo scRNA-seq data	Zhou et al.[41]	GEO: GSE109555
Chimeric rodent brain and connective tissue scRNA-seq data	This study	GEO: GSE266218
Experimental models: Cell lines		
SUN106.2 mouse ESCs	Nakauchi Lab	N/A
SGE2 mouse ESCs	Nakauchi Lab	N/A
Rat ESCs	Nakauchi Lab	N/A

(*Continued on next page*)

Continued		
REAGENT or RESOURCE	SOURCE	IDENTIFIER
Experimental models: Organisms/strains		
Wistar rats	Charles River Laboratory	CRL: 003
CD1 mice	Jackson Laboratories	ME: 000664
Software and algorithms		
CellRanger v7.1.0	10x Genomics	N/A
Scanpy v1.9.5	Wolf et al. 2018[51]	N/A
Harmony (python implementation) v0.0.9	Korunsky et al.[52]	N/A
Leiden algorithm v0.10.2	Traag et al.[53]	N/A
GSEAPY v1.0.6	Fang et al.[54]	N/A
ImageJ	Schindelin et al.[55]	https://imagej.net/software/fiji/downloads
Custom code used for this study	This study	Zenodo: https://doi.org/10.5281/zenodo.15263429
Other		
Piezo-driven micromanipulator	Prime Tech	N/A
FACS Aria II	BD	N/A
Optimal Cutting Temperature compound	Sakura Finetek	4583

EXPERIMENTAL MODEL AND SUBJECT DETAILS

Animals

Seven-week-old Wistar female rats and 10-week-old male rats were purchased from Charles River Laboratory (Wilmington, MA; CRL: 003). Seven-week-old CD1 female mice and 10-week-old male mice (CRL: 022) were purchased from Charles River Laboratory. Seven-week-old C57BL/6 female mice and 10-week-old male mice were purchased from Jackson Laboratories (Bar Harbor, ME: 000664). Littermates of the same sex were randomly assigned to experimental groups. All rats and mice were housed in pathogen-free conditions with free access to food and water. All animal protocols were approved by the Administrative Panel on Laboratory Animal Care at Stanford University.

ESC culture

Undifferentiated tdTomato-labelled or GFP-labeled mouse ESCs (SUN106.2 and SGE2) were maintained on mitomycin-c treated mouse embryonic fibroblasts (MEFs) in N2B27 medium[56] containing 1000 U/ml LIF (Peprotech, Cranbury, NJ; 300-05), 1 μM MEK inhibitor PD0325901 (Tocris, Barton Ln, Abingdon, United Kingdom; 4192) and 3 μM GSK3 inhibitor CHIR99021 (Tocris: 4423). Undifferentiated tdTomato-labelled rat ESCs were maintained in N2B27 medium containing 1 μM MEK inhibitor PD0325901, 3 μM CHIR99021, and 1000 U/ml of rat LIF as described.[57] Their pluripotency was confirmed by chimera generation assay. All PSC lines used in this study were male lines.

Embryo culture and manipulation

Wild-type mouse embryos were prepared according to published protocols.[58] In brief, morula-stage embryos were obtained by uterus perfusion from superovulated CD1 mice at 2.5 days postcoitum (dpc). Morula-stage embryos were cultured in KSOM-AA medium (CytoSpring, Mountain View, CA; K0101) for 1 day and developed to blastocyst-stage embryos. Wild-type rat blastocysts were obtained by uterus perfusion from female rats at 4.5 dpc and cultured in Rat KSOM medium. For micromanipulation, mouse or rat ESCs were trypsinized and suspended in mouse or rat ESC culture medium. A piezo-driven micromanipulator (Prime Tech, Tsuchiura, Japan) was used to pierce the zona pellucida and trophectoderm under microscopy and 5–7 mouse or rat ESCs were introduced into blastocyst cavities near the inner cell mass. After blastocyst injection, embryos were cultured for 1–2 hours. Mouse blastocysts were then transferred into uteri of pseudopregnant recipient CD1 female mice at 2.5 dpc. Rat blastocysts were then transferred into uteri of pseudopregnant recipient Wistar female rats at 3.5 dpc. Table S4 shows results of the cell injection.

METHOD DETAILS

Chimera dissection and cell preparation

Embryonic day (E) 15.25 mouse-rat and E13.5 rat-mouse chimeras were dissected to harvest forebrain and connective tissue. In brief, chimeric fetuses were collected from either E15.25 rat or E13.5 mouse pregnant mothers and forebrain and connective tissues were harvested from those chimeric fetuses. Both forebrains and connective tissues were dissociated in the solution containing 2mg/ml Collagenase/Dispase (Roche, Basel, Switzerland; 10269638001) in Hanks' Balanced Salt solution. After 30-60 min

incubation at 37 degrees, 10% fetal bovine serum (FBS) in PBS solution was added into the tissue solution to inactivate the enzymes. All the dissociated cells were then filtered and used for the subsequent experiment.

Flow cytometry

Dissociated cells derived from forebrain and connective tissue were stained with APC-anti-mouse CD45 antibody (Biolegend, San Diego, CA; 103112), PE-Cy7-anti rat CD45 (Biolegend: 202214). Donor chimerism was analyzed by detecting CD45 negative, tdTomato or GFP-expressing cells (detailed in Table S5). Both donor and host cells in the forebrain and connective tissue of chimeric fetuses were sorted using a FACS Aria II (BD, Franklin Lakes, NJ).

Single cell RNA-sequencing

The chimeric fetuses from the CD1-Wistar chimeras that presented less than 8% donor contribution to either forebrain or connective tissue were used for single cell RNA-sequencing (Table S4). The connective tissue and forebrain of three rat-like chimeras, three mouse-like chimeras, one wildtype mouse, and one wildtype rat were used for scRNA-seq using 10x 3' v3.1 chemistry. The connective tissue and forebrain were from different chimeras but pooled together before the cells entered the 10x controller. Of the resulting eight libraries, the rat-like chimera in library MR1 had high quality data for forebrain and connective tissue, the mouse-like chimera in library RM1 had sufficiently high quality data for the neuronal lineage, the mouse-like chimera in library RM2 had sufficiently high quality data for connective tissue, and both the wildtype mouse and rat libraries had sufficiently high quality data for both tissue types.

Histological analysis

Tissues were fixed with 4% paraformaldehyde and embedded in Optimal Cutting Temperature (O.C.T.) compound (Sakura Finetek, Tokyo, Japan). OCT-embedded sections were stained with antibodies for fluorescence microscopic analysis. Each section was incubated with the primary antibodies (Abs) for 24 hours at 4 degrees and with the secondary Abs for 1 hour at room temperature (see key resources table). Following a wash step, sections were mounted with Vibrance Antifade Mounting Medium with DAPI (Vector LABORATORIES, Newark, CA; H-1800), and observed under confocal laser scanning microscopy (Carl Zeiss, Oberkochen, Baden-Württemberg, Germany).

STATISTICAL ANALYSIS AND QUANTIFICATION

Alignment, species deconvolution, and clustering

All fastq files were aligned to the mouse (GRCm39, from ENSEMBL v109), rat (mRatBN7.2, from ENSEMBL v109), and a concatenated mouse and rat genome using CellRanger v7.1.0.[59] For mapping, we retained protein-coding genes, lncRNA, antisense transcripts, snoRNA, snRNA, miRNA, and scaRNA. The resulting count matrices were read into scanpy v1.9.5 and the proportion of aligned reads to each genome in the concatenated genome was used to determine the species of origin of each cell.[51] A cell was assigned to mouse if greater than or equal to 70% of aligned reads aligned to the mouse genome, assigned to rat if greater than or equal to 70% of aligned reads aligned to the rat genome, and discarded as a doublet otherwise. For all subsequent steps, we only used the mouse-aligned-only counts for mouse cells and the rat-aligned-only counts for rat cells. We then removed all genes on sex chromosomes, restricted to mouse-rat one-to-one orthologs (defined using data from ENSEMBL), and performed standard pre-processing steps to remove low quality cells.[59] For the latter, we performed standard filtering and removed cells with greater than 15% of counts coming from mitochondrial reads and cells with n_genes_by_counts (a commonly used quality control metric) greater than 7500. Next, we converted to the log normalized counts, identified highly variable genes, computed principal components, used the Python implementation of harmony v0.0.9 to integrate the mouse and rat cells, and then found nearest neighbors.[52] We then clustered the cells using the Leiden algorithm v0.10.2 with resolution equal to 0.1.[53] This first round of clustering split cells into neuronal, connective tissue, and hematopoietic lineages. The neuronal and connective tissue lineage cells were then retained for further analysis (see below).

Several libraries did not contain a sufficient number of cells to be useful for our analysis. Therefore, we used the rat-like chimera data from library MR1 for both neural and connective tissue analyses, the mouse-like chimera data from library RM1 for neural analyses, and the mouse-like chimera data from library RM2 for all subsequent analyses after the pseudobulking step (see below).

Our main goal in clustering the cells was to identify cell types and remove cell subtypes that had fewer than 10 cells in any of donor mouse cells, donor rat cells, host mouse cells, or host rat cells in MR1 and RM1 (for neuronal lineages) or MR1 and RM2 (for connective tissue). We repeated the harmony and Leiden-based procedure (with resolution equal to 0.5) on the neural lineages and identified 18 subclusters. Due to a low number of cells in other cell types, we restricted further analysis to forebrain glutamatergic progenitors (*Gli3* high, *Eomes*-, *Mki67*+), forebrain GABAergic progenitors (*Gli3* low, *Eomes*-, *Mki67*+), intermediate progenitors (*Eomes*+, *Mki67*+), forebrain glutamatergic neurons (*Slc17a6*+, *Tcf7l2*-, *Reln*-, *Hoxb8*-, *Mki67*-), forebrain GABAergic neurons (*Gad1*+, *Hoxb8*-), spinal glutamatergic neurons (*Slc17a6*+, *Hoxb8*+), and spinal GABAergic neurons (*Gad1*+, *Hoxb8*+).[60–62] As some forebrain GABAergic subtypes had very few rat donor cells, we subclustered (Leiden resolution equal to 0.35) that cell type further, removed the subtypes with very few rat donor cells, and then merged the remaining 3 cell subtypes which had similar cell type proportions across the four species-environment combinations.

For the connective tissue, we reclustered the cells (Leiden resolution equal to 0.15) and identified six clusters. We focused subsequent analysis on mesenchymal cells (*Postn* high, *Prrx1* high) and chondrocytes (*Acan*+).[63] We subclustered both cell types (Leiden resolution equal to 0.25 for mesenchymal cells and chondrocytes) and removed subtypes according to the above criteria (i.e. at least 10 cells in each of the 4 species-environment combinations). After this procedure, we retained one chondrocyte subcluster, two mesenchymal subclusters, and cycling mesenchymal cells (*Mki67*+) for further analysis.

Gene filtering, pseudobulking, and count normalization

To avoid overly noisy estimates of intrinsic and extrinsic divergence, we removed genes that were not expressed in greater than or equal to 20% of cells in at least one of the four species-environment combinations. For each gene, we summed all counts across cells within a cell type in each library for mouse and rat cells separately and removed genes that had fewer than ten counts in all four species-environment combinations.

To normalize counts, we divided by total expression and multiplied by 10,000 for each pseudobulked sample in each cell type separately. Rather than normalize the counts directly, we first randomly sampled from the gene-count distribution within each sample in each cell type so that the total number of counts was identical across all species-environment combinations. We performed this procedure 100 times and took the mean normalized counts across all 100 samplings. This mean value was highly correlated with the actual normalized counts (Spearman's rho > 0.999, $p < 10^{-300}$). This procedure was used instead of simply normalizing by total counts because this can lead to biased estimation of log fold-changes when there are few counts in one of the four species-environment combinations. For example, consider a scenario in which donor mouse, host mouse, and host rat all have 1,000,000 total counts whereas donor rat only has 100,000 counts. If a gene has 99 (100 after adding a pseudocount) counts in donor mouse and host mouse and 0 (1 after adding a pseudocount) counts for donor rat and host rat and we normalize to total counts and multiply by 10,000, we will have 1 normalized count in donor mouse and host mouse, 0.01 normalized counts in host rat, and 0.1 normalized counts in donor rat. If we then compute fold-changes, donor mouse divided by host rat would be 100 but donor mouse divided by donor rat would be 10. This difference would propagate and (see below) cause overestimation of the extrinsic or interaction component of gene expression and underestimation of the intrinsic component. Versions of this example frequently occur in practice due to considerable differences in the number of cells profiled between HR, HM, DR, and DM for some cell types. For example, we sampled relatively few GABAergic neurons in DR compared to HR, HM, and DM.

We computed the per-cell normalized counts shown in the supplemental figures using the default log normalization used by scanpy. This involves normalizing to counts per 10,000 (as was done for the pseudobulked expression) and then taking the natural logarithm of this value plus a pseudocount.

We refer to normalized counts for mouse cells in a rat-like environment as DM (donor mouse), counts for rat cells in a rat-like environment as HR (host rat), counts for mouse cells in a mouse-like environment as HM (host mouse), and counts for rat cells in a mouse-like environment as DR (donor rat).

A framework to decompose gene expression divergence into extrinsic, intrinsic, and interaction components

Throughout, we use the $\log_2$ fold-change to measure gene expression divergence. Our goal is to decompose the estimate of the overall divergence in gene expression (i.e. the $\log_2$ fold-change between HR and HM) into the intrinsic, extrinsic, and potentially other terms (see below). To do this, we formulate the total expression of a single gene in one of the species-environment combinations as the product of an extrinsic component and an intrinsic component. For example, the expression of a gene in a donor rat cell in a mouse-like environment (D_R) is equal to the intrinsic rat expression (I_R) multiplied by the expression induced by the mouse-like environment (E_M). Throughout, the subscript numbers reflect noise and intrinsic-extrinsic interactions make the values derived from different chimeras and cells unequal. In this framework, the normalized counts are:

$$H_M = I_{M1} \times E_{M1}$$

$$H_R = I_{R1} \times E_{R1}$$

$$D_M = I_{M2} \times E_{R2}$$
$$D_R = I_{R2} \times E_{M2}$$

Where H_M is the gene expression for a host mouse cell in a mouse-like environment, H_R is the gene expression for a host rat cell in a rat-like environment, D_M is the gene expression for a donor mouse cell in a rat-like environment, and D_R is the gene expression for a donor rat cell in a mouse-like environment. Using these four values with three degrees of freedom, we can obtain two independent estimates of the intrinsic divergence between mouse and rat (I_1 and I_2):

$$I_1 = log_2\left(\frac{H_R}{D_M}\right) = log_2\left(\frac{I_{R1} \times E_{R1}}{I_{M2} \times E_{R2}}\right) \approx log_2\left(\frac{I_{R1}}{I_{M2}}\right)$$
$$I_2 = log_2\left(\frac{D_R}{H_M}\right) = log_2\left(\frac{I_{R2} \times E_{M2}}{I_{M1} \times E_{M1}}\right) \approx log_2\left(\frac{I_{R2}}{I_{M1}}\right)$$

Similarly, we obtain two independent estimates of the extrinsic divergence between mouse and rat (E_1 and E_2):

$$E_1 = log_2\left(\frac{D_M}{H_M}\right) = log_2\left(\frac{I_{M2} \times E_{R2}}{I_{M1} \times E_{M1}}\right) = log_2\left(\frac{E_{R2}}{E_{M1}}\right)$$

$$E_2 = log_2\left(\frac{H_R}{D_R}\right) = log_2\left(\frac{I_{R1} \times E_{R1}}{I_{R2} \times E_{M2}}\right) = log_2\left(\frac{E_{R1}}{E_{M2}}\right)$$

However, for some genes the effects of a rat-like environment on mouse cells will likely differ from the effects of a rat-like environment on rat cells (and vice versa) due to interactions between extrinsic and intrinsic divergence. In the absence of such an interaction, the two estimates of intrinsic divergence should be equal and the two estimates of extrinsic divergence should be equal, so the difference between these pairs of estimates is itself an estimate of the interaction between extrinsic and intrinsic divergence. Therefore, to quantify this interaction, we can compute:

$$I_1 - I_2 = log_2\left(\frac{H_R}{D_M}\right) - log_2\left(\frac{D_R}{H_M}\right) = log_2\left(\frac{H_R \times H_M}{D_M \times D_R}\right) = 2N$$

This value is identical to what we obtain starting with extrinsic divergence:

$$E_2 - E_1 = log_2\left(\frac{H_R}{D_R}\right) - log_2\left(\frac{D_M}{H_M}\right) = log_2\left(\frac{H_R \times H_M}{D_M \times D_R}\right) = 2N$$

The factor of 2 is necessary because we are subtracting (which is equivalent to adding if you switch the numerator and denominator of the second estimate) two extrinsic estimates, so without the factor of 2 there would be twice as much weight placed on the interaction divergence compared to the intrinsic or extrinsic divergence.

Based on this framework, we obtain our final estimates of intrinsic, extrinsic, and interaction divergence by averaging the two estimates:

$$E = \frac{E_2+E_1}{2}$$

$$I = \frac{I_1+I_2}{2}$$

$$N = \frac{2N+2N}{4}$$

To compute the proportion extrinsic, intrinsic, and interaction divergence, we take the magnitude of each quantity and divide it by the sum of the magnitudes of all three quantities:

$$I_p = \frac{|I|}{|I|+|E|+|N|}$$

$$E_p = \frac{|E|}{|I|+|E|+|N|}$$

$$N_p = \frac{|N|}{|I|+|E|+|N|}$$

Finally, the signed proportion extrinsic, intrinsic, and interaction divergence is then computed as the sign of the divergence multiplied by the proportion divergence for each quantity. We only applied this framework to genes that had some evidence for divergence in expression level (i.e. an absolute log_2 fold-change greater than 0.5 in at least one of the four comparisons).

Data processing and analysis for predictors of intrinsic and extrinsic divergence

We tested six variables for their correlation with [intrinsic proportion - extrinsic proportion]. In general, if high quality data were not available for both rats and mice, we used data from the human genome to avoid biasing our analysis. To measure gene expression divergence and total expression in mice and rats, we used the log_2 fold-change between and average expression of mouse cells in a mouse environment and rat cells in a rat environment. To measure evolutionary constraint, we used the metric for constraint on each gene published in Zeng et al.[49] As a proxy for regulatory complexity, we assigned candidate *cis*-regulatory elements (CREs) from the ENCODE project to the nearest TSS of a protein-coding gene, removed CREs greater than 100,000 bases from the nearest TSS of a protein coding gene, and computed the total number of regulatory elements assigned to each gene.[50] For cell type specificity, we used total expression (as described above) for each of the eleven cell types in our analysis as input to compute Tau, a well-established measure of tissue/cell type-specificity. Tau is equal to one if a gene is completely specific to a single cell type or tissue, and zero if it is equally expressed across all cell types or tissues.[64] To compute tissue specificity in mice and rats at E13.5 and E15.25 respectively, we averaged normalized counts across replicates for six of the seven tissues from Cardoso-Moreira et al. (we excluded cerebellum/hindbrain due to its transcriptional similarity to the rest of the brain) and computed Tau.[48] Finally, we computed the log_2 fold-change

between mouse and rat within each tissue (again using E13.5 and E15.25 respectively and excluding cerebellum/hindbrain) and used the variance of the $\log_2$ fold-change across tissues as a measure of the tissue-specificity of gene expression divergence.

To merge these six variables into one matrix we converted mouse gene names to human gene names using the orthologs from the Ensembl database and joined all the above metrics together, removing any genes for which one or more of these metrics could not be computed.[59] We then calculated the Spearman correlation between [intrinsic proportion - extrinsic proportion] and each of these variables.[65]

Enrichment analysis

For the enrichment analysis, we either performed analysis on each cell type separately or averaged expression in connective tissue, the neural lineage (i.e. including spinal neurons with the forebrain cells), or across all eleven cell types. In the first two cases, we required that a gene have passed our filtering criteria (see above) in at least three cell types and in the latter case we required a gene to have passed the filtering criteria in at least five cell types. Throughout, we always use GSEAPY preranked with parameters: threads=4, permutation_num=1000, format='png', seed=6, min_size = 10, max_size = 300.[27] To identify enriched gene sets in extrinsically driven genes, we sorted the input gene list by the signed extrinsic proportion, converted mouse gene symbols to human gene symbols using orthologs defined by Ensembl, and used GSEAPY preranked.[27,59] We tested gene sets from the gene ontology (GO) biological process (2023 version), GO cellular component (2023 version), GO molecular function (2023 version), MGI Level 4 Mammalian Phenotypes (2021 version), and the CORUM protein complex database.[66–68] In all cases, we used the versions directly supported by GSEAPY. After running on all cell types, we retained all terms across the five ontologies with FDR < 0.25.

To identify shared and cell type-specific enrichments, we counted the number of times each term occurred in this final merged file. To further investigate shared terms, we visualized the signed extrinsic and signed intrinsic proportion as shown in Figures 5D–5F. The $\log_2$ fold-enrichment (shown in many figure panels) is defined as the $\log_2$ fold-change for the number of genes in the gene set with signed proportion extrinsic above the rank cutoff determined by GSEAPY preranked and the number of genes below an identical cutoff (or vice versa if the enrichment was for negative proportion extrinsic divergence). For example, consider an enrichment for positive extrinsic proportion with 500 total genes above the GSEAPY cutoff, 15 of which are imprinted genes. We would then count all imprinted genes in the 500 genes with the most negative signed extrinsic proportion. If, continuing the example, 0 genes of those 500 were in the gene set, the final $\log_2$ fold-change would be:

$$log_2\left(\frac{15+1}{0+1}\right) = 4$$

Transcription factor target enrichment analysis

For the transcription factor (TF) target enrichment analysis, we focused on the averaged signed extrinsic and intrinsic proportion across all cell types to reduce noise, but also performed the analysis on each cell type separately. The analysis was performed as described above, but using the TF Perturbations Followed By Expression database in GSEAPY/MSigDB.[27] We then computed the Spearman correlation between the extrinsic TF target enrichment score for genes with increased expression following *Xbp1* overexpression (from GSEAPY as described above) and the signed proportion extrinsic for *Xbp1.* With regard to the significant enrichment for targets *Nfe2l1* (which codes for the protein Nrf1) for positive intrinsic divergence, the gene is often erroneously referred to as *NRF1* in the database of TF targets and so the significant enrichment for *NRF1* we observe actually refers to *Nfe2l1* (and is referred to as *Nfe2l1*) throughout the text. We repeated this analysis using the TF target enrichment score and signed proportion extrinsic proportion for *Nfe2l2* and *Stat3*. We computed a similar correlation using *Xbp1* and *Nrf1* intrinsic signed proportion and the intrinsic TF target enrichment score for *Xbp1* and *Nrf1* target genes respectively. For *Xbp1*, we also computed the correlation between the signed extrinsic or intrinsic proportion and the enrichment score for the Response to Endoplasmic Reticulum Stress (Response to ER stress) Gene Ontology category.[68] Importantly, a strong correlation between signed extrinsic divergence in *Xbp1* expression and enrichment for its targets or ER stress genes does not necessarily imply a strong correlation between signed intrinsic divergence in *Xbp1* expression and the corresponding enrichments. For example, if *cis*-acting mutations altered the expression of many *Xbp1* target genes, this might lead to weak or no correlation for signed intrinsic divergence even if there were a strong correlation for signed extrinsic divergence.

Quantification and analysis of immunofluorescence (IF) images

All quantification of fluorescence intensity was performed in Python and all IF images shown in this work were made in ImageJ.[55] We paired chimeras that were generated, fixed, processed, stained, and imaged in the same batch with the same reagents. For all regions of interest, selection, tuning, and parameter selection was performed using only the DAPI and TdTomato channels before measuring fluorescence intensity for c-Jun or Hspa5, ensuring that we were effectively blind to the outcome of the analysis and that the results were not biased.

To quantify fluorescence intensity, we read in the images and drew a rectangular region of interest containing only the medial and lateral ganglionic eminences for chimera pair 1, and only the medial ganglionic eminence for chimera 3 (as the lateral ganglionic eminence had no mouse cells in the rat-like chimera). We then used a Gaussian filter to blur the DAPI and TdTomato channels and masked the images into DAPI positive, DAPI negative, TdTomato positive, and TdTomato negative. We tuned the Gaussian

blue sigma parameter and cutoff for the masking by hand until the DAPI mask included all of the area in the region of interest except clearly visible larger regions without DAPI+ nuclei in the region of interest. For the TdTomato signal, we ensured that the mask closely matched the original TdTomato signal in the region of interest. The sigma and masking cutoff values selected in this process were used for all results presented in the text.

We then considered all DAPI+ TdTomato+ pixels to be from donor cells (species-mismatched environment) and all DAPI+ TdT- pixels to be from host cells (species-matched environment), restricted to pixels with non-zero c-Jun or Hspa5 intensity values, and computed the average fluorescent intensity in each set of pixels. For within chimera comparisons, we computed the $\log_2$ fold-change between the average donor signal and the average host signal as was done for the RNA-seq measurements. We also subtracted the average background signal from a rectangular region with no nuclei from the values computed above before computing $\log_2$ fold-changes between chimeras. As the sigma parameter and cutoff for the masking were chosen by hand, we tested all possible combinations of three different masking cutoffs (the originally selected cutoff and two other similar values) for DAPI and TdTomato for the rat-like and mouse-like chimeras separately (81 combinations in total) and obtained highly similar log fold-changes (the largest standard error for any log fold-change was 0.0046). These $\log_2$ fold-changes were then used to compute the extrinsic, intrinsic, and interaction proportions for protein expression divergence as described above for the gene expression divergence measured through scRNA-seq.

To produce the images in the publication the images were first read into ImageJ. Next, channels were assigned colors and the brightness and contrast tuned to visualize the fluorescent signal. Scale bars were then added by synchronizing the windows, drawing a horizontal line, and adding a scale bar to each image with the Scale Bar tool. These images were then exported as .png objects.

Analysis of neuron-progenitor ratios

For the forebrain glutamatergic cells, forebrain GABAergic cells, and the combined spinal cord glutamatergic and GABAergic cells (combined due to the low number of progenitors we detect), we computed the ratio of post-mitotic cells to progenitors (excluding intermediate progenitors) in all four species-environment combinations. We then used these values to compute $\log_2$ fold-changes and the intrinsic, extrinsic, and interaction proportions in the same way we did for the normalized counts from the scRNA-seq data.

Analysis of imprinted gene expression

The list of imprinted genes for mice and humans was downloaded from https://www.geneimprint.com/site/home through the "gene by species" tab. To test whether imprinted genes were enriched for high absolute interaction divergence, we created an imprinted gene set consisting of all "confident" and "predicted" imprinted genes in mice from the above resource. Only two genes were predicted and not confident, so our results are unaltered by restricting only to confident imprinted genes. We next performed enrichment analysis as described above, including averaging across cell types within nervous and connective tissue, with the following modification. Initially, we simply ranked genes by the absolute interaction divergence and observed very strong enrichment. However, due to the GSEA running sum statistic being initially developed for signed metrics (e.g. log fold-change) we reasoned that this procedure could lead to spuriously strong enrichments.[27] To address this, we rank transformed the list such that the genes with very large absolute interaction divergence have large positive values and genes with absolute interaction divergence near zero had negative values with equally large magnitude. For example, if there were 5,000 input genes, the gene with the highest absolute interaction divergence would have a value of 2,500, the next highest 2,499, etc. The gene with the lowest absolute interaction divergence would have a value of -2,500, the next lowest -2,499, etc. Using this strategy, we still observe strong enrichment (we report the p-values obtained using this strategy in the text and Figure 7D). For the signed enrichment analysis, we ranked genes by the signed interaction divergence and used GSEAPY preranked with the imprinted gene set.

To analyze the data from chimeric rodent hearts, we downloaded the counts matrices and associated metadata (including the species of origin for each cell) from GEO: GSE236400.[39] The cell type and other metadata were shared by the authors of the study.

We then pseudobulked counts by summing across all cells within a cell type for chimera 1, chimera 2, and the wildtype rat sample separately, removed genes with fewer than twenty counts in all samples, and computed counts per million (CPM). We then took the mean of the two chimera replicates and computed the $\log_2$ fold-change of rat-like environment CPM divided by mouse-like environment CPM. This value was then used in place of interaction divergence for the enrichment analyses described above.

To analyze the data from adult parathyroid glands, we downloaded files containing TPM estimates for each mouse transcript from GEO: GSE232600. We then collapsed TPM estimates to the gene level by summing the TPM for each transcript assigned to individual genes. As each sample was generated from a very small number of cells (approximately 20), expression estimates for many genes were noisy so we used the median TPM across replicates per condition as our estimate of the expression level of that gene. We removed genes with median TPM less than 5 in all conditions (species-mismatched environment, species-matched environment, and wildtype mice). We restricted to imprinted genes as described above, normalized the expression of each gene, and applied hierarchical clustering with the default parameters in seaborn clustermap. The log fold-change was computed by comparing the median TPMs. All imprinted genes with absolute $\log_2$ fold-change > 1 between expression of species-mismatched and species-matched environment samples were plotted in Figure S29.

To analyze the data from human and macaque epiblast, we downloaded the transcripts per million (TPM) matrices for human-macaque chimeric embryos and wildtype human embryos from GEO: GSE155381 and GSE109555 respectively. We restricted to

cells annotated as epiblast for the human-macaque chimeric data and used Ensembl to identify one-to-one orthologs between crab-eating macaques and humans. For the wildtype human embryos, the raw count matrices and cell type annotations were unavailable. Therefore, we restricted to cells with greater than one TPM for *NANOG*, *SOX2*, or *POU5F1* (also known as *OCT4*) as putative epiblast cells. We then averaged TPM across all cells in each species-environment category, filtered genes that were expressed below 1 TPM in all categories, and computed the $\log_2$ fold-change of human in a macaque-like environment divided by macaque in a macaque-like environment and human in a macaque-like environment divided by human in a human-like environment after adding one pseudo-TPM to all values. We then performed the enrichment analysis using absolute $\log_2$ fold-change as described for mouse and rat heart data. Although the comparison of human data across studies undoubtedly introduces batch effects, it is very unlikely that those batch effects would specifically increase the absolute $\log_2$ fold-change of imprinted genes as a whole.

Analysis of bulk RNA-seq data and developmentally dynamic gene expression

To assess how developmentally dynamic gene expression might influence our results, we focused on the E11.5, E13.5, and E15.25 (or their closest approximation e.g. E15 for rats) timepoints collected from brain tissue in Cardoso-Moreira et al.[48] For mice and rats separately, we computed the Pearson correlation between expression level and developmental stage.[65] We then classified genes as having similar, opposite, or a shift in developmentally dynamic gene expression that goes against the global difference in maturation rate between mouse and rat (referred to as "temporal shift") according to the following criteria. We considered a gene as having a similar change over development if the product of the correlation coefficients for mice and rats was greater than 0.7 (increasing if both correlation coefficients were positive, decreasing if both correlation coefficients were negative). Similarly, we classified genes as opposite if the product of the correlation coefficients was less than -0.25. These different cutoffs were chosen such that there was a sufficient number of genes for the analysis. As there were many fewer genes with opposite expression trajectories over development, a less strict cutoff (-0.25) was used. To classify genes as having a temporal shift, we restricted to the set of genes with similar changes in expression over time and required that the absolute log fold-change between mouse and rat at both E13.5 and E15.25 be less than 0.25, consistent with the logic outlined above.

To further subdivide genes, we considered a gene as having higher expression in mouse if the $\log_2$ fold-change of mouse expression divided by rat expression was greater than 0.5 and considered a gene as having higher expression in rat if the $\log_2$ fold-change was less than -0.5. Restricting to only the genes with similar gene expression trajectories in mouse and rat (i.e. the product of the mouse and rat correlation coefficients was greater than 0.7), we classified genes as increasing in expression over time if a gene had a positive correlation coefficient in mice and rats and as decreasing over time if a gene had a negative correlation coefficient in both mice and rats (for both decreasing and increasing genes, the product would be positive.

In general, to investigate the role of developmentally dynamic gene expression in our results (see Figs. S4–S9), we defined some gene set that we would expect to be enriched for a target divergence (opposing extrinsic and intrinsic divergence, reinforcing extrinsic and intrinsic divergence, mostly extrinsic divergence, or mostly interaction divergence) under a model of purely intrinsic divergence combined with some form of developmentally dynamic gene expression. We then used GSEAPY v1.0.6 preranked to test for enrichment of that gene set at the top or bottom of the gene list when ranking by the target (signed) divergence.[27] Throughout the next section, we describe each gene set in terms of what we would expect under the purely intrinsic model outlined above. We also always use GSEAPY preranked with the following parameters: threads=4, permutation_num=1000, format='png', seed=6, min_size = 10, max_size = 30000.[27]

To define the first gene set in which we expect enrichment of both opposing and reinforcing genes, we took the union of genes with similar expression trajectories in mice and rats and genes that were classified as either higher in mouse or higher in rat. We then ranked genes by the product of proportion intrinsic and proportion extrinsic and used GSEAPY preranked to test for enrichment.[27] To further investigate genes with opposing or reinforcing extrinsic and intrinsic divergence, we ranked genes by the product of the signed extrinsic divergence and signed intrinsic divergence so that genes with a large positive value for this metric are reinforcing and genes with a large negative value are opposing. We then defined the gene set in which we expect enrichment of opposing genes as the union of genes that either (1) were increasing over time and had higher expression in mouse or (2) were decreasing over time and had higher expression in rat. Similarly, for the gene set in which we expect enrichment of reinforcing genes we took the union of genes that either (1) were decreasing over time and had higher expression in mouse or (2) were increasing over time and had higher expression in rat. We then ranked genes by the product of the signed intrinsic proportion and signed extrinsic proportion (so that genes with a very low value are opposing and genes with a very high value are reinforcing) and used GSEAPY preranked.[27]

To investigate purely extrinsic genes, we defined the gene set in which we expect enrichment of extrinsic genes as the temporal shift genes (defined above), sorted by extrinsic proportion, and used GSEAPY preranked.[27] Next, we split temporal shift genes into decreasing (expected to be enriched for negative extrinsic divergence) and increasing (expected to be enriched for positive extrinsic divergence) and input both gene sets, sorted by signed extrinsic proportion, and used GSEAPY preranked.[27]

Finally, to investigate interaction divergence genes we defined the gene set we expect to be enriched for interaction divergence as the opposing genes (defined above), sorted by proportion interaction, and used GSEAPY preranked.[27] Next, we defined genes expected to have higher expression in species-matched environments as genes with increasing expression in rat and decreasing expression in mouse and genes expected to have higher expression in species-mismatched environments as genes with increasing expression in mouse and decreasing expression in rat. We then sorted the input gene list by the signed interaction proportion and tested for enrichment using GSEAPY preranked.[27]

The spatial distribution of donor cells could lead to differences in estimates of intrinsic and extrinsic divergence between cell types

In our study, different cell types and tissues have different percentages of donor cells and different spatial distributions of donor cells. For example, rat donor cells make up a much larger portion of the developing cortex than the ganglionic eminences (Figure 1F). For rat cells surrounded by other rat cells in the developing cortex, the local environment is likely more rat-like than for rat cells surrounded by mouse cells. This might result in underestimation of the extrinsic component of gene expression divergence and overestimation of the intrinsic component for forebrain glutamatergic progenitors and forebrain glutamatergic neurons compared to forebrain GABAergic cells. While we do observe a larger intrinsic component for forebrain glutamatergic neurons compared to their GABAergic counterparts, the intrinsic and extrinsic estimates for glutamatergic and GABAergic progenitors are highly similar (Figure 2G). With the scRNA-seq analysis in this study, the spatial distribution of the donor cells in each tissue is lost so we are unable to directly test the extent to which the species of the cells adjacent to each sequenced cell affects estimates of extrinsic and intrinsic divergence. In the future, spatial transcriptomics will enable more accurate estimation of extrinsic and intrinsic divergence as well as further division of the extrinsic component of gene expression divergence into global and local effects.

Cell Genomics, Volume *5*

Supplemental information

Disentangling cell-intrinsic

and cell-extrinsic factors underlying evolution

Alexander L. Starr, Toshiya Nishimura, Kyomi J. Igarashi, Chihiro Funamoto, Hiromitsu Nakauchi, and Hunter B. Fraser

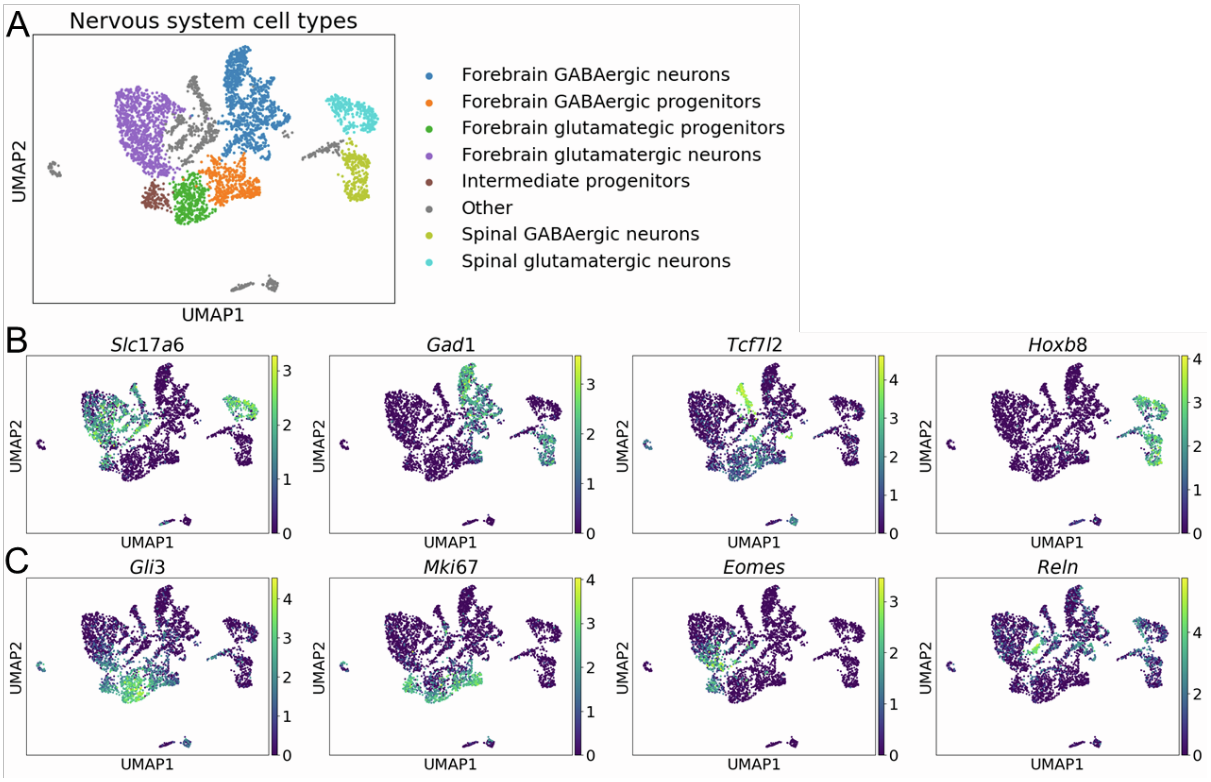

Fig. S1: Nervous system cell type annotations, related to figure 3. A) Uniform manifold approximation (UMAP) of all nervous system cell types. Cell types analyzed in this study are labeled and all other cell types are categorized as "Other". **B)** Marker genes used to classify nervous system cell types. From left to right: expression of *Slc17a6*, a marker of glutamatergic cells, expression of *Gad1,* a marker of GABAergic cells, expression of *Tcf7l2*, a marker for midbrain neurons (which were not analyzed), expression of *Hoxb8*, a marker for spinal neurons. Each point is colored by the log normalized expression in the cell represented by the point. **C)** Additional marker genes used to classify cell types. From left to right: expression of *Gli3*, which is highly expressed in forebrain glutamatergic progenitors, expression of *Mki67*, a marker of cycling cells, expression of *Eomes*, a marker for intermediate progenitors, expression of *Reln*, a marker of Cajal-Retzius cells which were excluded from further analysis.

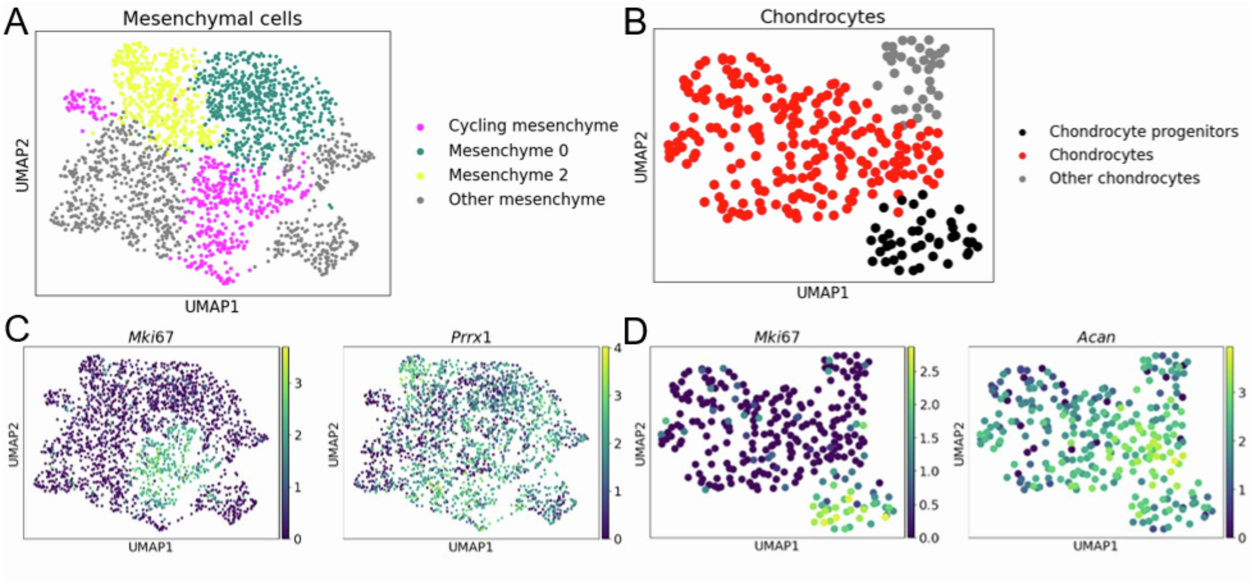

Fig. S2: Connective tissue cell type annotations, related to figure 3. A) UMAP of mesenchymal cell subtypes. UMAP is used purely for visualization and the proximity of different clusters in UMAP space cannot be used as a proxy for the true similarity between groups of cells. The small group of cycling mesenchymal cells with lower *Mki67* expression in the upper left is most similar in gene expression to the large group of cycling mesenchymal cells with higher *Mki67* expression as determined by leiden clustering and so are included with that cluster. Some cell types excluded from further analysis are labeled as Other. **B)** UMAP of chondrocyte subtypes. Cycling chondrocytes and other chondrocytes were excluded from further analysis. **C)** Expression of *Mki67*, a marker of cycling cells, and *Prrx1*, a marker of mesenchymal cells, in mesenchymal cells. Each point is colored by the log normalized expression in the cell represented by the point. **D)** Expression of *Mki67* and *Acan*, a marker of chondrocytes, in chondrocytes.

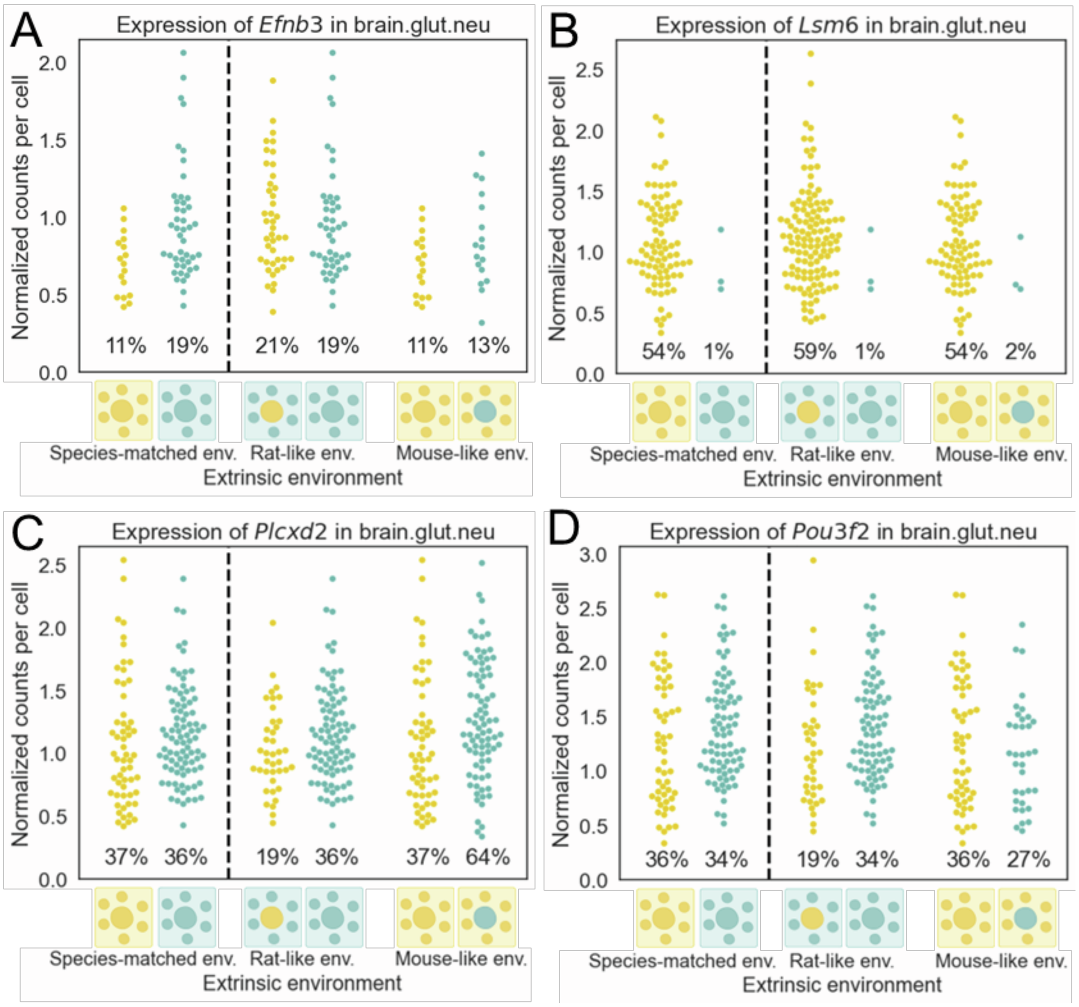

Fig. S3: Per-cell expression distribution for example genes, related to figure 3. Each swarm of points shows the normalized counts for a gene in each forebrain glutamatergic neuronal cells with non-zero counts for that gene. The percentage near the bottom of the plot indicates the percentage of cells with non-zero counts for that gene. A) Per-cell expression for *Efnb3*. B) Per-cell expression for *Lsm6*. C) Per-cell expression for *Plcxd2*. D) Per-cell expression for *Pou3f2*.

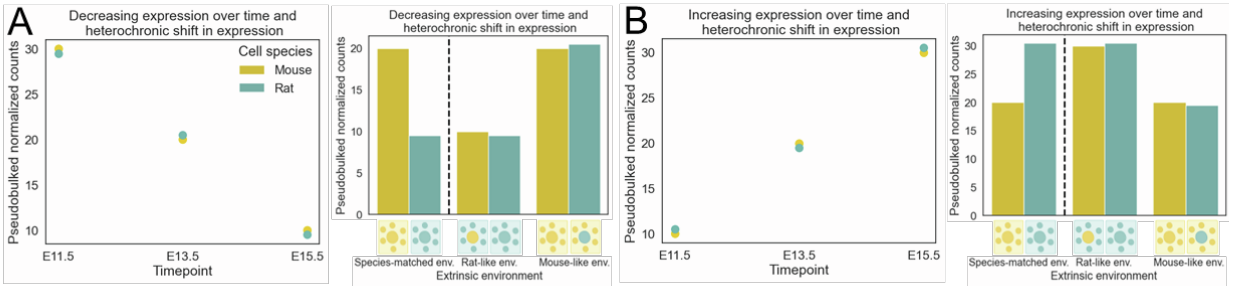

Fig. S4: Conceptual outline of the interplay of intrinsic divergence and heterochronic shifts in gene expression, related to figure 3. An important aspect of gene expression we have not explored in this study is changes in gene expression over developmental time (developmentally dynamic expression). In addition, the trajectory of gene expression can diverge between species. To explore how developmentally dynamic expression influences the results presented here, we analyzed bulk RNA-seq data from a time course of mouse and rat development, focusing on E11.5, E13.5, E15.5 in mouse and E11, E13, and E15 in rat. We analyze how a combination of intrinsic divergence and developmentally dynamic gene expression can appear as having purely interaction, purely extrinsic, or reinforcing/opposing extrinsic and intrinsic divergence in our study. First, a “temporal shift” (i.e. a shift against the global shift in gene expression associated with slower development of rats compared to mice) in gene expression could appear as extrinsic divergence in our study. As mentioned above, rat development proceeds at a slower pace than mouse development. Therefore, if there is no divergence in the expression trajectory of a gene beyond this global change, we would expect rat expression to differ from mouse expression at identical (but not stage-matched) timepoints for developmentally dynamic genes. However, the expression trajectories of some genes might not shift with this global change. This would result in the same expression trajectory (i.e. increasing or decreasing) in both species, but with similar expression levels at identical time points. **A)** Expression of a hypothetical gene across development. The gene decreases in expression over development but has very similar expression in mice and rats at the same

embryonic timepoints, going against the global difference in developmental rate between species. In the absence of extrinsic or interaction divergence, this gene would appear to have purely extrinsic divergence and higher expression in a mouse-like environment. **B)** The same as in (A) but showing a gene with increasing expression over time and a heterochronic shift. In the absence of extrinsic or interaction divergence, this gene would appear to have purely extrinsic divergence and higher expression in a rat-like environment.

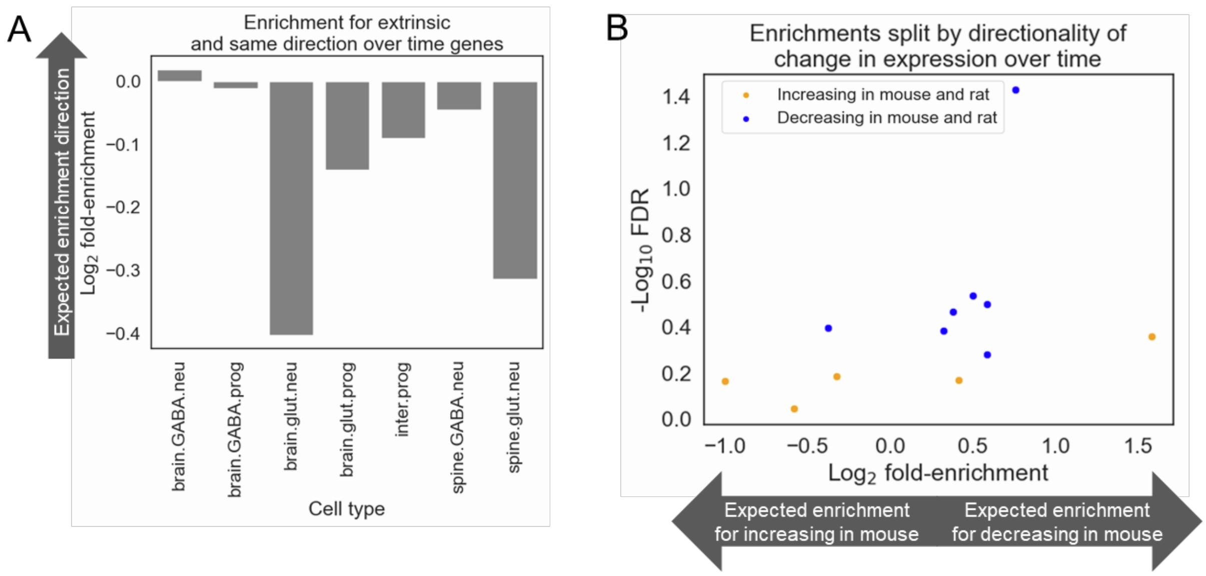

Fig. S5: Enrichment analysis for heterochronic shift genes, related to figure 3. See Methods for how heterochronic shift genes were defined. If a temporal shift occurs through purely intrinsic mechanisms, we would observe purely extrinsic divergence in our study. To test whether this occurs frequently, we identified genes with similar expression trajectories in mice and rats but small differences in expression at both E13.5 and E15.5 (absolute log fold-change less than 0.25). Importantly, when adding the restriction that the log fold-change in expression between E13.5 and E15.5 within species be greater than 0.5, less than 1% of genes fulfill these criteria suggesting the kind of temporal shift in expression hypothesized here is very rare. Therefore, we proceeded only with the restriction of low divergence between species at identical timepoints and similar expression trajectories between species. **A)** Enrichment analysis for genes with similar expression trajectories during embryonic brain development and similar expression levels at E13.5 and E15.5 in mice and rats and proportion extrinsic divergence. The y-axis shows the $\log_2$ fold-enrichment and the x-axis corresponds to cell type. The arrow shows the expected enrichment if intrinsic heterochronic shift genes were inflating estimates of extrinsic divergence. Overall, we find weak or no enrichment for genes with high proportion extrinsic divergence in this set of genes with evidence for a temporal shift (no cell types with

enrichments in the expected direction with $p < 0.1$). **B)** In addition, we would expect that temporal shift genes that decrease over time would be enriched for negative extrinsic divergence and vice versa (Fig. S4A-B). Enrichment analysis for genes with similar expression trajectories during embryonic brain development and similar expression levels at E13.5 and E15.5 in mice and rats and signed proportion extrinsic divergence. The x-axis is the $\log_2$ fold-enrichment and the y-axis is the $-\log_{10}$(FDR). The arrow shows the expected enrichment if intrinsic heterochronic shift genes were inflating estimates of extrinsic divergence. Each point corresponds to the enrichment in a central nervous system cell type and each cell type is represented twice, once as an blue dot for genes that are decreasing in mouse and rat (and have similar expression levels at E13.5 and E15.5 in mouse and rat) and once as an orange dot for genes that are increasing in mouse and rat (and have similar expression levels at E13.5 and E15.5 in mouse and rat). Here again we find only weak enrichments.

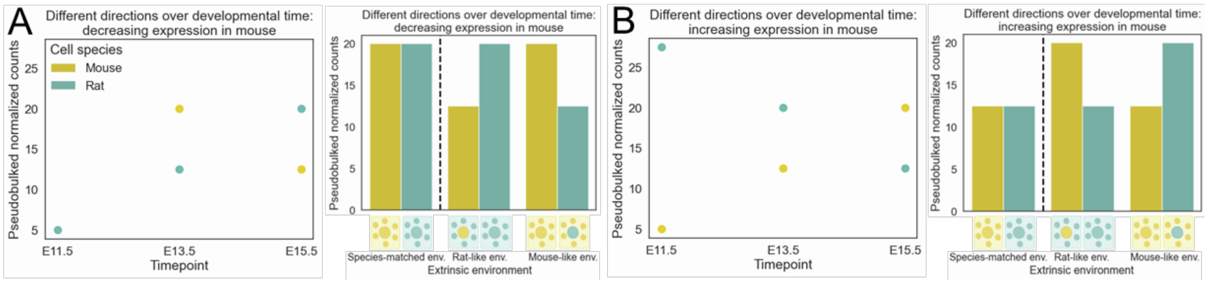

Fig. S6: Conceptual outline of the interplay of intrinsic divergence and switches in the trajectory of gene expression during development between species, related to figure 3. Another form of temporal divergence in gene expression is when the expression of a gene increases over time in one species but decreases over time in another species. For example, if a gene is intrinsically decreasing over time in mouse and increasing over time in rat (or vice versa), this could appear as purely an interaction between extrinsic and intrinsic divergence in our study. **A)** Expression of a hypothetical gene across development. The gene increases in expression over time in rats, but decreases in expression over time in mice. In the absence of extrinsic or interaction divergence, this gene would appear to have purely interaction divergence and higher expression in species-matched environments. **B)** The same as in (A) but showing a gene with increasing expression over time in mice and decreasing expression over time in rats. In the absence of extrinsic or interaction divergence, this gene would appear to have purely interaction divergence and higher expression in species-mismatched environments.

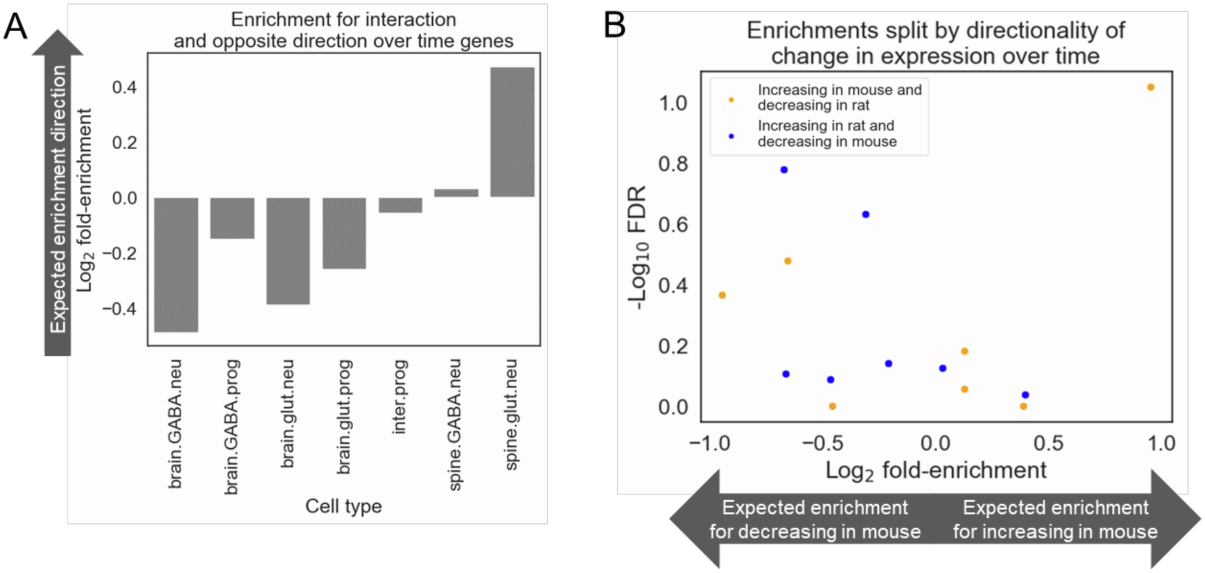

Fig. S7: Enrichment analysis for genes with switched trajectories of gene expression during development in mice and rats, related to figure 3. See Methods for how genes with switches in gene expression trajectory between species were defined. If this genes with intrinsically opposite expression trajectories contribute to the inflation of the interaction component in our study, we would expect that genes with high interaction proportions would be enriched for opposing trajectories in the time course data. **A)** Enrichment analysis for genes with switches in gene expression during embryonic brain development and proportion interaction divergence. The y-axis shows the $\log_2$ fold-enrichment and the x-axis corresponds to cell type. The arrow shows the expected enrichment if genes with the opposite gene expression trajectory between mice and rats were inflating estimates of interaction divergence. Across all brain cell types in our study, we find little to no evidence for this enrichment ($p < 0.1$ in one cell type). **B)** In addition, we can make a stronger prediction about the sign of interaction divergence if this confounder plays a major role. If gene expression is decreasing in mouse and increasing in rat, then this would lead to higher expression in species-matched environments (i.e. negative interaction divergence) due to the slower development of rat cells (Fig. S6A). As a result, this category of genes should be enriched for negative interaction divergence. Similarly, genes that

increase in expression over time in mouse but decrease over time in rat would lead to higher expression in species-mismatched environments (i.e. positive interaction divergence, Fig. S6B). This category should then be enriched for positive interaction divergence. Enrichment analysis for genes with switches in gene expression during embryonic brain development between species and signed proportion interaction divergence. The x-axis is $\log_2$ fold-enrichment and the y-axis is the $-\log_{10}$(FDR). The arrow shows the expected enrichment if genes with the opposite gene expression trajectory between mice and rats were inflating estimates of interaction divergence. Each point corresponds to the enrichment in a central nervous system cell type and each cell type is represented twice, once as a blue dot for genes that are increasing over time in rat and decreasing over time in mouse, and once as an orange dot for genes that are decreasing over time in rat and increasing over time in mouse. We find weak to no enrichment in the expected direction ($p < 0.1$ for one cell type for increasing in mouse, no cell types for decreasing in mouse).

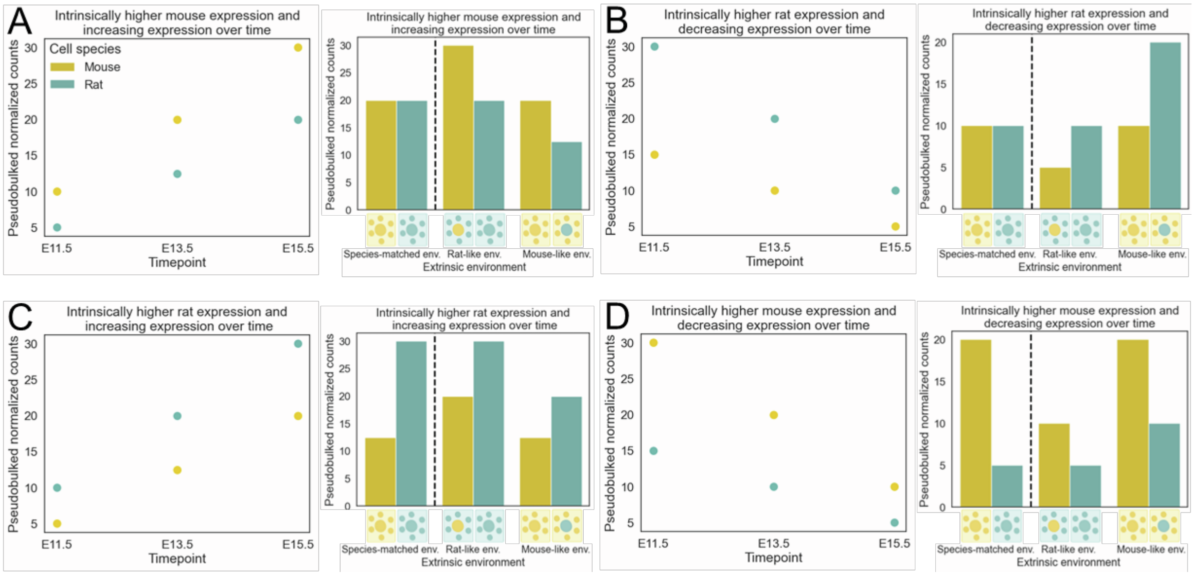

Fig. S8: Conceptual outline of the interplay of intrinsic divergence and conserved gene expression trajectories, related to figure 3. Intrinsic divergence coupled with a conserved expression trajectory can lead to the appearance of opposing or reinforcing extrinsic or intrinsic divergence. For example, if a gene is increasing in expression over time in both species and is intrinsically more highly expressed in mouse cells, this can appear as opposing extrinsic and intrinsic divergence in our study. In general, we would expect that both opposing and reinforcing genes would be enriched for genes with conserved expression trajectories in mouse and rat development. **A)** Expression of a hypothetical gene across development. The gene increases in expression over development in both mice and rats, but is intrinsically more highly expressed in mice. In the absence of extrinsic or interaction divergence, this gene would appear to have opposing extrinsic and intrinsic divergence. **B)** The same as in (A) but showing a gene with decreasing expression over time in both species and intrinsically higher expression in rat cells. In the absence of extrinsic or interaction divergence, this gene would appear to have opposing extrinsic and intrinsic divergence. **C)** The same as in (A) but showing a gene with increasing expression over time in both species and intrinsically higher expression in rat cells. In the

absence of extrinsic or interaction divergence, this gene would appear to have reinforcing extrinsic and intrinsic divergence. **D)** The same as in (A) but showing a gene with decreasing expression over time in both species and intrinsically higher expression in mouse cells. In the absence of extrinsic or interaction divergence, this gene would appear to have reinforcing extrinsic and intrinsic divergence.

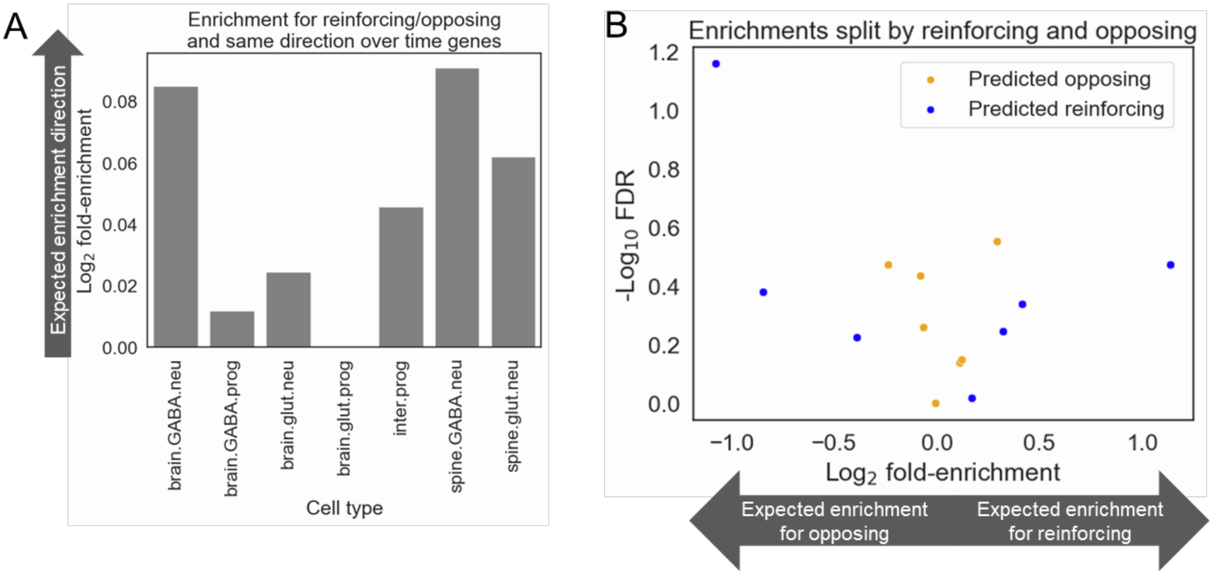

Fig. S9: Enrichment analysis for genes with conserved gene expression trajectories and reinforcing/opposing intrinsic and extrinsic divergence, related to figure 3. See Methods for how genes with conserved expression trajectories between species were defined. We can make the more specific hypothesis that genes that are intrinsically higher in mouse cells and increasing over time as well as genes that are intrinsically higher in rat and decreasing over time should be enriched for opposing extrinsic and intrinsic divergence (Fig. S8A-B). On the other hand, genes that are intrinsically higher in mouse cells and decreasing over time as well as genes that are intrinsically higher in rat and increasing over time should be enriched for reinforcing genes (Fig. S8C-D). **A)** Enrichment analysis for genes with conserved expression trajectories between species and the product of intrinsic proportion divergence and extrinsic proportion divergence. For this metric, genes with high values have opposing or reinforcing expression and genes with low values do not. The y-axis shows the $\log_2$ fold-enrichment and the y-axis corresponds to cell type. The arrow shows the expected enrichment if genes with conserved expression trajectories between mice and rats were inflating estimates of reinforcing/opposing divergence. **B)** Enrichment analysis for genes with conserved expression trajectories between species and the product of signed intrinsic proportion divergence and

signed extrinsic proportion divergence. For this metric, larger positive values indicate reinforcing intrinsic and extrinsic divergence, large negative values indicate opposing intrinsic and extrinsic divergence, and values near zero indicate neither reinforcing nor opposing intrinsic and extrinsic divergence. The x-axis is the $\log_2$ fold-enrichment and the y-axis is the $-\log_{10}$(FDR). The arrow shows the expected enrichment if genes with the conserved gene expression trajectories between mice and rats were inflating estimates of reinforcing/opposing intrinsic and extrinsic divergence. Each point corresponds to the enrichment in a central nervous system cell type and each cell type is represented twice, once as a blue dot for genes that are increasing over time in mouse and rat so would be predicted to appear as having reinforcing extrinsic and intrinsic divergence in our study, and once as an orange dot for genes that are decreasing over time in mouse and rat so would be predicted to appear as having opposing extrinsic and intrinsic divergence in our study. We find limited evidence for either hypothesis. Although this suggests that intrinsic divergence coupled with similar changes in expression over time is not the primary contributor to opposing or reinforcing extrinsic and intrinsic divergence, there are many genes with conserved expression trajectories suggesting that this area in particular should be explored further in future studies. Overall, these results suggest that a combination of intrinsic divergence and developmentally dynamic expression do not overly inflate our estimates of extrinsic and interaction divergence. However, we have only discussed intrinsic divergence in conjunction with developmentally dynamic gene expression. Various other complex combinations of divergence can instead inflate estimates of intrinsic divergence. For example, extrinsic divergence itself can be partially responsible for global shifts in the trajectory of gene expression further adding to complexity. Data from developmental time courses in reciprocal chimeras will undoubtedly provide valuable insight into how intrinsic, extrinsic, and temporal divergence interact and be vital in developing a more complete understanding of the molecular mechanisms underlying gene expression divergence.

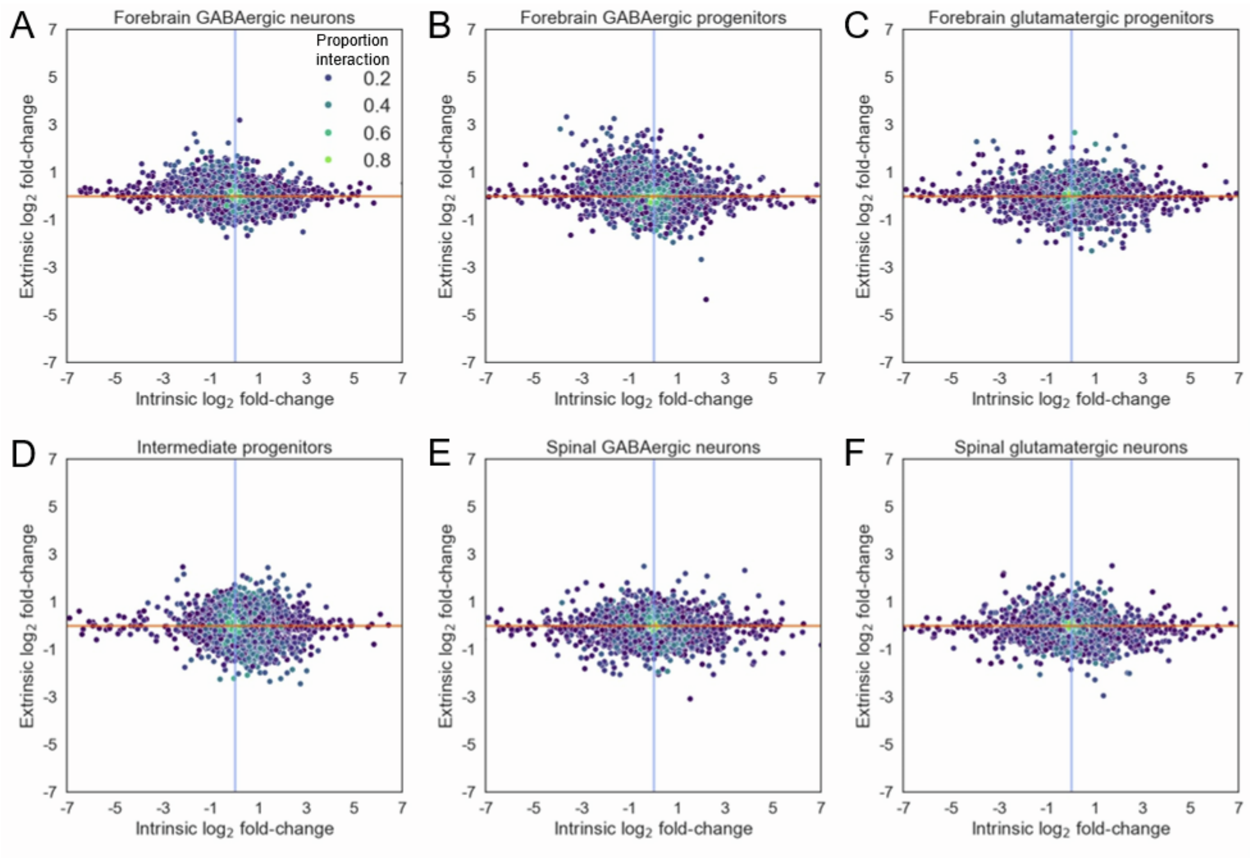

Fig. S10: Intrinsic and extrinsic divergence of nervous system cell types, related to figure 3. Each point is a gene. Intrinsic divergence is on the y-axis and extrinsic divergence is on the x-axis. Genes are colored by their proportion interaction. Forebrain glutamatergic neurons are shown in Fig. 2F. **A)** Plot for forebrain GABAergic neurons. **B)** Plot for forebrain GABAergic progenitors. **C)** Plot for forebrain glutamatergic progenitors. **D)** Plot for intermediate progenitors. **E)** Plot for spinal GABAergic neurons. **F)** Plot for spinal glutamatergic neurons.

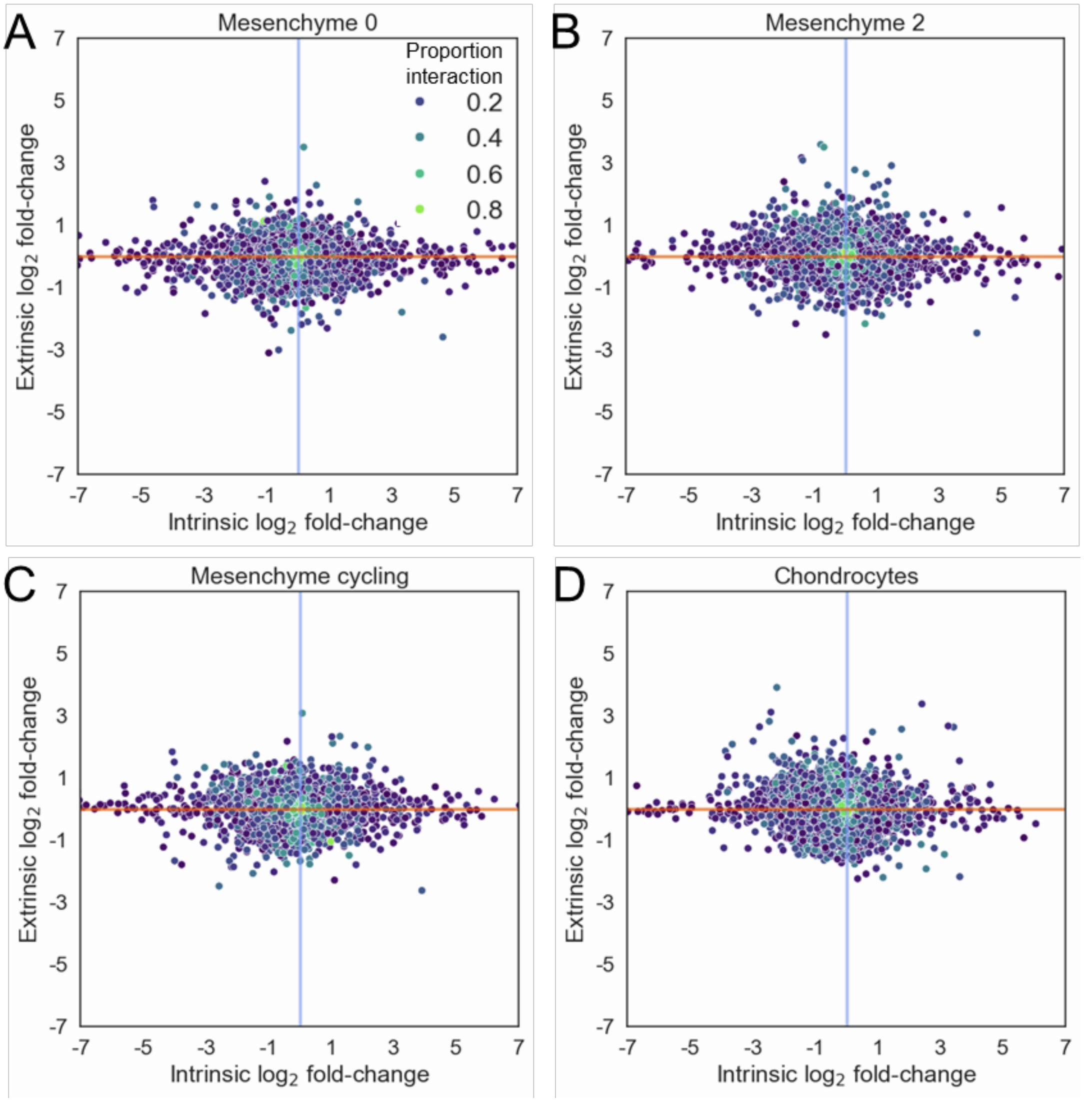

Fig. S11: Intrinsic and extrinsic divergence of connective tissue cell types, related to figure 3. Each point is a gene. Intrinsic divergence is on the y-axis and extrinsic divergence is on the x-axis. Genes are colored by their proportion interaction. **A)** Plot for mesenchyme cluster 0. **B)** Plot for mesenchyme cluster 2. **C)** Plot for cycling mesenchymal cells. **D)** Plot for chondrocytes.

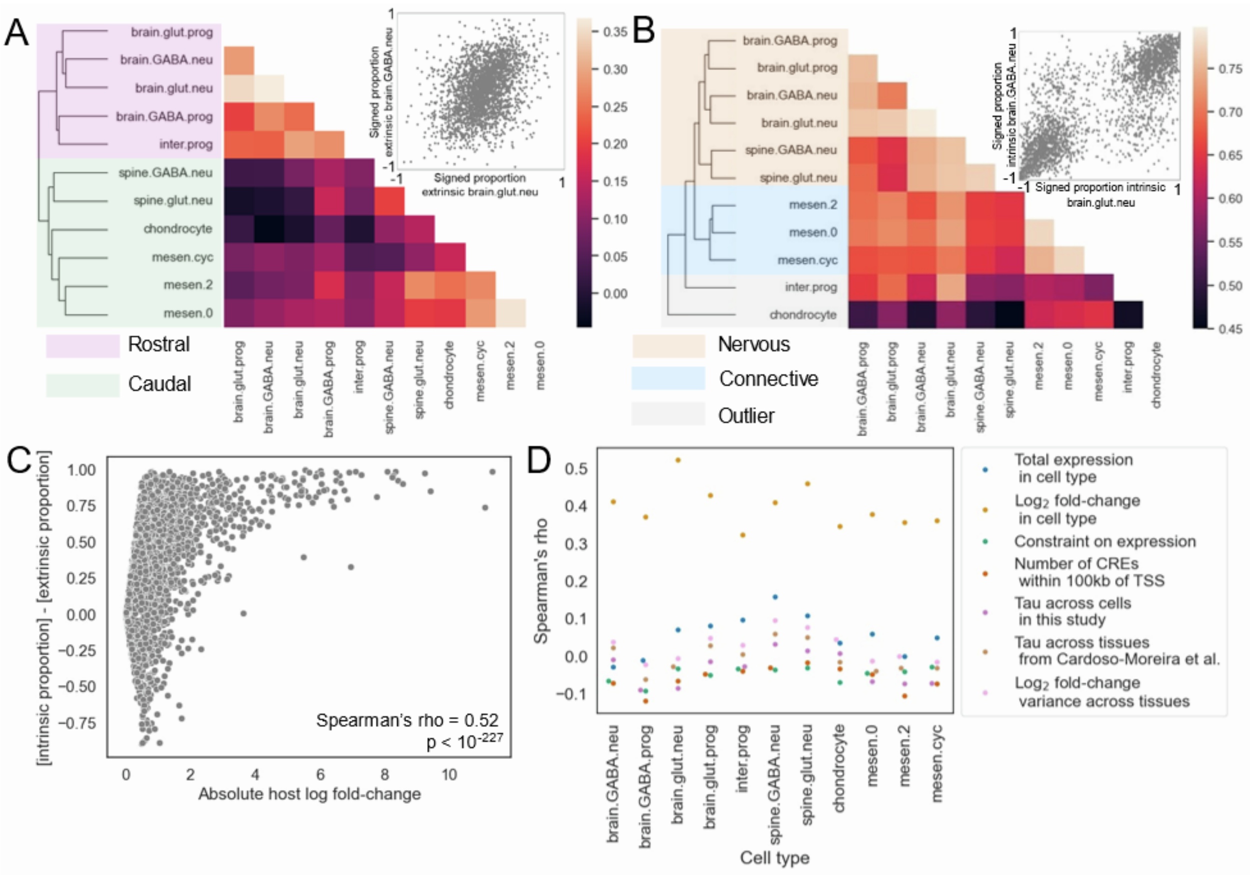

Fig. S12: Correlates of intrinsic and extrinsic divergence across cell types, related to figure 3. A) Heatmap showing Spearman correlation of signed proportion extrinsic divergence between cell types. Hierarchical clustering was performed on the Spearman rho values using the Euclidean distance metric. Cell types are shaded by their anatomical location of origin, with spinal neurons being more caudal similar to connective tissue cells. For the scatter plot in the upper right, each dot is a gene, the x-axis is the forebrain glutamatergic neuron signed proportion extrinsic divergence, and the y-axis is the forebrain GABAergic neuron signed proportion extrinsic divergence. **B)** The same as in (A) but showing the signed proportion intrinsic divergence and shading cell types by whether they cluster with nervous system cell types, connective tissue cell types, or are outliers. **C)** Scatter plot showing the relationship between absolute $\log_2$ fold-change between mouse cells in a mouse-like environment and rat cells in a rat-like environment (x-axis) and the proportion extrinsic subtracted from the proportion

intrinsic (referred to as [proportion intrinsic - proportion extrinsic], y-axis) for each gene in forebrain glutamatergic neurons. **D)** Spearman correlation coefficients for different per-gene variables and [proportion intrinsic - proportion extrinsic] across cell types.

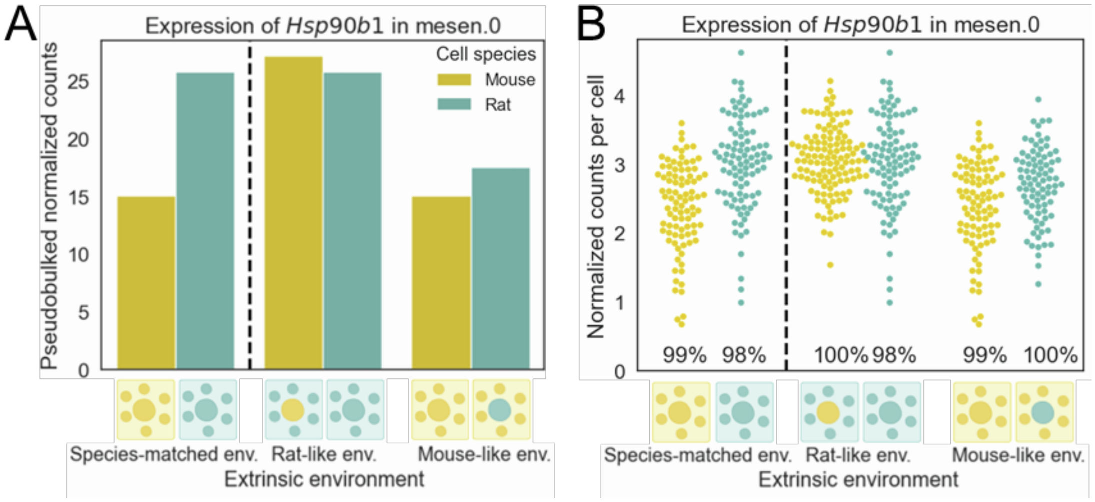

Fig. S13: Expression of *Hsp90b1*, related to figure 4. A) Expression of *Hsp90b1*, a gene involved in the ER stress response, in mesenchymal cluster 0. **B)** Per-cell expression of *Hsp90b1* in in mesenchymal cluster 0. Each swarm of points shows the normalized counts for a gene in each cell with non-zero counts for that gene. The percentage near the bottom of the plot indicates the percentage of cells with non-zero counts for that gene.

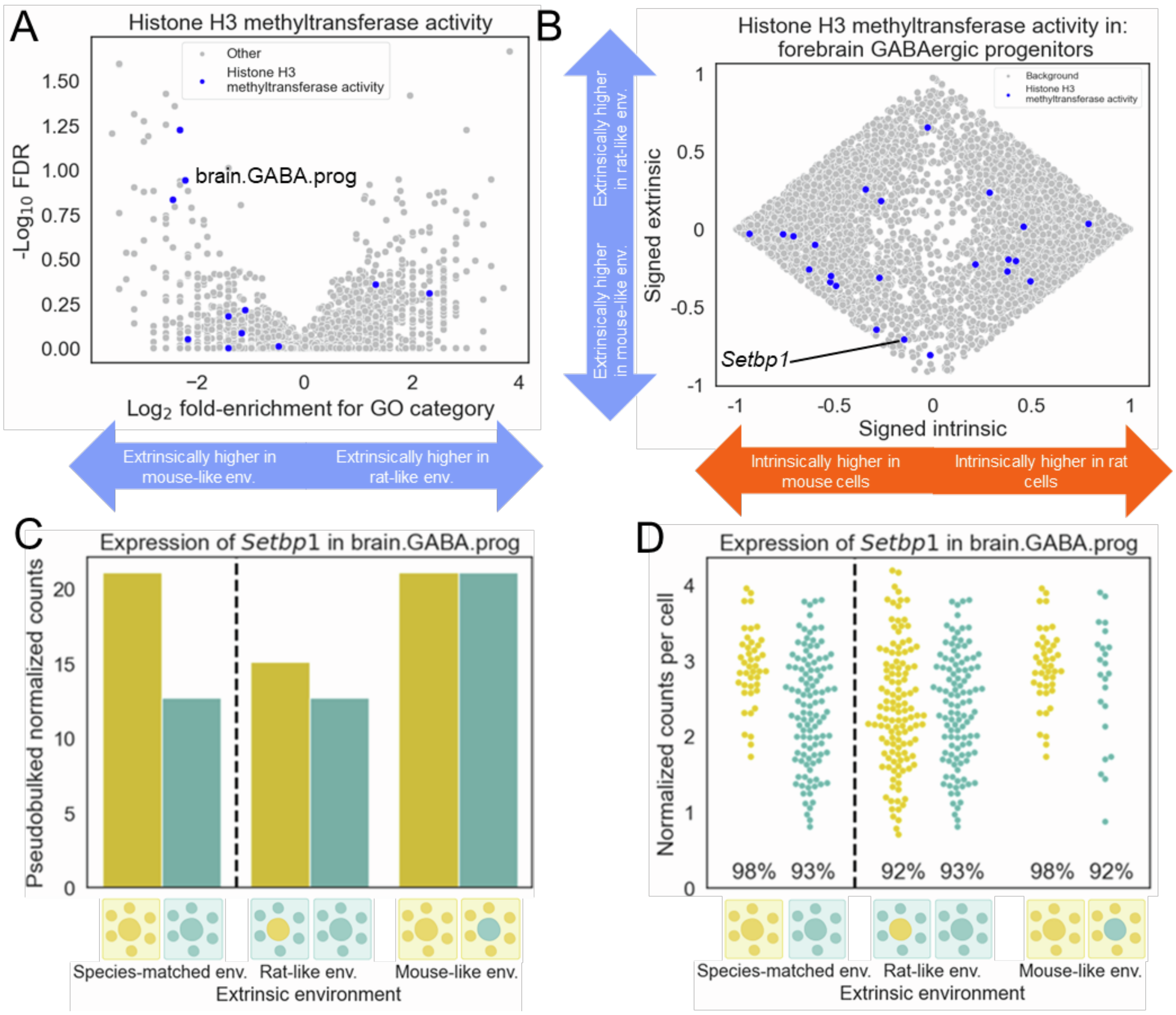

Fig. S14: Extrinsic divergence of genes encoding histone methyltransferases in central nervous system cell types, related to figure 4. A) Enrichment of genes encoding histone H3 methyltransferase for signed extrinsic divergence across cell types. Each point is a GO biological process category in a cell type and the points corresponding to the histone H3 methyltransferase activity GO category are colored blue. The x-axis shows the $\log_2$ fold-enrichment and the y-axis shows the $-\log_{10}$ false discovery rate. **B)** Scatterplot showing signed proportion intrinsic divergence (x-axis) and signed proportion extrinsic divergence (y-axis) for all genes passing our filtering criteria for forebrain GABAergic progenitors. Genes coding for histone H3 methyltransferases are shown in blue and all other genes are shown in grey. **C)**

Expression of *Setbp1*, a gene involved in histone methylation, in forebrain GABAergic progenitors. **D)** Per-cell expression *Setbp1* in forebrain GABAergic progenitors. Each swarm of points shows the normalized counts for a gene in each cell with non-zero counts for that gene. The percentage near the bottom of the plot indicates the percentage of cells with non-zero counts for that gene.

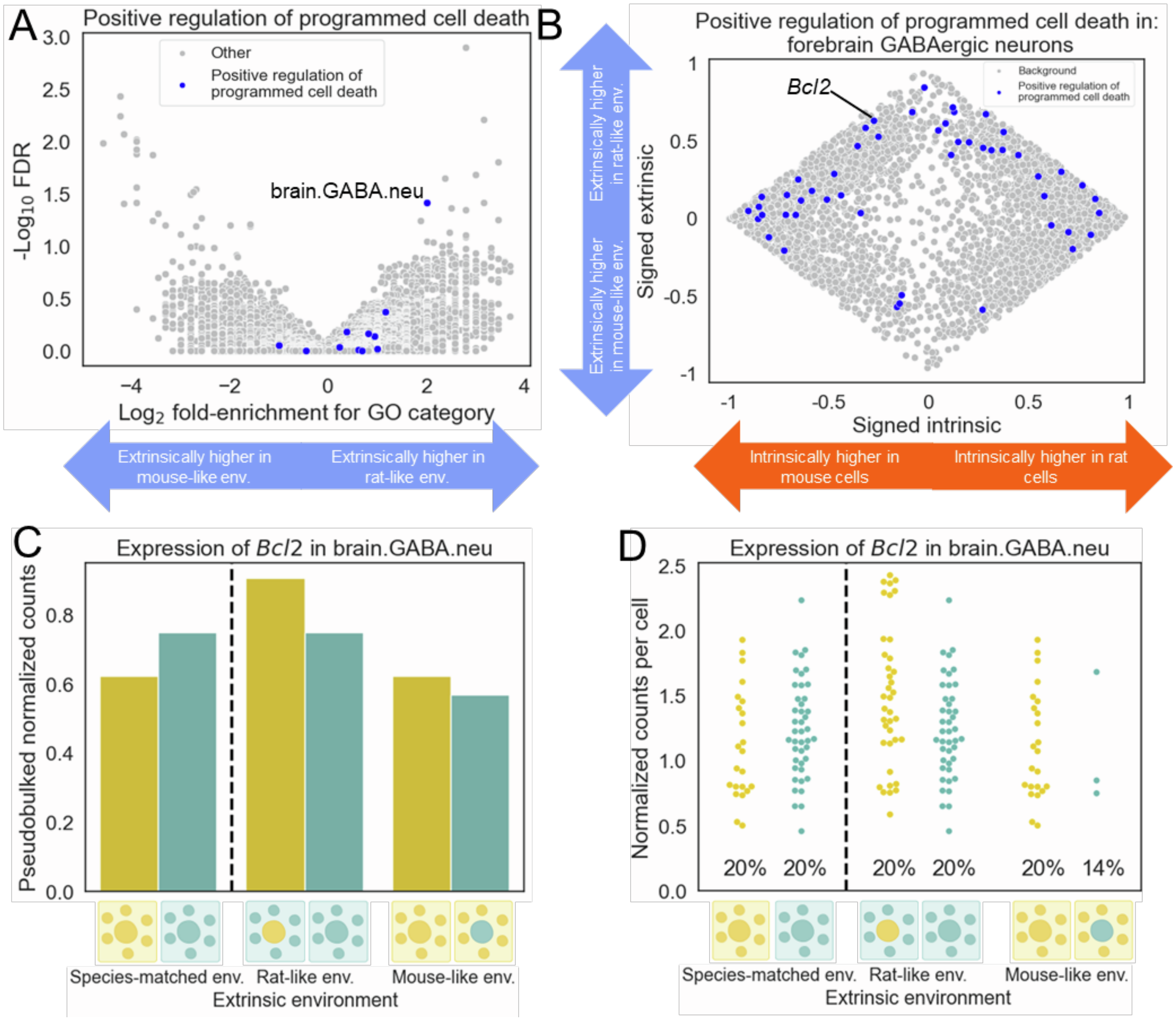

Fig. S15: Extrinsic divergence of genes involved in the positive regulation of programmed cell death in forebrain GABAergic neurons, related to figure 4. A) Enrichment of genes involved in the positive regulation of programmed cell death for signed extrinsic divergence across cell types. Each point is a GO biological process category in a cell type and the points corresponding to the positive regulation of programmed cell death GO category are colored blue. The x-axis shows the $\log_2$ fold-enrichment and the y-axis shows the $-\log_{10}$ false discovery rate. **B)** Scatterplot showing signed proportion intrinsic divergence (x-axis) and signed proportion extrinsic divergence (y-axis) for all genes passing our filtering criteria for forebrain

GABAergic neurons. Genes involved in the positive regulation of programmed cell death are shown in blue and all other genes are shown in grey. **C)** Expression of *Bcl2*, a gene involved in the regulation of programmed cell death, in forebrain GABAergic neurons. **D)** Per-cell expression *Bcl2* in forebrain GABAergic neurons. Each swarm of points shows the normalized counts for a gene in each cell with non-zero counts for that gene. The percentage near the bottom of the plot indicates the percentage of cells with non-zero counts for that gene.

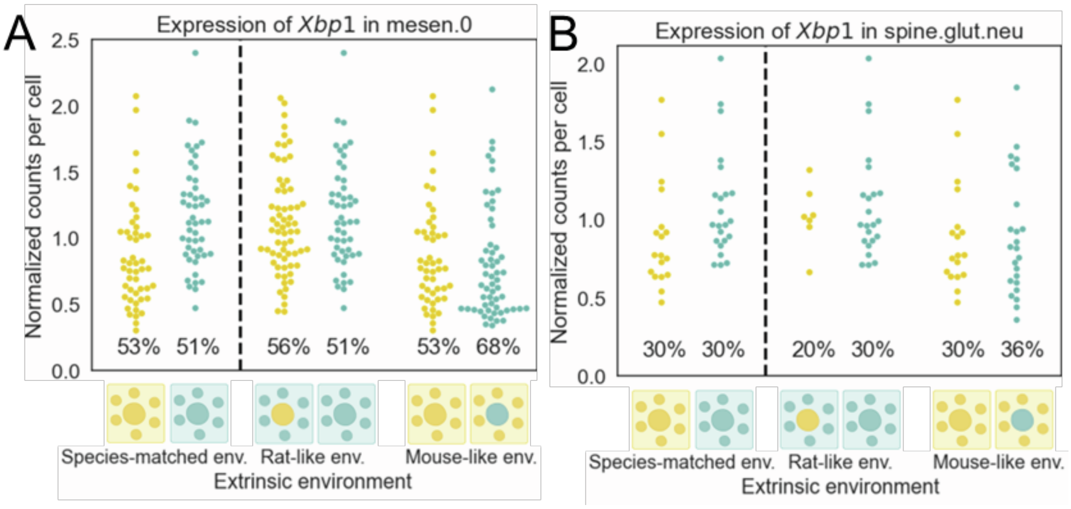

Fig. S16: Per-cell expression of *Xbp1*, related to figure 4. Each swarm of points shows the normalized counts for a gene in each cell with non-zero counts for that gene. The percentage near the bottom of the plot indicates the percentage of cells with non-zero counts for that gene. **A)** Per-cell expression *Xbp1* in mesenchymal cluster 0. **B)** Per-cell expression of *Xbp1* in spinal glutamatergic neurons.

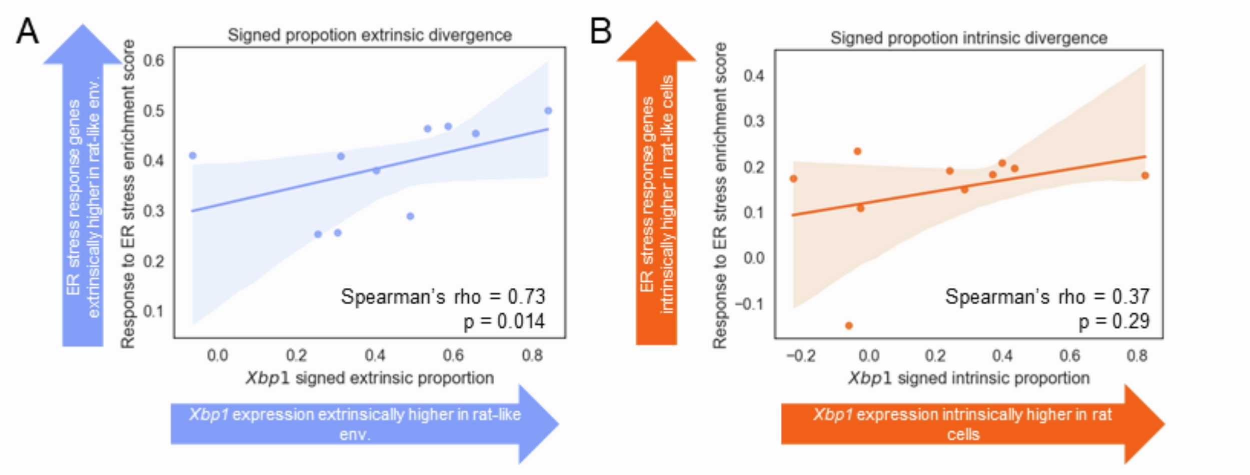

Fig. S17: Relationship between divergence in *Xbp1* expression and divergence in ER stress response gene expression, related to figure 4. A) Plot showing the relationship between signed proportion extrinsic divergence for *Xbp1* (x-axis) and the GSEA preranked enrichment score for the Response to ER stress GO category and signed proportion extrinsic divergence (y-axis). Each point is a cell type and cell types with extrinsically driven increased expression of *Xbp1* in a rat-like environment have larger values on the x-axis. Cell types with extrinsically driven increased expression of ER stress response genes in a rat-like environment have larger values on the y-axis. The line and shaded region represent the best fit and 95% confidence interval of a linear model fit to the data. **B)** Plot showing the relationship between signed proportion intrinsic divergence for *Xbp1* (x-axis) and the GSEA preranked enrichment score for the Response to ER stress GO category and signed intrinsic proportion divergence (y-axis). Each point is a cell type and cell types with intrinsically driven increased expression of *Xbp1* in rat cells have larger values on the x-axis. Cell types with intrinsically driven increased expression of ER stress response genes in rat cells have larger values on the y-axis. The line and shaded region represent the best fit and 95% confidence interval of a linear model fit to the data.

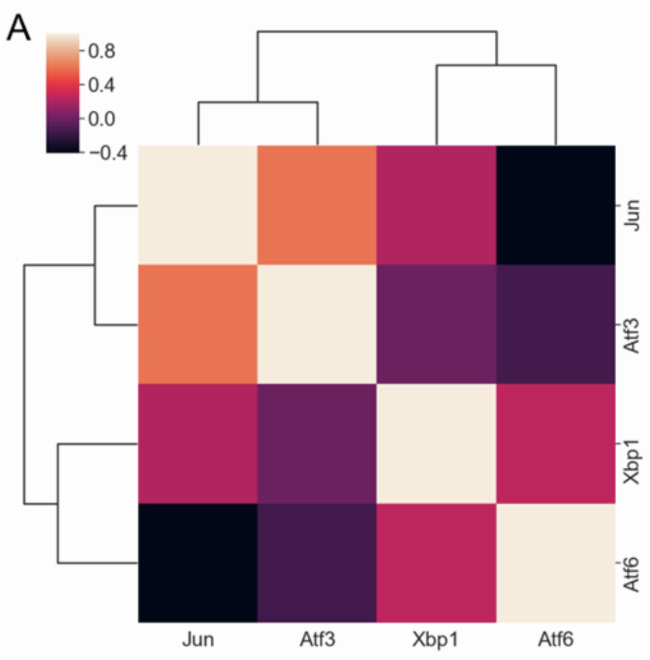

Fig. S18: Correlation between ER stress response TF signed proportion extrinsic divergence, related to figure 4. A) Heatmap showing the correlation between the signed proportion extrinsic divergence of TFs associated with the ER stress response. The genes were hierarchically clustered using the Euclidean distance metric. Only cell types in which both genes passed our filtering criteria were used to compute the correlation (see Methods).

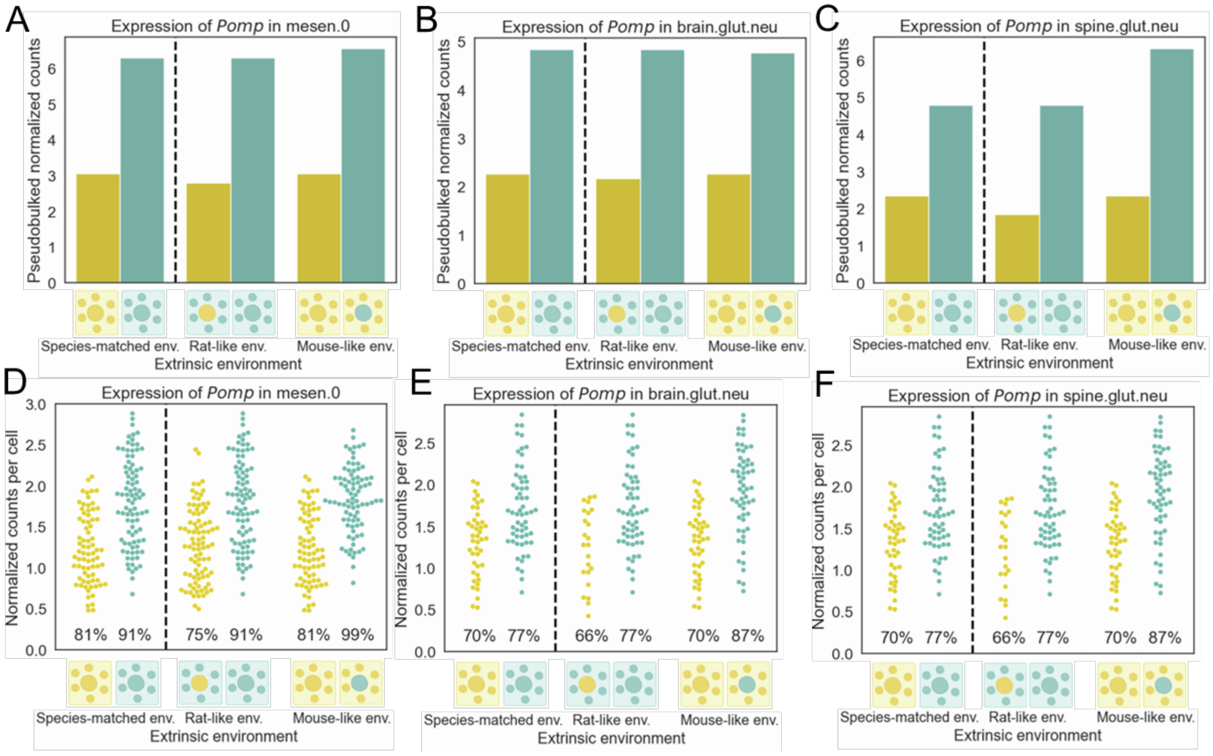

Fig. S19: Expression of *Pomp* across cell types, related to figure 5. A) Expression of the *Nfe2l1* target gene *Pomp* in mesenchymal cluster 0 cells. Expression is intrinsically higher in rat cells. **B)** Expression of *Pomp* in forebrain glutamatergic neurons. Expression is intrinsically higher in rat cells. **C)** Expression of *Pomp* in spinal glutamatergic neurons. Expression is primarily intrinsically higher in rat cells. **D)** Per-cell expression *Pomp* in mesenchymal cluster 0. Each swarm of points shows the normalized counts for a gene in each cell with non-zero counts for that gene. The percentage near the bottom of the plot indicates the percentage of cells with non-zero counts for that gene. **E)** Per-cell expression of *Pomp* in forebrain glutamatergic neurons. **F)** Per-cell expression of *Pomp* in spinal glutamatergic neurons.

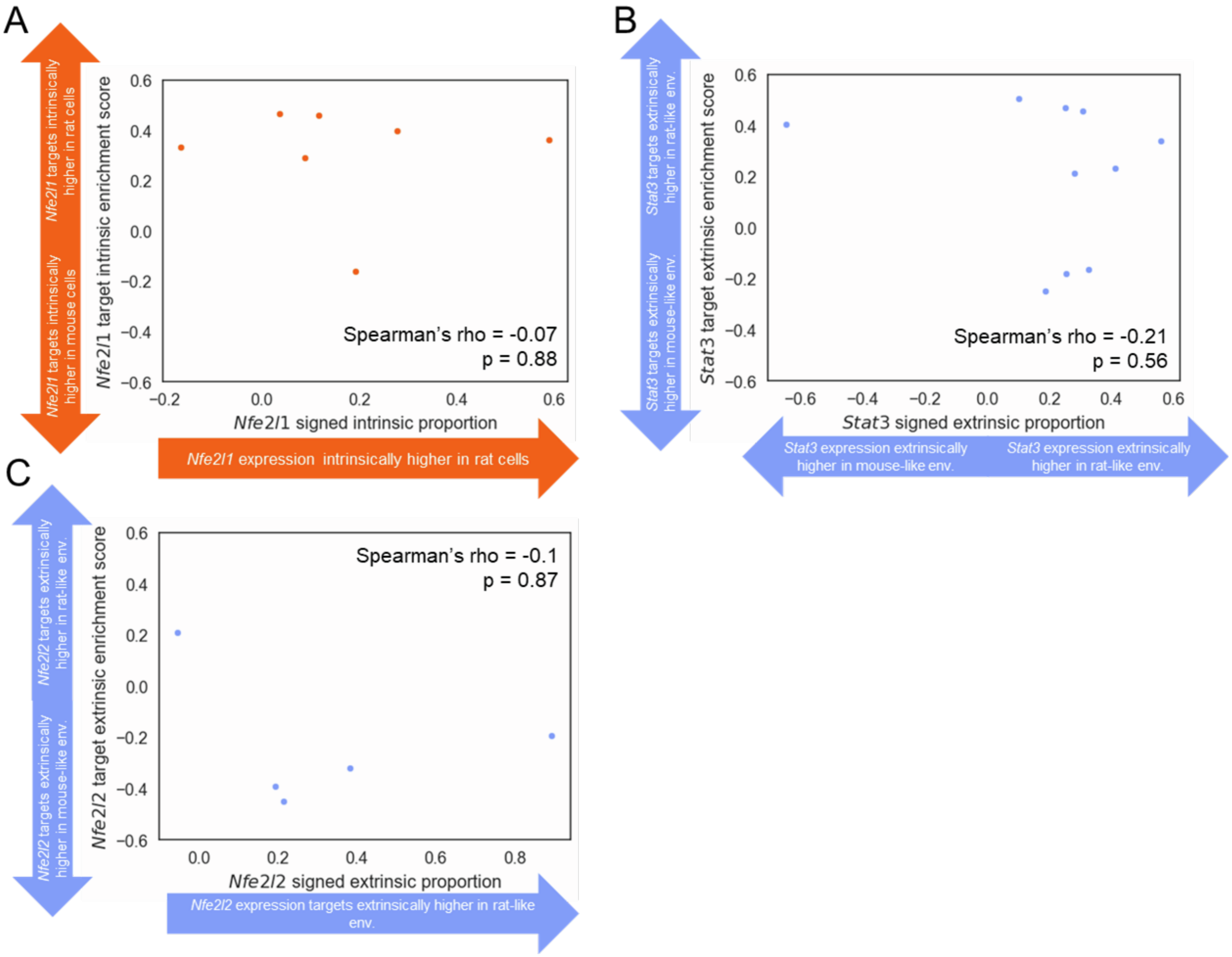

Fig. S20: Analysis of TFs regulating proteasomal subunit expression and their target genes, related to figure 5. A) Scatter plot showing the relationship between signed intrinsic proportion divergence for *Nfe2l1* (x-axis) and the enrichment of its target genes for signed intrinsic proportion divergence (y-axis). Each point is a cell type and cell types with intrinsically driven increased expression of *Nfe2l1* in rat cells have larger values on the x-axis. Cell types with intrinsically driven increased expression of *Nfe2l1* target genes in rat cells have larger values on the y-axis. **B)** Correlation between signed extrinsic proportion divergence for *Stat3* (x-axis) and the enrichment of its target genes for signed extrinsic proportion divergence (y-axis). Each point is a cell type and cell types with extrinsically driven increased expression of *Stat3* in a rat-like environment have larger values on the x-axis and cell types with extrinsically driven

increased expression of *Stat3* in a mouse-like environment have smaller values on the x-axis. Cell types with extrinsically driven increased expression of *Stat3* target genes in a rat-like environment have larger values on the y-axis whereas cell types with extrinsically driven increased expression of *Stat3* target genes in a mouse-like environment have smaller values on the y-axis. **C)** Correlation between signed extrinsic proportion divergence for *Nfe2l2* (x-axis) and the enrichment of its target genes for signed extrinsic proportion divergence (y-axis). Each point is a cell type and cell types with extrinsically driven increased expression of *Nfe2l2* in a rat-like environment have larger values on the x-axis and cell types with extrinsically driven increased expression of *Nfe2l2* in a mouse-like environment have smaller values on the x-axis. Cell types with extrinsically driven increased expression of *Nfe2l2* target genes in a rat-like environment have larger values on the y-axis whereas cell types with extrinsically driven increased expression of *Nfe2l2* target genes in a mouse-like environment have smaller values on the y-axis.

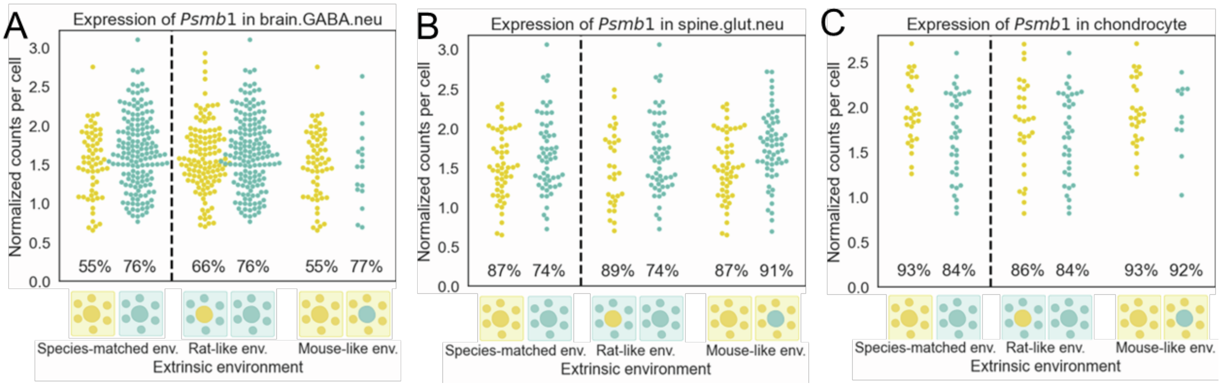

Fig. S21: Per-cell expression of *Psmb1* across cell types, related to figure 5. Each swarm of points shows the normalized counts for a gene in each cell with non-zero counts for that gene. The percentage near the bottom of the plot indicates the percentage of cells with non-zero counts for that gene. **A)** Per-cell expression *Psmb1* in forebrain GABAergic neurons. **B)** Per-cell expression of *Psmb1* in spinal glutamatergic neurons. **C)** Per-cell expression of *Psmb1* in chondrocytes.

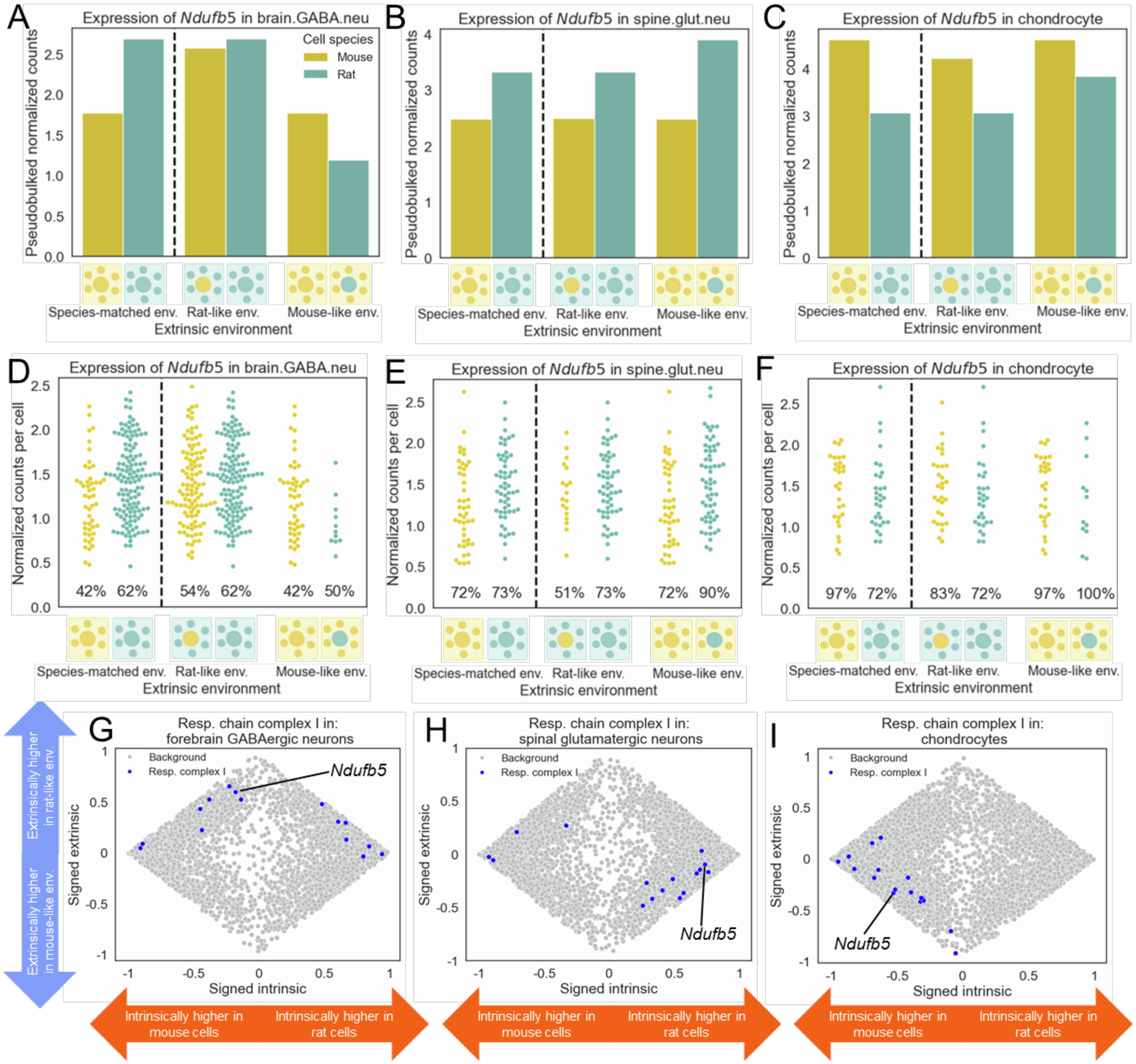

Fig. S22: Cell-type specific intrinsic and extrinsic divergence of the expression of genes encoding mitochondrial respiratory chain complex I subunits across cell types, related to figure 5. A) Expression of *Ndufb5* in forebrain GABAergic neurons. **B)** Same as in (A) but for spinal glutamatergic neurons. **C)** Same as in (A) but for chondrocytes. **D)** Per-cell expression *Ndufb5* in forebrain GABAergic neurons. Each swarm of points shows the normalized counts for a gene in each cell with non-zero counts for that gene. The percentage near the bottom of the plot indicates the percentage of cells with non-zero counts for that gene. **E)** Per-cell expression

of *Ndufb5* in spinal glutamatergic neurons. **F)** Per-cell expression of *Ndufb5* in chondrocytes. **G)** Scatterplot showing signed proportion intrinsic divergence (x-axis) and signed proportion extrinsic divergence (y-axis) for all genes passing our filtering criteria for forebrain GABAergic neurons. Genes coding for mitochondrial respiratory chain complex I subunits are shown in blue and all other genes are shown in grey. Expression of genes coding for mitochondrial respiratory chain complex I subunits is generally extrinsically higher in a rat-like environment. **H)** Same as in (G) but for spinal glutamatergic neurons. Expression of genes coding for mitochondrial respiratory chain complex I subunits is generally intrinsically higher in rat cells but extrinsically higher in a mouse-like environment. **I)** Same as in (G) but for chondrocytes. Expression of genes coding for mitochondrial respiratory chain complex I subunits is generally intrinsically higher in mouse cells and extrinsically higher in a mouse-like environment.

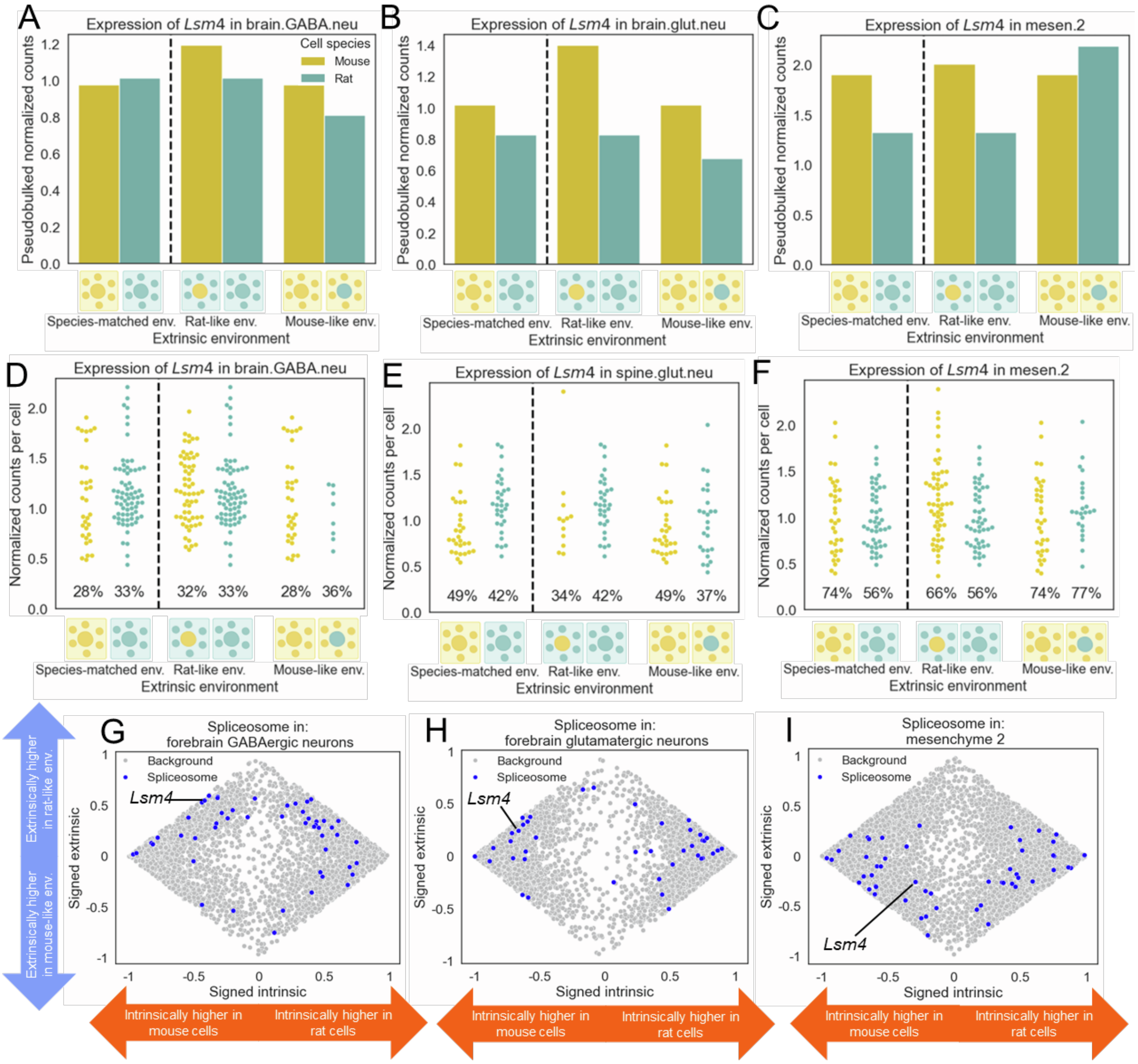

Fig. S23: Cell-type specific intrinsic and extrinsic divergence of the expression of genes encoding spliceosomal subunits across cell types, related to figure 5. A) Expression of *Lsm4* in forebrain GABAergic neurons. **B)** Same as in (A) but for spinal glutamatergic neurons. **C)** Same as in (A) but for mesenchymal cluster 2 cells. **D)** Per-cell expression *Lsm4* in forebrain GABAergic neurons. Each swarm of points shows the normalized counts for a gene in each cell with non-zero counts for that gene. The percentage near the bottom of the plot indicates the percentage of cells with non-zero counts for that gene. **E)** Per-cell expression of *Lsm4* in spinal glutamatergic neurons. **F)** Per-cell expression of *Lsm4* in mesenchymal cluster 2 cells. **G)**

Scatterplot showing signed proportion intrinsic divergence (x-axis) and signed proportion extrinsic divergence (y-axis) for all genes passing our filtering criteria for forebrain GABAergic neurons. Genes coding for spliceosomal subunits are shown in blue and all other genes are shown in grey. Expression of genes coding for spliceosomal subunits is generally extrinsically higher in a rat-like environment. **H)** Same as in (G) but for forebrain glutamatergic neurons. Expression of genes coding for spliceosomal subunits is generally extrinsically higher in a mouse-like environment and intrinsically higher in rat cells. **I)** Same as in (G) but for mesenchymal cluster 2 cells. Expression of genes coding for spliceosomal subunits is generally extrinsically higher in a mouse-like environment and intrinsically higher in mouse cells.

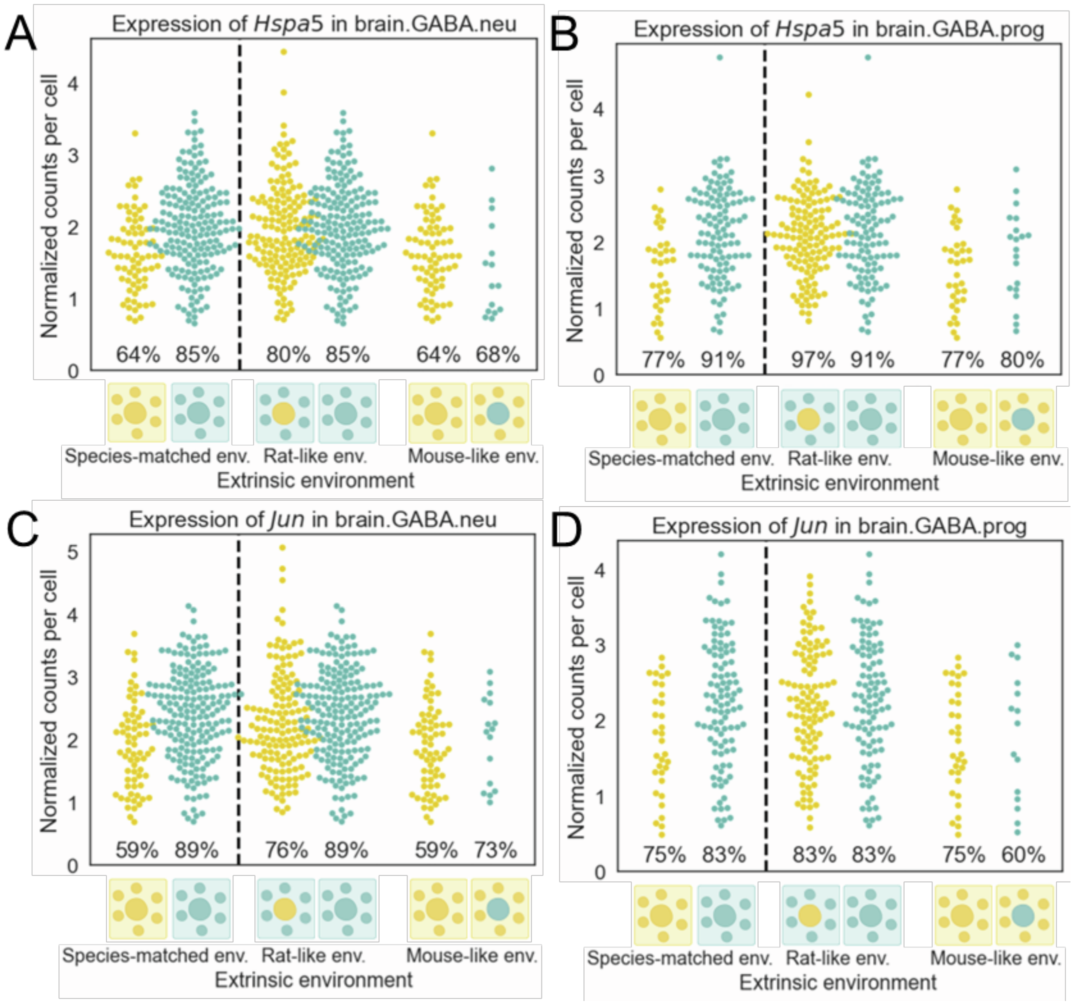

Fig. S24: Per-cell expression of *Jun* and *Hspa5* in forebrain GABAergic neurons and progenitors, related to figure 6. Each swarm of points shows the normalized counts for a gene in each cell with non-zero counts for that gene. The percentage near the bottom of the plot indicates the percentage of cells with non-zero counts for that gene. **A)** Per-cell expression of *Hspa5* in forebrain GABAergic neurons. **B)** Per-cell expression of *Hspa5* in forebrain GABAergic progenitors. **C)** Per-cell expression of *Jun* in forebrain GABAergic neurons. **D)** Per-cell expression of *Jun* in forebrain GABAergic progenitors.

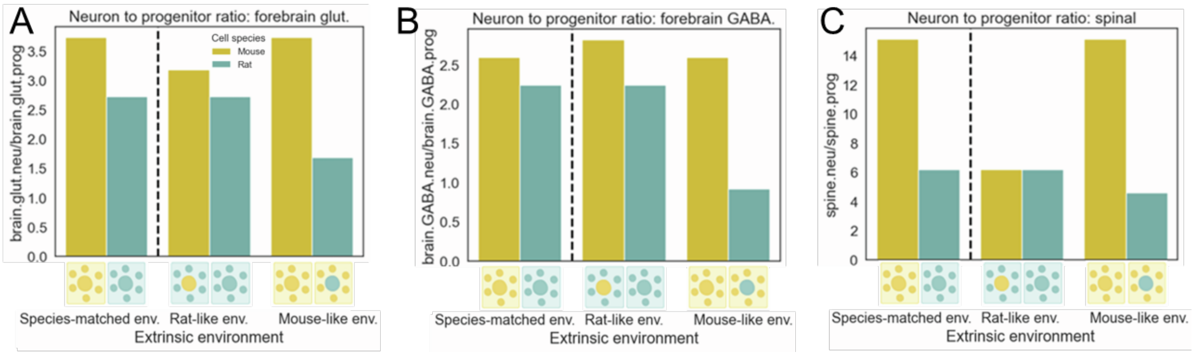

Fig. S25: Neuron-to-progenitor ratio in different neurogenic niches, related to figure 6. A) Plot showing the neuron-to-progenitor ratio for forebrain glutamatergic neurogenesis across the four different species-environment combinations. **B)** Same as in (A) but for forebrain GABAergic neurogenesis. **C)** Same as in (A) but for combined GABAergic and glutamatergic spinal neurogenesis. The two lineages were combined as we were unable to distinguish GABAergic and glutamatergic progenitors.

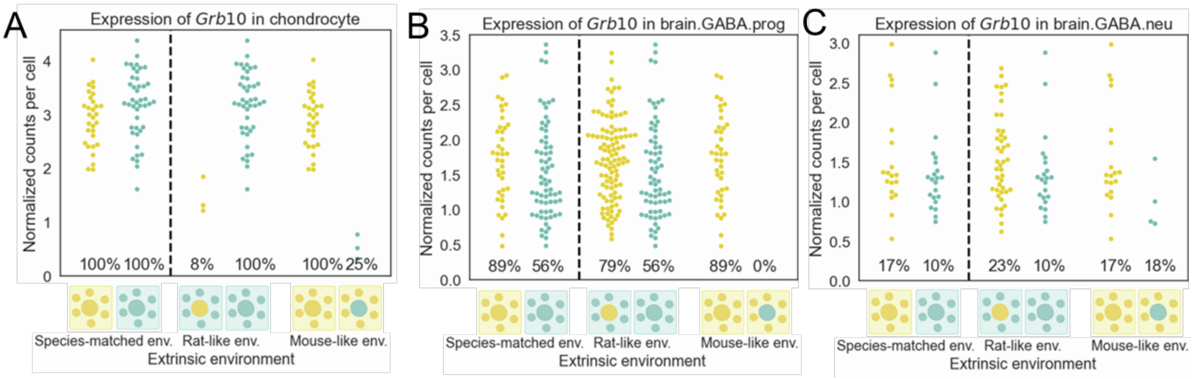

Fig. S26: Per-cell expression of *Grb10* across cell types, related to figure 7. Each swarm of points shows the normalized counts for a gene in each cell with non-zero counts for that gene. The percentage near the bottom of the plot indicates the percentage of cells with non-zero counts for that gene. **A)** Per-cell expression of *Grb10* in chondrocytes. **B)** Per-cell expression of *Grb10* in forebrain GABAergic progenitors. **C)** Per-cell expression of *Grb10* in forebrain GABAergic neurons.

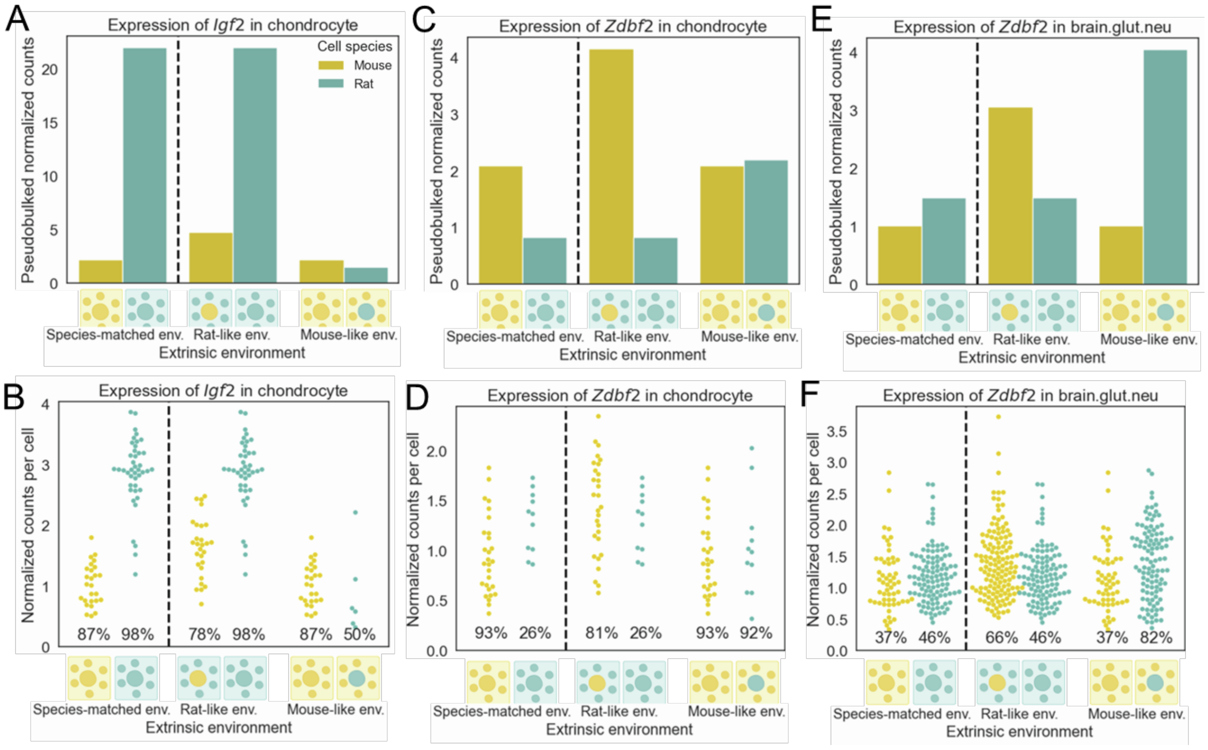

Fig. S27: Expression of selected imprinted genes, related to figure 7. A) Expression of *Igf2* across the four species-environment combinations in chondrocytes. Expression is very high for rat cells in a rat-like environment but is very low for rat cells in a mouse-like environment. **B)** Per-cell expression *Igf2* in chondrocytes. Each swarm of points shows the normalized counts for a gene in each cell with non-zero counts for that gene. The percentage near the bottom of the plot indicates the percentage of cells with non-zero counts for that gene. **C)** Expression of the imprinted gene *Zdbf2* across the four species-environment combinations in chondrocytes. Expression is higher in species-mismatched environments in cells from both species. **D)** Per-cell expression of *Zdbf2* in chondrocytes. **E)** Expression of the imprinted gene *Zdbf2* in forebrain glutamatergic neurons across the four species-environment combinations in forebrain glutamatergic neurons. Expression is higher in species-mismatched environments in cells from both species. **F)** Per-cell expression of *Zdbf2* in forebrain glutamatergic neurons.

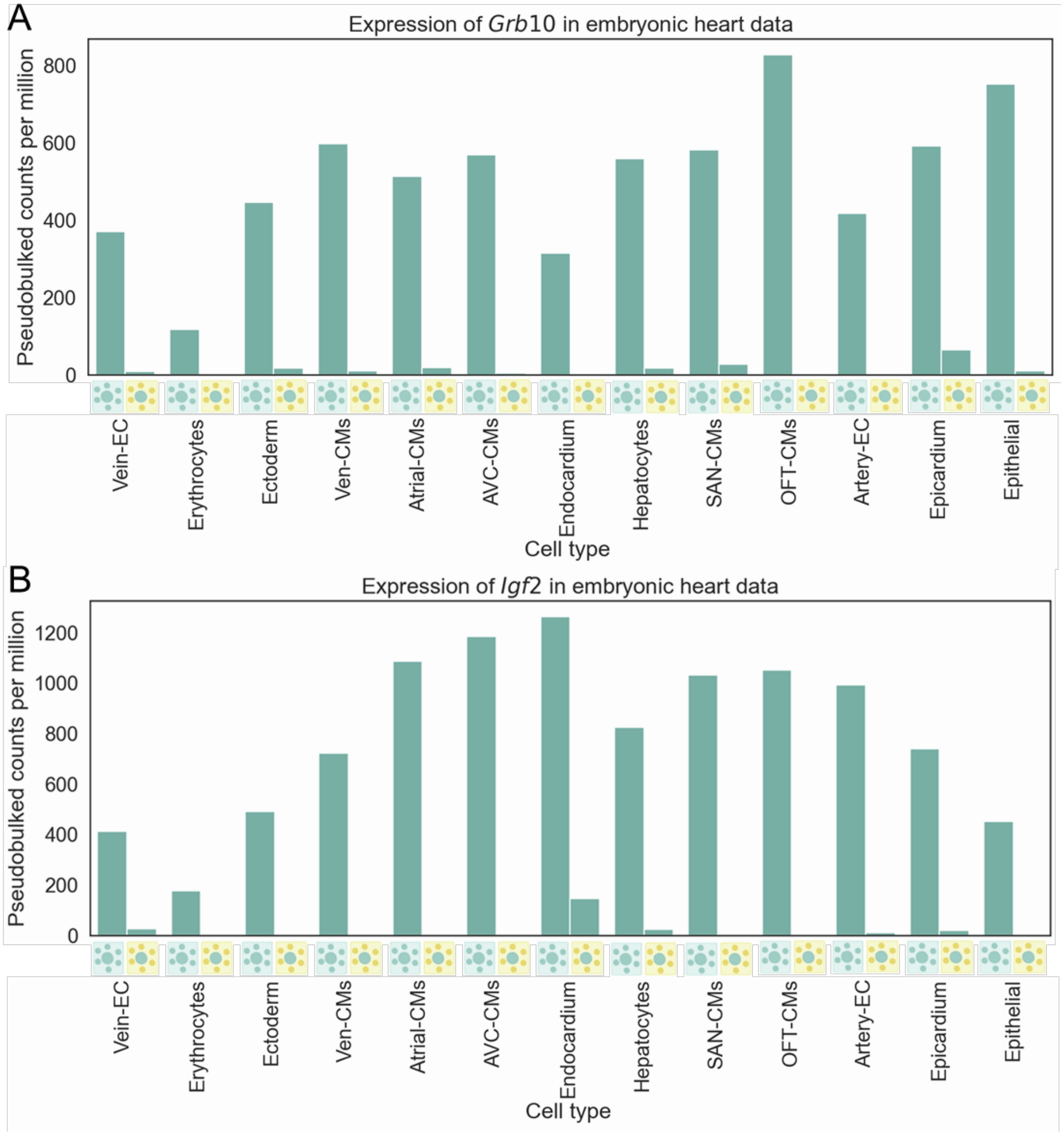

Fig. S28: Expression of imprinted genes *Grb10* and *Igf2* across cell types from the heart dataset, related to figure 7. For each cell type, expression in rat cells in a rat-like environment is shown on the left and expression in rat cells in a mouse-like environment is shown on the right. **A)** Expression of *Grb10* across cell types. Expression is higher in a species-matched

environment. **B)** Expression of *Igf2* across cell types. Expression is higher in a species-matched environment.

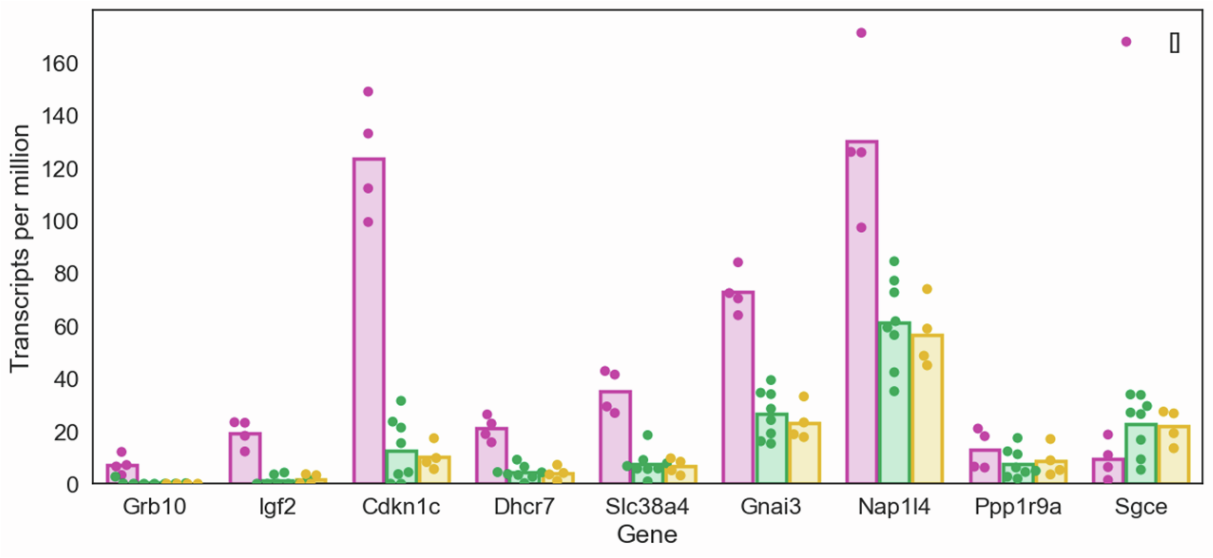

Fig. S29: Disrupted expression of imprinted genes in adult mouse parathyroid cells in species-mismatched environments, related to figure 7. A) Expression levels of mis-expressed imprinted genes for samples from species-mismatched environments, species-matched environments, and wildtype mice. Expression is higher in species-mismatched environments, but very similar between donor mouse cells in species-matched environments and wildtype mice. All imprinted genes with absolute difference between expression for species-mismatched and species-matched environment samples greater than 1 were plotted.

	Cell line	Embryo strain	Number of cells injected into embryo	stage (Embryonic)	No. of neonates	No. of chimeras
					(%)	(%)
mouse ESCs	CD1 (SUN106.2)	Wistar rat	5-7	13.5	23 (37)	7 (30)
	C57BL/6 (SGE2)	Wistar rat	5-7		23 (44)	16 (70)
Rat ESCs	Wistar (rat ESC No.3)	CD1 mouse	5-7	15.25	19(19)	10(53)
		C57BL/6 mouse	5-7		7(7)	1(14)

Table S4, related to STAR Methods: Results of all embryo injections performed.

			Donor chimerism analyzed by Flow cytometry as a percentage, (scRNA-seq library)		
Chimera	Donor cell	Host embryo	Chimera No.	Connective tissue	Forebrain
Mouse to Rat	SUN106.2	Wistar	1	23.1	1.84 (MR1)
			2	4.76 (MR3)	0
			3	0.95	0.03
			4	22.7	1.02 (MR2)
			5	15.4	0.64 (MR3)
			6	4.29 (MR2)	0
			7	3.52 (MR1)	0.089
	SGE2	Wistar	1	N/A	2.25
			2	2.22	11.5
			3	1.47	4.1
			4	0.31	3.48
			5	1.76	8.56
			6	0.15	1.86
			7	0.53	10.4
			8	3.92	21
			9	2.09	7.72
			10	0.078	1.56
			11	0.27	2.26
			12	0.45	4.33
			13	0.11	0.14
			14	0.88	0.56
			15	0.073	1.91
			16	0.6	8.81
Rat to Mouse	rat ESC No.3	C57BL/6	1	12.9	24.8
	rat ESC No.3	CD1	1	9.69	19.2
			2	5.59 (RM1)	3.14
			3	12.2	14
			4	4.52 (RM2)	9.22
			5	7.6 (RM3)	5.59
			6	2.27	1.81(RM2)
			7	4	3.23 (RM3)
			8	5.15	6.39 (RM1)
			9	8.75	15.9

Table S5, related to STAR Methods: Donor percentages for all chimeras analyzed with flow cytometry. The abbreviation in the parentheses indicate the library to which the tissue contributed.
